# Biological marks of early-life socioeconomic experience is detected in the adult inflammatory transcriptome

**DOI:** 10.1038/srep38705

**Published:** 2016-12-09

**Authors:** Raphaële Castagné, Michelle Kelly-Irving, Gianluca Campanella, Florence Guida, Vittorio Krogh, Domenico Palli, Salvatore Panico, Carlotta Sacerdote, Rosario Tumino, Jos Kleinjans, Theo de Kok, Soterios A. Kyrtopoulos, Thierry Lang, Silvia Stringhini, Roel Vermeulen, Paolo Vineis, Cyrille Delpierre, Marc Chadeau-Hyam

**Affiliations:** 1 Department of Epidemiology and Biostatistics, School of Public Health, Imperial College London, Norfolk Place, W2 1PG London, UK; 2 INSERM, UMR1027, Toulouse F-31000, France; 3 Université Toulouse III Paul-Sabatier, UMR1027, Toulouse F-31000, France; 4 Epidemiology and Prevention Unit, Fondazione IRCCS- Istituto Nazionale dei Tumori, Via Venezian 1, 20133 Milan, Italy; 5 Molecular and Nutritional Epidemiology Unit, Istituto per lo Studio e la Prevenzione Oncologica (ISPO Toscana), Via delle Oblate 2, 50141, Florence, Italy; 6 Department of Clinical Medicine and Surgery, University of Naples Federico II, Via Pansini 5, 80131 Naples, Italy; 7 Piedmont Reference Centre for Epidemiology and Cancer Prevention (CPO Piemonte), Viale Settimio Severo nr. 65, 10133 Turin, Italy; 8 Cancer registry and Histopathology Unit, Azienda Ospedaliera ‘Civile –M.P.Arezzo’, Via Dante N 109, 97100 Ragusa, Italy; 9 Department of Toxicogenomics, Maastricht University, 6211 LK Maastricht, The Netherlands; 10 National Hellenic Research Foundation, Institute of Biology, Pharmaceutical Chemistry and Biotechnology, Vas. Constantinou 48, 11635 Athens, Greece; 11 Institute of Social and Preventive Medicine, Lausanne University Hospital, Route de la Corniche 10, 1010 Lausanne, Switzerland; 12 Institute for Risk Assessment Sciences (IRAS), Utrecht University, PO Box 80178, 3508 TD, Utrecht, The Netherlands; 13 HuGeF, Human Genetics Foundation, Via Nizza 52, 10126 Torino, Italy; 14 MRC-PHE Centre for Environment and Health, Imperial College, Praed Street Wing, St Mary’s Campus, W2 1PG London, UK

## Abstract

Consistent evidence is accumulating to link lower socioeconomic position (SEP) and poorer health, and the inflammatory system stands out as a potential pathway through which socioeconomic environment is biologically embedded. Using bloodderived genome-wide transcriptional profiles from 268 Italian participants of the European Prospective Investigation into Cancer and Nutrition (EPIC) cohort, we evaluated the association between early life, young and later adulthood SEP and the expression of 845 genes involved in human inflammatory responses. These were examined individually and jointly using several inflammatory scores. Our results consistently show that participants whose father had a manual (as compared to nonmanual) occupation exhibit, later in life, a higher inflammatory score, hence indicating an overall increased level of expression for the selected inflammatory-related genes. Adopting a life course approach, these associations remained statistically significant upon adjustment for later-in-life socioeconomic experiences. Sensitivity analyses indicated that our findings were not affected by the way the inflammatory score was calculated, and were replicated in an independent study. Our study provides additional evidence that childhood SEP is associated with a sustainable upregulation of the inflammatory transcriptome, independently of subsequent socioeconomic experiences. Our results support the hypothesis that early social inequalities impacts adult physiology.

Life course socioeconomic position (SEP), whether assessed by income, education or occupation, is linked to a wide range of adverse health conditions, including cardiovascular disease, hypertension, diabetes and cancer^1^. The literature suggests that socioeconomic disadvantage tends to yield poorer health^2^,^3^, and while behavioural and lifestyle factors are important determinants of mortality, several epidemiological studies have shown that social inequalities cannot fully be explained by established risk factors for chronic disease^4^,^5^. There are various physiological systems (parasympathetic and sympathetic nervous system; hypothalamic-pituitary-adrenal axis) through which a wide range of possible mediators at different molecular levels (hormones, proteins, gene expression and DNA methylation) may contribute to the biological embedding of social experiences^6^,^7^,^8^,^9^. One of the leading mechanistic hypotheses about how SEP exerts its effects on health is inflammation^6^. Chronic psychosocial stress can alter the body’s ability to regulate the pathways of inflammation over time^10^ and promote the development and progression of many chronic health conditions including cardio-metabolic and neurodegenerative disorders, asthma and cancer^11^,^12^.

Research shows that in adults, low SEP is associated with (i) specific inflammatory profiles, such as elevated C-reactive and interleukin 6 proteins^13^,^14^,^15^,^16^,^17^, and (ii) a composite inflammatory score based on circulating levels of 28 inflammation-related proteins^18^. Consistently, with the rapid development of field of social genomics^19^, focusing on the identification of molecular signals involved in the regulation of gene activity in response to social experiences, evidence is accumulating to show that adverse life circumstances such as low SEP^20^,^21^, social isolation^22^,^23^ and early-life social deprivation^24^ are related to the up-regulation of the expression of several genes involved in the inflammatory pathways^25^. The pattern of social stress-related gene expression has been formalised as a Conserved Response To Adversity (CTRA)^26^ which encompasses 53 genes. The latter is characterised by the differential expression of genes involved in inflammation, antiviral responses and antibody synthesis that have been developped during evolution to respond to stressful environment in both humans and animal models^27^,^28^,^29^.

In the present study we extend that definition of the inflammatory transcriptome, and exploit genome-wide gene expression profiles measured from peripheral blood mononuclear cells (PBMC) prospectively collected in 268 participants from the Italian component of the European Prospective Investigation into Cancer and Nutrition (EPIC-Italy), using the Agilent 4 × 44 K human whole genome microarray technology. We used a comprehensive panel of genes (N = 1,027) involved in various aspects of inflammatory responses^30^. Of these, a vast proportion (N = 845) was assayed in our study population and were used to define the inflammatory transcriptome. In a first approach, each of the contributing genes was examined separately. In accordance with the theory of a global wear-and-tear due to stressful events on the body^31^, we assumed a global positive association between inflammation (as indicated by an upregulation of inflammatory genes) and lower SEP to define a composite inflammatory score, which was complemented by a continuous and a rank-based alternative. Irrespective of the measure used, the inflammatory transcriptome was examined in relation to SEP at different life stages: early-life, young adulthood and adulthood. As previously proposed^18^, we investigated life course effects of early-life SEP experiences by sequentially controlling for time-ordered SEP. In order to assess the robustness of our findings, we conducted a series of sensitivity analyses and independent replication was sought for by using a publicly available dataset. Finally to gain better understanding of the inflammatory response to SEP experiences we investigated our inflammatory scores for several (functionally-defined) inflammatory sub-pathways^30^.

## Results

A detailed description of the study population is given in Table 1. Irrespective of the SEP indicator, participants in the low SEP group tend to be older, to have a higher BMI, and to exhibit a lower smoking prevalence than those in higher SEP categories.

### Inflammatory transcriptome of life course SEP indicators

We first investigated associations between each of the 845 genes and the three time-specific SEP indicators (father’s occupational position, participant’s education, highest occupational position in the household). For all indicators and in all subsequent analyses, the ‘high’ socioeconomic group was used as reference and a positive association therefore indicates an up regulation of gene expression in the ‘low’ socioeconomic group. No association reached statistical significance after correcting for multiple testing with father’s occupational position, education and highest household occupational position (Table S1 and Figures S1–S3).

### Inflammatory transcriptome scores and life course SEP indicators

**Figure 1:**
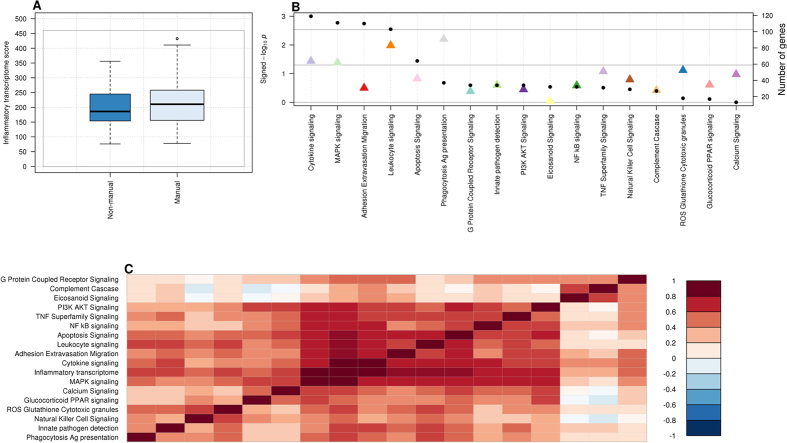
(**A**) Boxplot of the inflammatory transcriptome for both classes of father’s occupational position. (**B**) Association between sub-pathway inflammatory score and father’s occupational position (model A). The −log10 p-value (left Y-axis) is signed by the direction of the effect size estimate and is given separately for of the 17 sub-pathways (X-axis). The dotted horizontal line represents the Bonferroni significance level correcting for 17 tests, and ensuring a family wide error rate of 5%. In the secondary (right) Y-axis, the number of genes contributing to each sub-pathway is represented. (**C**) Pairwise Spearman correlation between each of the 17 sub-pathway scores in the EPIC-Italy participants (N = 246).

As detailed in the methods section, we defined a transcriptome inflammatory score by cumulating over all 845 selected inflammatory-related genes a binary indicator indicating for each gene, low (first three quartiles) or high (highest quartile) expression levels^32^. Only father’s occupational position was found significantly associated with this inflammatory transcriptome score (Table 2): participants whose father had a low occupational position had a higher inflammatory score (*β* = 21.81, P = 0.04, Fig. 1A). We also identified for participants with lower household occupational position a higher inflammatory score (*β* = 7.51, P = 0.49), and conversely, the score was lower in participants with lower educational level (*β* = −0.64, P = 0.95). However, none of these two associations were statistically significant (P > 0.49, Table 2). The association between low early-life SEP and higher inflammatory transcriptome score (Table 3A, model A) was slightly strengthened by adjusting for participant’s education (Table 3A, model B, P = 0.02), and subsequently for highest household occupational position (Table 3A, model C, P = 0.03). Adjustments for potential (behavioural) confounders only marginally impacted these associations (Table 3A, model D, P = 0.03). Further adjustment on the estimated cell types proportions (see Methods, neutrophils and monocytes), slightly weakened the association with father’s occupational position, which however, remained statistically significant (Table 3A, fully adjusted model, P-value = 0.040).

### Sensitivity analyses

To assess the specificity of the 845 genes selected to define the inflammatory transcriptome, we randomly sampled (N = 10,000) subsets of 845 genes to be included in the score definition and assessed the strength of the association between that ‘null’ score and father’s occupational position. Over the 10,000 random gene sets, only 27 gave rise to a p-value lower than that observed using the real score. The stability of our findings to the definition of the inflammatory score was also investigated by considering two alternative definitions: (i) the first principal component from the 845 gene expression levels (PC1, explaining 36.8% of the total variance), and (ii) a cumulative gene ranking-based score recently proposed by Sood *et al*.^33^. Because of the negative correlation between the inflammatory score and PC1 (*ρ* = −0.53, P < 0.001), a higher PC1 score indicates lower inflammatory transcriptome score level and effect size estimates have reversed signs. Although slightly weakened in terms of strength of association, our results and conclusions remained markedly stable irrespective of the score considered: we identified significant association between father’s occupational position and both inflammatory PC1/rank score (Table 2), that remained stable after controlling for young and adult SEP (Table 2B,C) and a non-significant association with participant’s education and highest household occupational position (Table 1).

### External validation

To provide additional support for the association between the inflammatory transcriptome score, we used a publicly available dataset (GSE15180) which included gene expression profiles from PBMCs in 30 adults with low early-life SEP and 30 adults with high early-life SEP from Vancouver, Canada (ages 25–40 years)^24^. Several potentially differences between the validation and our original study population include country, age and study design. Despite these specificities, we were able to replicate our main findings in that independent population (Table 4) and found that participants whose parents had a low-SEP household had a higher inflammatory transcriptome score (*β* = 24.50, P = 0.02). Sensitivity analyses using the PC1 (explaining 13.5% of the variance) and the cumulative gene ranking-based score resulted in a stronger association for the cumulative gene-ranking score (Table 4).

### Inflammatory sub-pathways scores

To try and capture the multiplicity and complexity of biological mechanisms involved in the inflammation responses, we analysed 17 sub-pathways^30^, and defined for each of them a specific score using the same approach. As summarised in Table 5, we examined the association between father’s occupational position and each sub-pathway score. We found that father’s occupational position was positively correlated to all 17 sub-pathway scores, suggesting a general and non-specific stimulation of the inflammatory sub-pathway in the ‘low’ socioeconomic group (Fig. 1B). None of these associations survived a stringent Bonferroni correction, but the two strongest ones involved the Phagocytosis-Antigen presentation sub-pathway (N = 37 genes, *β* = 2.13, P-value = 0.006), and the Leukocyte signalling pathway (N = 103 genes, *β* = 3.62, P-value = 0.010) (Fig. 1B). Those associations remained nominally significant after controlling for education and upon adjustment for highest household occupational position and potential confounders. Positive and strong pairwise correlation coefficients were observed across the 17 sub-pathways scores (Fig. 1C). This indicates the functional proximity of these sub-pathways all contributing to the inflammatory responses and further supports the global wear-and-tear hypothesis underlying the definition of our overall inflammatory transcriptome score.

## Discussion

In the present study, we investigated the association between SEP at different time points in life and the inflammatory transcriptome using a panel of 845 inflammatory genes considered separately, or combined into an inflammatory transcriptome score. We hypothesised that SEP may physiologically be embedded from early life, and subsequently affect the inflammatory burden. Testing each inflammatory-related genes in relation to the three SEP indicators separately, we did not identify any significant association after correction for multiple testing, which could be related to the limited statistical power our population size yields. Formal power calculations indicated that a sample size group of around 600 subjects was required to yield a power of 80% to detect the strongest effect size we estimated. In that context, and in order to limit the number of tests performed (and hence preserve power), individual inflammatory status was defined using an inflammatory transcriptome score. These analyses indicated that participants reporting a father with a ‘manual’ occupation had a higher inflammatory transcriptome score later in life compared to those whose father had a ‘non-manual’ occupation. This association was robust to the definition of the inflammatory score, was not affected by the adjustment for the main potential confounders and was not driven by differential blood cell composition. No significant association was found between participant’s education and highest household occupation. Our results suggest that early-life SEP is associated with elevated expression of inflammatory-related genes in adulthood. This result was further strengthened by external validation in an independent and publicly available dataset. We assessed the contribution of each functional pathways involved in the inflammation responses. Although none of the 17 pathways was statistically significant after multiple testing correction, we identified 4 sub-pathways that were significantly overexpressed at a nominal 5% significance level. While strongest associations included some of the most represented sub pathways (leukocyte, cytokine and MAPK signalling pathways including over 100 genes), some sub-pathways including 40 or less genes (e.g. Phagocytosis Antigen presentation) were also identified.

Several limitations of this study should be considered. First, our study population remains limited in size, which constrained our methodological choices: the categories for the three life course SEP were all recoded (binary indicators) to preserve statistical power. The use of binary SEP indicators to capture complex and multivariate SE experiences and related exposures over a long period of time may hamper the performance of our model through an inflation of the variability within each SEP category. In addition, our study being based a prospective cohort, data, and in particular father’s occupational position, collected at enrolment may be subject to recall bias. Further, there was no information available on potential participant’s infection at enrolment. Our study population includes participants from one breast cancer and one lymphoma nested case control study and may therefore lack of representativeness. Our study population includes a large proportion of breast cancer prospective cases-control pairs. Given the higher breast cancer incidence in higher SE categories, our results may be prone to residual confounding and cannot directly be generalised to other populations.

We replicated our results in an independent study comprising participants of different age range, country, and socioeconomic background than those from our study population. In addition, gene expression data from the replication set arose from a different technology (Illumina HumanRef-8 v3.0 Expression Beadchips). Despite these sources variability, we were able to replicate the association between the inflammatory score and early-life exposure to socioeconomic disadvantages. Altogether, this supports the generalisability of our findings. Nevertheless, we cannot exclude the possibility that other genes and/or pathways may also be involved in the biological embedding of early-life SEP.

To test the robustness of the definition of the inflammatory transcriptome score, we defined, as an unsupervised alternative, the inflammatory score as the first principal component (PC1) obtained from the (N = 845) inflammatory-related genes and also considered a rank-based approach recently proposed by Sood *et al*.^33^. We identified consistent, though statistically weakened, associations with father’s occupation after controlling for young and adult SEP. This consistency may at least be partially explained by the strong correlation between our inflammatory score and both PC1 and the rank-based score (*ρ* = −0.53 and 0.56, respectively). Our estimates and conclusions remained stable upon adjustments for behavioural factors (smoking and alcohol consumption, and BMI) and cell sub-populations, hence providing evidence that the inflammatory signal we report as markers of SEP are independent of the potential inflammatory signatures of these factors.

Our results are consistent with previous findings: a number of published studies linked various indicators of SEP with circulating inflammatory marker^34^,^35^. Several studies have examined the influence of childhood SEP (as measured by father’s occupation or father’s education) on inflammatory protein concentration in adulthood; specifically, these have shown that lower early-life SEP was associated with a greater level of C Reactive Protein^17^,^36^,^37^, fibrinogen^17^,^36^,^37^ and interleukin 6^17^,^38^.

The transcriptional dynamic was also investigated in previous research suggesting that negative experiences in early-life are associated with increased inflammatory gene expression in adulthood by examining both childhood and adult stress and their associations with later life gene expression^6^,^20^,^24^,^25^,^39^,^40^. The replication dataset used in our study was initially designed to compare healthy adults matched on current SEP who came from high- versus low-SEP families of origin. The original research work revealed that early-life low SEP was associated with resistance to glucocorticoid signalling, which in-turn promoted adrenocortical and inflammatory responses through the transcriptional dynamics and the immune activation^24^.

Building upon an approach we developed in a lower dimensional setting^18^, the present work proposes an operationalization of the inflammatory transcriptome through different score metrics allowing its applicability to a broad spectrum of cohort studies or tissues and cell types. The originality of this study not only resides in the extension, at the gene expression level, of previous results observed using targeted protein profiles, but also provides insights, from two independent study populations, into the way social experiences are biologically translated in the long-term. In particular, our study provides evidence that early life SEP affects in the long-term the inflammatory transcriptome.

While our study provides consistent evidence of the sustainable inflammatory response to early-life adversity, further work would be required to identify the molecular processes triggering and maintaining these molecular signals and to explore the functional consequences of the observed increased inflammatory transcriptome score. One candidate mechanism might involve epigenetic alterations as DNA methylation and histone modification play crucial roles in development, adaptation and response to environmental signals^41^. Several recent studies have supported the possibility of differential DNA methylation patterns in peripheral blood cells associated with factors describing the individual social environment^42^,^43^,^44^. In a previous study using participants from EPIC-Italy, we reported that indicators of life course SEP were associated with DNA methylation levels in genes (N = 17 genes, corresponding to 403 CpG sites) involved in inflammation^44^.

Our work highlights research questions needed to explore the mechanisms involved in the social-to-biological transition over the life course. One way forward would be to exploit existing resources where multiple OMIC profiles are available in the same individuals and to identify the main regulatory cascades that are triggered and biologically mediate the effect socioeconomic experiences during the life course. While this could be done in a targeted way, typically looking at the correlation patterns existing across molecular signals arising at different cellular level, a hypothesis-free investigation would require larger studies with repeated data on social circumstances and biological molecular profiles over the life span. Understanding biological mechanisms by which social environment influences the inflammatory system has important implications in treatment and especially in prevention, by potentially identifying modifiable factors in the environment that affect physiological health.

## Methods

### Study population

Our study population arises from the EnviroGenoMarkers (EGM) project, which was initially designed to identify novel biomarkers of non-Hodgkin’s lymphoma and breast cancer risk from multiple ‘-omics’ profiles^45^. We include in this study 268 EGM participants from the Italian component of EPIC who were healthy (i.e. cancer-free) at enrolment. For each participant, one blood sample as well as questionnaire-based anthropometric, lifestyle, dietary and socioeconomic factors were collected at baseline^46^. All participants provided informed consent, and the EPIC study protocol was approved by the review board of the International Agency for Research on Cancer and by all local institutes recruiting participants. The study was conducted in accordance with the approved guidelines. Incident NHL (N = 84) and breast cancer (N = 50) cases were diagnosed between 2 and 13 years after recruitment in EPIC, and were identified through local cancer registries. For each case identified, one random control was selected among all EPIC Italy participants alive and free of cancer at the time of diagnosis of the index case, matched by centre (Turin; Varese; Naples; Ragusa and Florence), gender, date of blood collection (+/− 6 months), and age at recruitment (+/− 2.5 years). Biosamples underwent genome-wide expression profiling in two distinct phases including 100 and 34 case/control pairs, respectively.

### Life course socioeconomic position

To preserve power and interpretability, SEP factors from the EPIC questionnaire were dichotomised. Childhood SEP was measured by father’s occupation and recoded in the two following categories; i) ‘Manual’ (N = 144) consisting of: unskilled workers (N = 52), skilled workers (N = 57), and farmers (N = 35); and ii) ‘Non-manual’ (N = 82) consisting of: retailers (N = 28), employees (N = 39), and self-employed (N = 15). Young adulthood SEP was measured through participant’s own education which was dichotomised as i) ‘High’ (above the minimum legal education level, 15 years of age; N = 108): professional (N = 29), upper secondary school (N = 51), and university (N = 28); and ii) ‘Low’ (below the minimum legal education level; N = 137): none (N = 5), primary school (N = 73), lower secondary school (N = 59). Adulthood SEP was measured using the highest occupational position in the household as defined by either the participant’s own occupation or his/her partner. It was classified as ‘Manual’ (N = 78) and ‘Non-manual’ (N = 151), following the same categorisation as for father’s occupation. Characteristics of the 222 participants with full SEP information are summarised in Table 1.

### Genome-wide expression profile

We recently demonstrated that high-quality RNA can be obtained from stored PBMC samples from the EPIC-Italy^45^. We also showed that samples not cold-stored within 2 h after blood collection had significantly different expression profiles than fresh samples, and therefore only PBMC samples that had been placed in cold storage within 2 h after blood collection were included in the current study. Gene expression profiles were acquired using the Agilent 4 × 44 K human whole genome microarray platform. Technical performance and quality of the microarrays was assessed according to a protocol described previously leaving a total of 29 662 transcripts successfully analysed in 246 samples^45^,^47^.

### Inflammatory transcriptome definition and gene selection

Loza *et al*. assembled 1,027 inflammation related genes using literature survey and Ingenuity pathway analysis(24). Genes were assigned to one of the functional pathways. This gene list was used to extract the inflammation related genes from our study. Using the Agilent 4 × 44 K human annotation, we first selected all annotated transcripts with an ‘Entrez Gene’ identifiers (N = 23,104 transcripts). Data on the probe level was collapsed to genes prior to analysis. When two or more probes were available for the same gene, the most variable probe was selected using the R package WGCNA resulting in 15,613 annotated genes^48^. Out of the 1,027 inflammatory genes, 845 genes (82.3%) were present in our dataset. The inflammatory transcriptome and its sub-pathways are described in Tables S2 and S3 and Figure S4. Initially, we considered all inflammatory gene expression levels separately. As depicted in Figure S5, gene expression levels exhibit a strong pairwise correlation. Principal components analysis (PCA) of these 845 inflammatory genes level showed that 98 principal components (PCs) explained more than 95% of the total variation (Figure S6). The multiple testing corrected significance level accounts for the correlation in the data and is defined as P = 0.05/98 (P = 0.0005). Finally, we defined an inflammatory transcriptome score from the 845 gene expression levels. For each gene, we defined a dichotomised indicator: ‘high gene expression level’ = 1, and ‘low gene expression level’ = 0 based on the highest quartile of the gene expression level, and summed these across the 845 genes^49^. Using the same strategy we calculated sub-pathway specific scores.

### Sensitivity analyses: alternative inflammatory scores

As a continuous alternative to the inflammatory transcriptome score, we used the first axis from a PCA. We also used the score proposed by Sood *et al*. that allowed both feature selection and direction of regulation to be taken into account in the estimation of the inflammatory transcriptome score. It was defined as a cumulative gene ranking based score and calculated using each of the 845 gene expression values^33^. For a given SEP indicator, gene expression levels were ranked in descending and ascending order for down and up-regulated genes, respectively. The median sum of the rank scores was then calculated across all transcripts, and a cumulative gene ranking based score was then derived for each SEP.

### Statistical analyses

Statistical analyses were performed using R v3.1.2. As proposed elsewhere^47^, the per-gene analyses were based on a linear mixed model controlling for technically-induced noise (nuisance variation: isolation, hybridization, and labeling steps) and investigated the relationship between the expression level of each gene and the SEP. The general formulation of the mixed model for sample *i* is


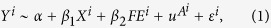


where *Y*^*i*^ represents the gene expression level in participant *i, α* is the intercept, *ε* is the residual error, *X*^*i*^ is the binary SEP indicator observed in that same participant (where the highest class is used as the reference category) whose effect is measured by the regression coefficient *β*_1_, and *FE*^*i*^ is a matrix of fixed effect observations and corresponding regression coefficients are compiled in the vector *β*_2_. Fixed effect covariates include the case-control matching criteria (age, gender, and centre, recoded in three categories: North, Central, and South Italy) and phase. To account for the case-control design of EGM, we also included two binary variables indicating whether a participant is a prospective breast cancer or lymphoma case (model 1). The inflammatory transcriptome score, each of the 17 sub-pathway scores and the principal component 1 (PC1) were based on ‘de-noised’ gene expression levels as obtained from the above linear mixed model by subtracting the random effect estimates from the observed levels. These measures are implicitly corrected for technically-induced variation, and were analysed using a linear model corresponding to equation (1) setting the random intercept term to zero.

### Life course analyses

For the different measures of inflammatory status described above, we used the same benchmark model, and, to mimic life course experiences, we sequentially adjusted for the following chronologically ordered proxies for early-life, young adulthood, and adulthood SEP indicators; resulting in four time-sequenced models:

- Age, gender, case-control status, phase, centre and father’s occupation;
- Model A + education;
- Model B + highest household occupational position;

To control for potential confounder another model was subsequently built upon model C including body mass index (BMI, kg/m2), smoking status (categorical: current, former, and never smoker), and alcohol consumption (g/day) as three potential SEP-driven behaviours (model D). To adjust for potential cell composition bias, we re-tested the model D including two blood cell estimates (neutrophils and monocytes proportion, see below).

### Score specificity

To assess the specificity of the 845 genes constituting the inflammatory transcriptome score, we randomly generated 10,000 independent sets of 845 genes among the 15,613 unique and annotated genes. From each gene set, we calculated the ‘null’ inflammatory transcriptome score based and assessed the strength of their association with father’s occupational position (model 1, see above).

### Replication using a public dataset

The GSE15180 public dataset was used to replicate our findings^24^. Subjects were adult from Vancouver, Canada, from either low or high early lifer SEP background. Early life SEP was measured by parental occupation during the first 5 years of life and was dichotomised as follows: low (manual or lower supervisory occupation) or high (managerial or professional occupation). The authors ensured that both early-life SEP groups had similar average SEP at enrolment and similar demographic (age, sex and ethnicity) and behavioural (smoking, body mass index, alcohol use) characteristics. The raw GSE15180 dataset was downloaded from the GEO database and consisted of 30 adults with low early-life SEP and 30 adults with high early-life SEP who underwent a transcriptional profiling of PBMC using the Illumina HumanRef-5 v3.0 expression beadchip (N = 16 830 transcripts)^24^. Quantile normalization was performed on probe level intensities using the R package preprocessCore^50^. Out of the 1,027 inflammation related genes assembled by Loza *et al*., using the HumanRef-8 v3.0 Expression BeadChip annotation (GPL6883) and the transcript selection described above, 913 (88.9%) inflammatory genes were present in the GSE15180 dataset. The inflammatory transcriptome score was computed as described above together with its 2 alternatives (PC1 and cumulative gene ranked based score). These were linearly regressed against early-life SEP.

### Cell type estimation

PBMC cell composition was assessed by estimating the proportion of each cellular type using the deconvolution algorithm proposed by Abbas *et al*. implemented in the gedBlood function in the CellMix R package^51^,^52^. This resulted in 17 surrogate variables which were then aggregated into ‘Lymphocytes’, ‘Neutrophils’ and ‘Monocytes’ using the asCBC function from the same R package. Applying this method, we found that the EPIC samples contained an average of 75% lymphocytes, 18% neutrophils and 7% of monocytes. These proportions were 71% lymphocytes, 10% neutrophils and 19% monocytes in GSE15180.

## Additional Information

**How to cite this article:** Castagné, R. *et al*. Biological marks of early-life socioeconomic experience is detected in the adult inflammatory transcriptome. *Sci. Rep.* **6**, 38705; doi: 10.1038/srep38705 (2016).

**Publisher's note:** Springer Nature remains neutral with regard to jurisdictional claims in published maps and institutional affiliations.

## Supplementary Material

Supplementary Materials

## Figures and Tables

**Table 1:** Summary characteristics of the study population.

		Father’s occupational position (N = 226)			Participant’s education (N = 245)			Highest household occupational position (N = 229)			Participants with complete data (N = 222)
		Non Manual	Manual	P-value	High	Low	P-value	Non Manual	Manual	P-value	
N		82	144		108	137		151	78		222
Age, yo		52.5 (7.8)	54.2 (8.3)	1.35E-01	50.8 (7.2)	55.4 (8.0)	3.59E-06	52.9 (8)	54.9 (8.2)	8.58E-02	53.6 (8.1)
Gender											
Breast case	Women	62 (75.6)	101 (70.1)	4.67E-01	76 (70.4)	104 (75.9)	4.07E-01	106 (70.2)	57 (73.1)	7.63E-01	160 (72.1)
	Men	20 (24.4)	43 (29.9)		32 (29.6)	33 (24.1)		45 (29.8)	21 (26.9)		62 (27.9)
		16 (19.5)	22 (15.3)	5.26E-01	19 (17.6)	22 (16.1)	8.83E-01	29 (19.2)	8 (10.3)	1.20E-01	36 (16.2)
NHL case		29 (35.4)	44 (30.6)	5.51E-01	33 (30.6)	46 (33.6)	7.15E-01	55 (36.4)	21 (26.9)	1.94E-01	73 (32.9)
Center*											
	South	4 (4.9)	17 (11.8)	3.45E-02	16 (14.8)	16 (11.7)	2.24E-02	11 (7.3)	9 (11.5)	1.93E-01	19 (8.6)
	Central	47 (57.3)	59 (41.0)		58 (53.7)	54 (39.4)		78 (51.7)	31 (39.7)		105 (47.3)
	North	31 (37.8)	68 (47.2)		34 (31.5)	67 (48.9)		62 (41.1)	38 (48.7)		98 (44.1)
Phase											
	Phase 1	62 (75.6)	106 (73.6)	8.63E-01	87 (80.6)	98 (71.5)	1.39E-01	119 (78.8)	51 (65.4)	4.12E-02	165 (74.3)
	Phase 2	20 (24.4)	38 (26.4)		21 (19.4)	39 (28.5)		32 (21.2)	27 (36.4)		57 (25.7)
Body mass index		25.3 (3.3)	26.0 (3.6)	1.49E-01	24.9 (3.1)	26.5 (3.6)	1.57E-04	25.4 (3.3)	26.5 (3.6)	2.94E-02	25.8 (3.4)
Smoking status											
	Never	36 (43.9)	78 (54.5)	2.86E-01	42 (38.9)	78 (56.9)	1.90E-02	68 (45.0)	45 (57.7)	1.58E-01	112 (50.5)
	Former	25 (30.5)	33 (23.1)		35 (32.4)	30 (21.9)		43 (28.5)	16 (20.5)		58 (26.1)
	Current	21 (25.6)	32 (22.4)		31 (28.7)	29 (21.2)		40 (26.5)	16 (20.5)		52 (23.4)
	Missing	0 (0.0)	1 (0.7)		0 (0.0)	0 (0.0)		0 (0.0)	1 (1.3)		0 (0)
Grams alcohol/day		14.5 (20.2)	10.6 (14.5)	1.28E-01	12.6 (15.9)	11.1 (17)	4.79E-01	12.2 (15.4)	12.6 (19.4)	8.90E-01	12.1 (16.9)

Population features are also summarized for each SEP category.

Counts and percentages are reported for categorical variable, and means and standard deviations for continous variables.

P-value for difference was calculated using the chi-squared test for categorical variables and the Student t-test for continuous variables.

^*^North: Turin & Varese; Central: Florence; South: Naples & Ragusa.

**Table 2:** Linear regression results for the inflammatory transcriptome and each of the three SEP factors in the EPIC-Italy participants from EGM (N = 222).

	Father’s occupational position		Participant’s education		Highest household occupationnal position	
	*β* (SE)	P-value	*β* (SE)	P-value	*β* (SE)	P-value
Inflammatory transcriptome score	21.81 (10.32)	0.036	−0.64 (10.51)	0.951	7.51 (10.78)	0.487
Principal component 1	−4.03 (2.14)	0.061	−0.91 (2.17)	0.676	−2.36 (2.23)	0.289
Cumulative gene ranking-based score*	13.76 (6.14)	0.026	4.64 (3.41)	0.176	10.57 (5.61)	0.061

Sensitivity analysis results are also presented for the principal component 1 and the cumulative gene ranking-based score. ^*^Score is calculated separately for each SEP indicator. Model adjusted on age, gender, lymphoma and breast cancer case-control status, phase and center.

**Table 3:** Life course multiple regression analyses for father’s occupational position and the inflammatory transcriptome.

Variables	Levels	Model A		Model B		Model C		Model D		Fully Adjusted Model*	
		*β* (SE)	P-value	*β* (SE)	P-value	*β* (SE)	P-value	*β* (SE)	P-value	*β* (SE)	P-value
(A) Inflammatory transcriptome score											
Father’s occupational position	Manual	21.81 (10.32)	0.036	26.25 (11.26)	0.021	25.41 (11.39)	0.027	25.37 (11.46)	0.028	23.59 (11.40)	0.040
Participant’s education	Low			−11.21 (11.35)	0.324	−14.09 (12.61)	0.265	−9.81 (12.67)	0.440	−9.07 (12.57)	0.472
Highest household occupational position	Manual					6.59 (12.49)	0.599	9.14 (12.5)	0.465	10.24 (12.40)	0.410
BMI								−3.23 (1.53)	0.036	−3.30 (1.52)	0.031
Smoking status	Former							19.23 (13.44)	0.154	18.78 (13.32)	0.160
	Current							10.19 (13.33)	0.445	11.44 (13.30)	0.391
Alcohol								−0.06 (0.34)	0.868	0.06 (0.34)	0.855
(B) Principal components 1											
Father’s occupational position	Manual	−4.03 (2.14)	0.061	−4.36 (2.34)	0.063	−4.12 (2.36)	0.083	−3.93 (2.39)	0.102	−2.52 (2.05)	0.219
Participant’s education	Low			0.85 (2.35)	0.720	1.68 (2.62)	0.522	1.42 (2.64)	0.591	0.73 (2.26)	0.746
Highest household occupational position	Manual					−1.9 (2.59)	0.464	−1.98 (2.61)	0.449	−2.67 (2.23)	0.232
BMI								0.14 (0.32)	0.657	0.22 (0.27)	0.414
Smoking status	Former							−0.61 (2.8)	0.829	−0.44 (2.39)	0.854
	Current							4.51 (2.78)	0.106	3.10 (2.39)	0.196
Alcohol								0.04 (0.07)	0.535	−0.01 (0.06)	0.852
(C) Cumulative gene ranking-based score											
Father’s occupational position	Manual	13.76 (6.14)	0.026	15.6 (6.71)	0.021	14.85 (6.78)	0.030	14.04 (6.88)	0.043	10.41 (6.12)	0.091
Participant’s education	Low			−4.63 (6.76)	0.494	−7.2 (7.5)	0.338	−5.96 (7.61)	0.434	−4.16 (6.75)	0.538
Highest household occupational position	Manual					5.89 (7.43)	0.429	6.63 (7.5)	0.378	8.39 (6.66)	0.209
BMI								−0.95 (0.92)	0.304	−1.16 (0.82)	0.156
Smoking status	Former							2.78 (8.07)	0.731	2.38 (7.16)	0.740
	Current							−5.71 (8)	0.476	−2.00 (7.16)	0.780
Alcohol								−0.16 (0.2)	0.435	−0.02 (0.18)	0.904

Results are presented for the inflammatory transcriptome score (A), the first PC (B) and the cumulative gene ranking-based score (C). Estimates are based on 222 participants with full SEP and lifestyle information. ^*^Model adjusted for cell blood composition (see Methods).

**Table 4:** Linear regression results for the inflammatory transcriptome and the early-life SEP in participants from the GSE15180 dataset.

	SES in early-life			
	Model non adjusted		Adjusted for cell blood count	
	*β* (SE)	P-value	*β* (SE)	P-value
Inflammatory transcriptome score	24.5 (10.21)	0.020	20.21 (9.59)	0.040
Principal component 1	−2.80 (2.86)	0.332	−1.23 (2.49)	0.624
Cumulative gene ranking-based score*	4.48 (1.11)	0.0002	3.68 (0.78)	0.00002

Sensitivity analysis results are also presented for the principal component 1 and the cumulative gene ranking-based score.

**Table 5:** Linear regression results for each sub-pathway score (model A and D) and father’s occupational position.

Subpathway	Number of genes	*β*	Father’s occupational position				
			Model A		*β*	Model D	
			SE	P-value		SE	P-value
Cytokine signaling	119	3.07	1.46	0.036	3.32	1.61	0.04
MAPK signaling	111	3.62	1.76	0.041	4.89	1.95	0.01
Adhesion-Extravasation-Migration	110	1.53	1.52	0.315	2.29	1.69	0.18
Leukocyte signaling	103	3.62	1.40	0.010	4.02	1.56	0.01
Apoptosis Signaling	64	1.50	1.04	0.149	1.65	1.16	0.16
Phagocytosis-Ag presentation	37	2.13	0.77	0.006	1.82	0.86	0.03
G-Protein Coupled Receptor Signaling	34	0.44	0.53	0.413	0.52	0.59	0.38
Innate pathogen detection	34	0.86	0.75	0.251	0.73	0.84	0.39
PI3K/AKT Signaling	34	0.56	0.60	0.357	0.76	0.67	0.26
Eicosanoid Signaling	32	0.06	0.50	0.898	0.09	0.55	0.88
NF-kB signaling	32	0.59	0.52	0.260	0.77	0.58	0.18
TNF Superfamily Signaling	31	0.78	0.45	0.084	1.01	0.49	0.04
Natural Killer Cell Signaling	29	0.99	0.71	0.161	1.05	0.79	0.18
Complement Cascase	27	0.42	0.48	0.384	0.40	0.54	0.45
ROS/Glutathione/Cytotoxic granules	18	0.68	0.38	0.076	0.76	0.42	0.07
Glucocorticoid/PPAR signaling	17	0.46	0.39	0.245	0.52	0.44	0.23
Calcium Signaling	13	0.51	0.32	0.106	0.78	0.35	0.03
