# Supplementary material for: Biological marks of early-life socioeconomic experience is detected in the adult inflammatory transcriptome
Source: Sci Rep. 2016 Dec 9;6:38705. doi: 10.1038/srep38705 (PMC5146729; doi:10.1038/srep38705)
Supplement: Supplementary Materials [file srep38705-s1.pdf]

# Biological marks of early-life socioeconomic experience is detected in the adult inflammatory transcriptome

**Raphaële Castagné[1,2,3], Michelle Kelly-Irving[2,3], Gianluca Campanella[1], Florence Guida[1], Vittorio Krogh[4], Domenico Palli[5], Salvatore Panico[6], Carlotta Sacerdote[7], Rosario Tumino[8], Jos Kleinjans[9], Theo de Kok[9], Soterios Kyrtopoulos[10], Thierry Lang[2,3], Silvia Stringhini[11], Roel Vermeulen[1,12], Paolo Vineis[1,13,14], Cyrille Delpierre[2,3, a], and Marc Chadeau-Hyam[1,14, a, *]**

[1]Department of Epidemiology and Biostatistics, School of Public Health, Imperial College London, Norfolk Place, W2 1PG London, UK
[2]INSERM, UMR1027, Toulouse F-31000, France
[3]Université Toulouse III Paul-Sabatier, UMR1027, Toulouse F-31000, France
[4]Epidemiology and Prevention Unit, Fondazione IRCCS- Istituto Nazionale dei Tumori, Via Venezian 1, 20133 Milan, Italy
[5]Molecular and Nutritional Epidemiology Unit, Istituto per lo Studio e la Prevenzione Oncologica (ISPO Toscana), Via delle Oblate 2, 50141, Florence, Italy
[6]Department of Clinical Medicine and Surgery, University of Naples Federico II, Via Pansini 5, 80131 Naples, Italy
[7]Piedmont Reference Centre for Epidemiology and Cancer Prevention (CPO Piemonte), Viale Settimio Severo nr. 65, 10133 Turin, Italy
[8]Cancer registry and Histopathology Unit, Azienda Ospedaliera 'Civile –M.P.Arezzo', Via Dante N 109, 97100 Ragusa, Italy
[9]Department of Toxicogenomics, Maastricht University, 6211 LK Maastricht, The Netherlands
[10]National Hellenic Research Foundation, Institute of Biology, Pharmaceutical Chemistry and Biotechnology, Vas. Constantinou 48, 11635 Athens, Greece
[11]Institute of Social and Preventive Medicine, Lausanne University Hospital, Route de la Corniche 10, 1010 Lausanne, Switzerland
[12]Institute for Risk Assessment Sciences (IRAS), Utrecht University, PO Box 80178, 3508 TD, Utrecht, The Netherlands.
[13]HuGeF, Human Genetics Foundation, Via Nizza 52, 10126 Torino, Italy
[14]MRC-PHE Centre for Environment and Health, Imperial College, Praed Street Wing, St Mary's Campus, W2 1PG London, UK
[a]C.D. and M.C-H. contributed equally to this work.
[*]To whom correspondence should be addressed. E-mail: m.chadeau@imperial.ac.uk

## Supplementary Information

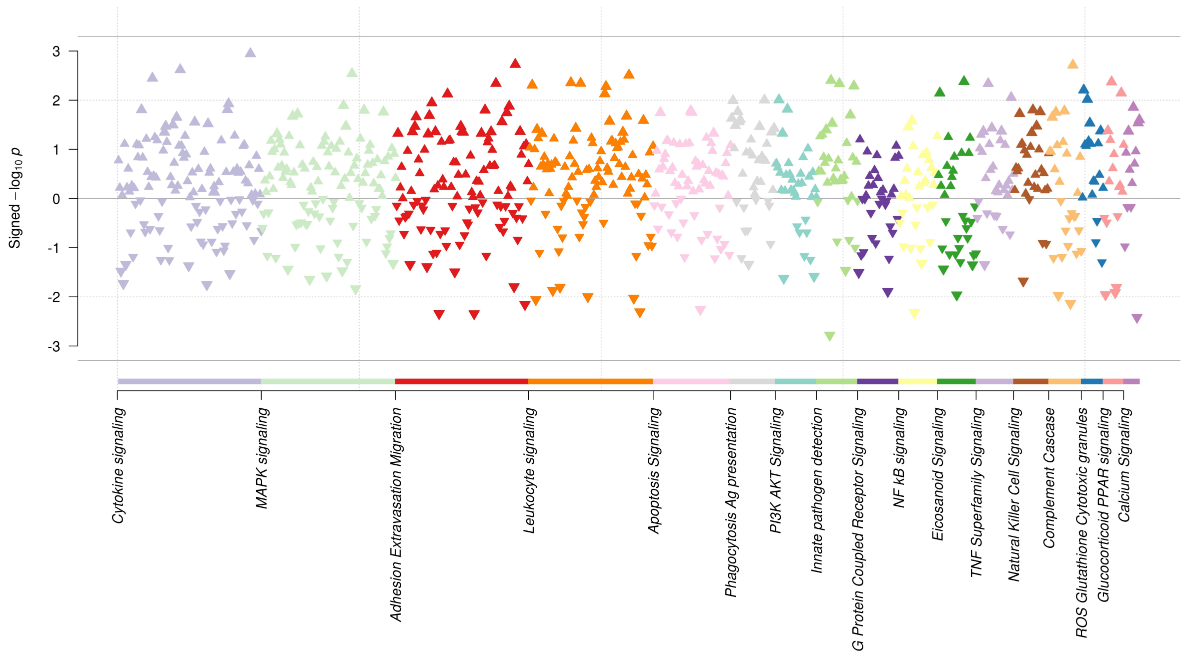


**Fig. S 1.** Inflammatory transcriptome profiling of father's occupational position (N=226). The -log10 p-value is signed by the direction of the effect size estimate and is plotted against each of the 845 genes by sub-pathway. The grey line indicates the per-test significance level controlling the FWER at a 5% level. Results are given for model 1 (see Methods).

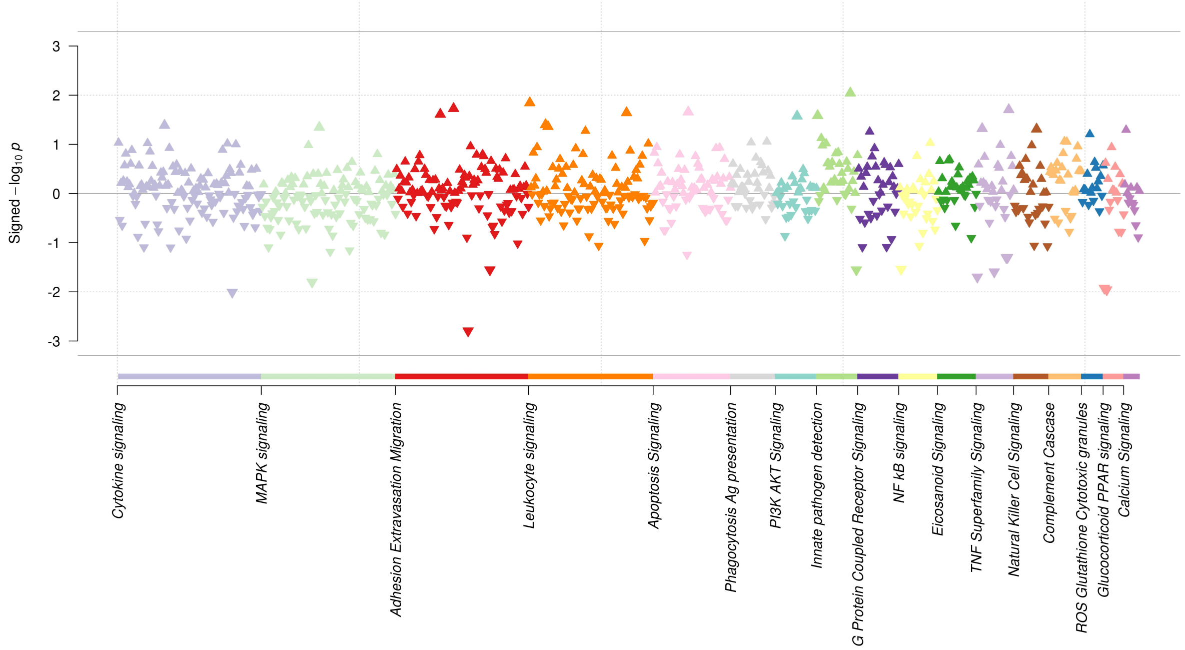


**Fig. S 2.** Inflammatory transcriptome profiling of educational level (N=245). The -log10 p-value is signed by the direction of the effect size estimate and is plotted against each of the 845 genes by sub-pathway. The grey line indicates the per-test significance level controlling the FWER at a 5% level. Results are given for model 1 (see Methods).

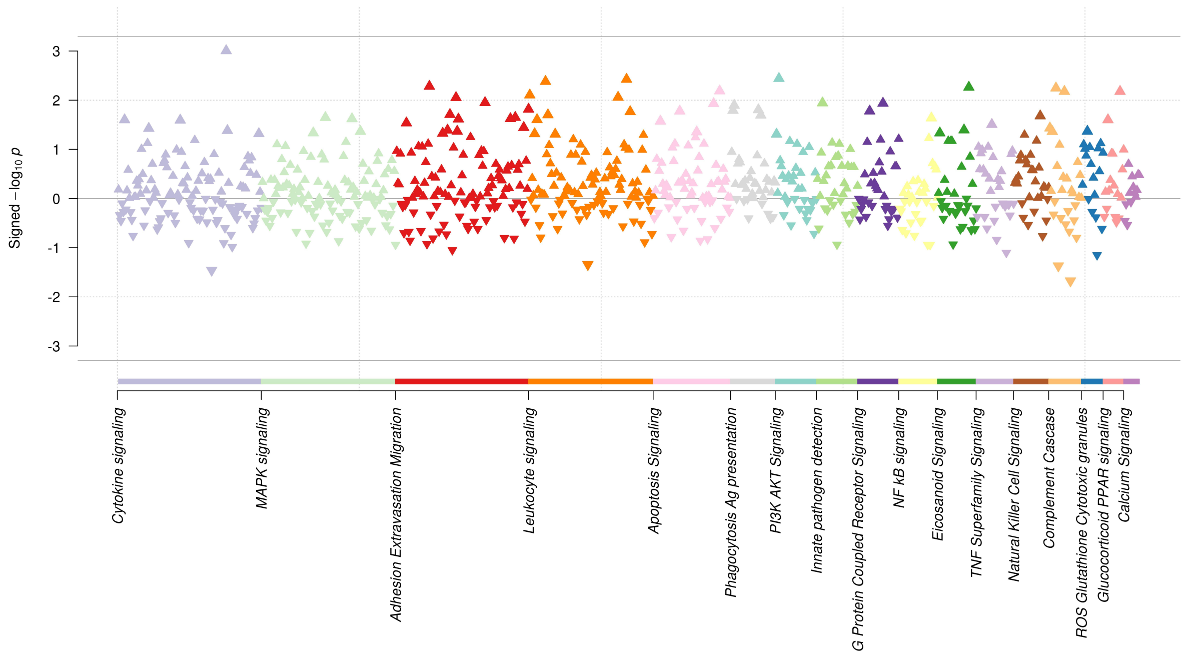


**Fig. S 3.** Inflammatory transcriptome profiling of highest household occupational position (N=229). The -log10 p-value is signed by the direction of the effect size estimate and is plotted against each of the 845 genes by sub-pathway. The grey line indicates the per-test significance level controlling the FWER at a 5% level. Results are given for model 1 (see Methods).

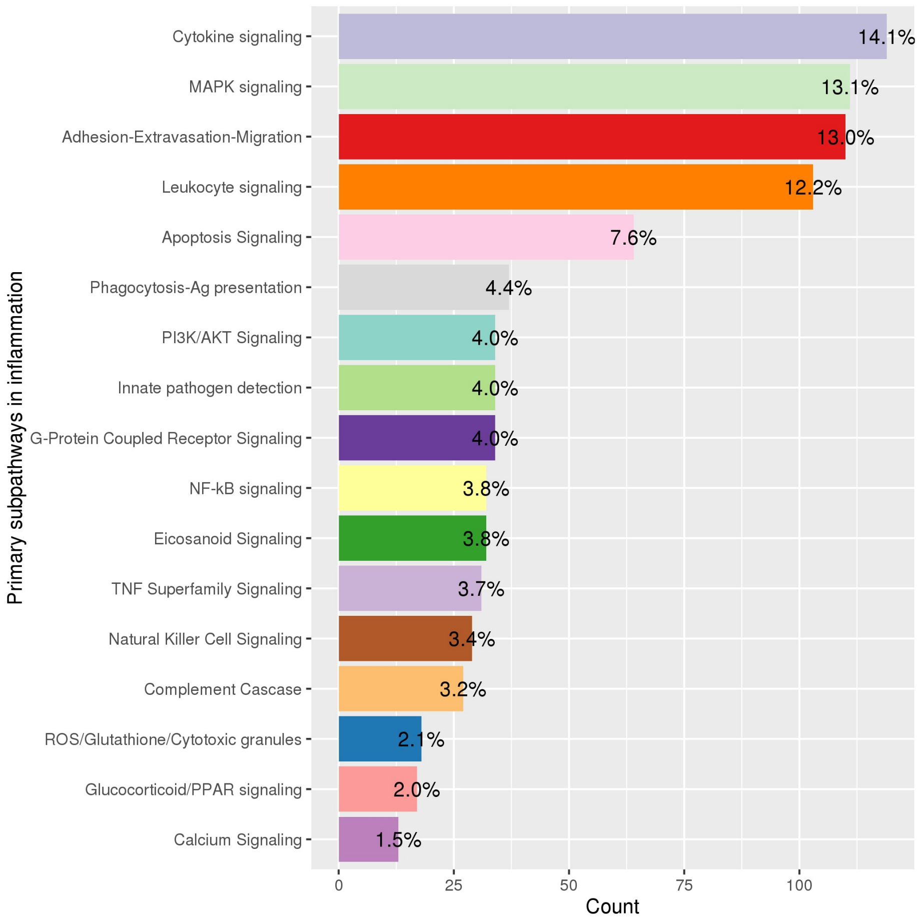


**Fig. S 4.** Bar plot of the number of unique genes on the x-axis by sub-pathways in inflammation on the y-axis. Percentage of genes within each sub-pathways is also reported in bold.

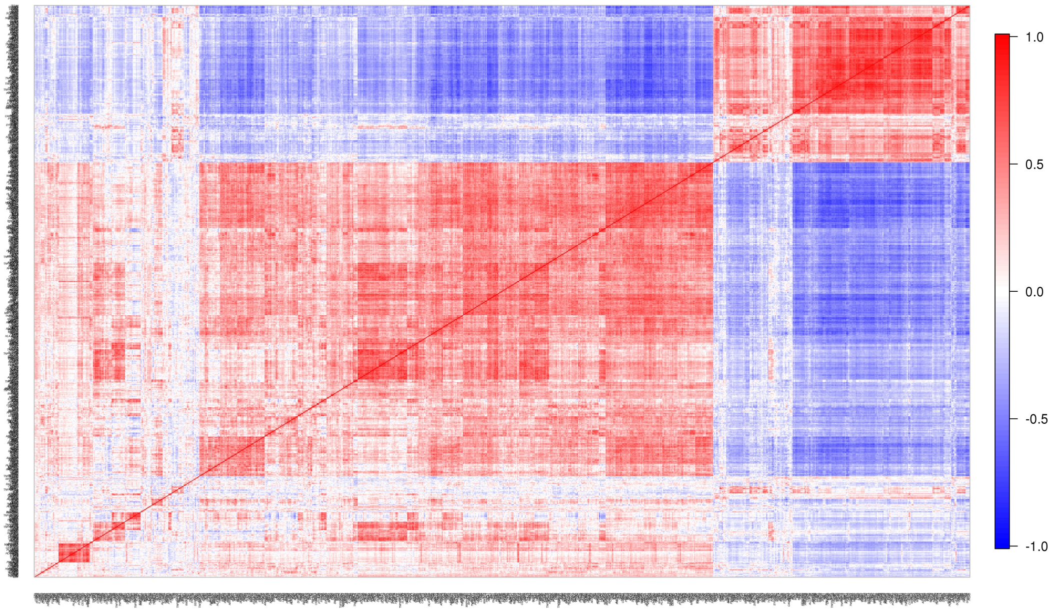


**Fig. S 5.** Gene expression level correlation matrix of EPIC-Italy participants in EnviroGenoMarkers (N=246) with pairwise Spearman's rank correlation.

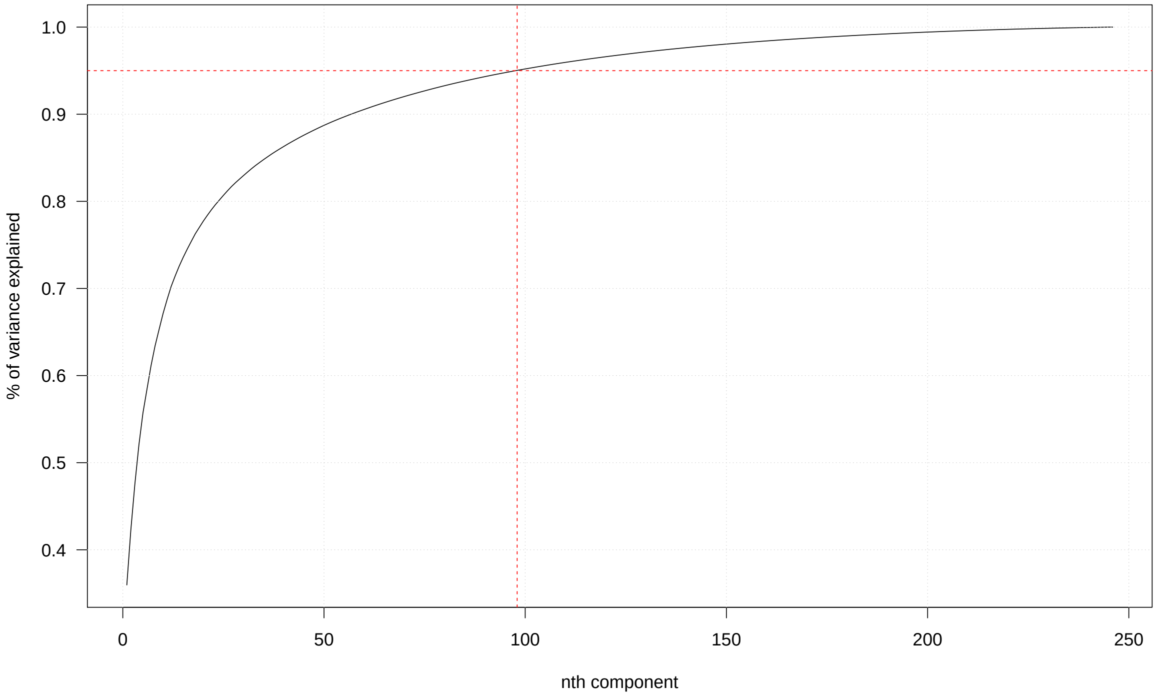


**Fig. S 6.** Scree plot from the PCA of the 845 gene expression levels.

**Table S 1.** Results for the inflammatory transcriptome profiling (N=845) and each of the three SEP factors. Fold-change (f) is derived from the regression coefficient estimate ($\beta$) by the mixed model: f = $2^{\beta}$.

| **Primary Subpathway** | **Symbol** | **AgilentID** | **EntrezID** | **Father's occ. pos. (N=226)** | | **Edu. level (N=245)** | | **High. hous. occ. (N=229)** | |
|---|---|---|---|---|---|---|---|---|---|
| | | | | f | P-value | f | P-value | f | P-value |
| Cytokine signaling | *STAT5B* | A_23_P100788 | 6777 | 1.05 | 1.68E-01 | 1.06 | 9.27E-02 | 1.02 | 6.58E-01 |
| Cytokine signaling | *STAT3* | A_24_P923962 | 6774 | 1.03 | 6.05E-01 | 0.94 | 2.91E-01 | 0.96 | 4.60E-01 |
| Cytokine signaling | *IL12RB1* | A_23_P101783 | 3594 | 0.85 | 3.34E-02 | 1.04 | 6.15E-01 | 0.93 | 3.50E-01 |
| Cytokine signaling | *IL18* | A_23_P104798 | 3606 | 1.01 | 8.58E-01 | 0.93 | 2.23E-01 | 0.99 | 7.99E-01 |
| Cytokine signaling | *IL5RA* | A_23_P109843 | 3568 | 0.79 | 1.86E-02 | 1.06 | 5.50E-01 | 0.94 | 5.34E-01 |
| Cytokine signaling | *CSF1R* | A_23_P110791 | 1436 | 1.12 | 7.75E-02 | 1.08 | 2.69E-01 | 1.16 | 2.51E-02 |
| Cytokine signaling | *EBI3* | A_24_P370201 | 10148 | 0.87 | 4.54E-02 | 1.11 | 1.55E-01 | 0.96 | 5.99E-01 |
| Cytokine signaling | *CSF2RB* | A_23_P120899 | 1439 | 1.02 | 5.95E-01 | 1.02 | 5.75E-01 | 1.02 | 6.88E-01 |
| Cytokine signaling | *JAK2* | A_23_P123608 | 3717 | 1.05 | 2.47E-01 | 1.02 | 7.47E-01 | 1.05 | 2.62E-01 |
| Cytokine signaling | *TGFBR1* | A_23_P123943 | 7046 | 1.01 | 7.23E-01 | 1.02 | 6.52E-01 | 1.00 | 9.79E-01 |
| Cytokine signaling | *IRF2* | A_23_P125082 | 3660 | 0.95 | 2.05E-01 | 1.05 | 2.49E-01 | 0.97 | 5.29E-01 |
| Cytokine signaling | *CSF3R* | A_23_P126218 | 1441 | 1.05 | 5.43E-01 | 1.12 | 1.31E-01 | 0.93 | 3.68E-01 |
| Cytokine signaling | *IL10* | A_23_P126735 | 3586 | 0.89 | 6.47E-02 | 0.99 | 8.99E-01 | 0.92 | 1.74E-01 |
| Cytokine signaling | *IL2RA* | A_23_P127288 | 3559 | 1.04 | 6.20E-01 | 1.00 | 9.91E-01 | 1.01 | 9.36E-01 |
| Cytokine signaling | *SOCS2* | A_24_P230675 | 8835 | 0.98 | 7.84E-01 | 0.97 | 6.11E-01 | 1.01 | 9.27E-01 |
| Cytokine signaling | *IL4R* | A_23_P129556 | 3566 | 1.11 | 7.94E-02 | 0.99 | 8.61E-01 | 1.05 | 4.37E-01 |
| Cytokine signaling | *PIAS2* | A_24_P382266 | 9063 | 1.07 | 1.46E-01 | 0.93 | 1.32E-01 | 1.03 | 5.46E-01 |
| Cytokine signaling | *IL13RA1* | A_24_P280113 | 3597 | 1.11 | 8.32E-02 | 1.08 | 2.70E-01 | 1.06 | 4.08E-01 |
| Cytokine signaling | *IL15RA* | A_23_P138680 | 3601 | 1.00 | 9.20E-01 | 1.01 | 7.11E-01 | 1.01 | 8.07E-01 |
| Cytokine signaling | *TBX21* | A_23_P141555 | 30009 | 1.19 | 1.57E-02 | 1.03 | 6.63E-01 | 1.13 | 9.84E-02 |
| Cytokine signaling | *TYK2* | A_23_P141917 | 7297 | 1.08 | 1.48E-01 | 1.01 | 8.38E-01 | 1.06 | 3.19E-01 |
| Cytokine signaling | *BMPR1A* | A_23_P1431 | 657 | 0.92 | 3.00E-01 | 0.87 | 8.06E-02 | 0.91 | 2.82E-01 |
| Cytokine signaling | *ADAM17* | A_23_P143120 | 6868 | 0.93 | 2.10E-01 | 0.93 | 2.43E-01 | 0.97 | 5.77E-01 |
| Cytokine signaling | *CEBPB* | A_23_P411296 | 1051 | 1.13 | 6.12E-02 | 1.02 | 7.20E-01 | 1.03 | 6.87E-01 |
| Cytokine signaling | *CISH* | A_23_P144096 | 1154 | 1.13 | 2.89E-01 | 1.21 | 9.48E-02 | 1.23 | 7.49E-02 |
| Cytokine signaling | *IL2RG* | A_23_P148473 | 3561 | 1.02 | 5.86E-01 | 1.01 | 7.38E-01 | 1.08 | 3.76E-02 |
| Cytokine signaling | *PIAS3* | A_24_P6889 | 10401 | 0.94 | 2.39E-01 | 1.08 | 1.40E-01 | 0.99 | 8.92E-01 |
| Cytokine signaling | *IFNG* | A_23_P151294 | 3458 | 1.07 | 3.67E-01 | 1.05 | 5.58E-01 | 1.07 | 4.27E-01 |
| Cytokine signaling | *IL32* | A_23_P15146 | 9235 | 1.17 | 3.57E-03 | 1.06 | 2.77E-01 | 1.07 | 1.98E-01 |
| Cytokine signaling | *SOCS4* | A_23_P151700 | 122809 | 1.04 | 3.46E-01 | 1.01 | 7.67E-01 | 1.00 | 9.65E-01 |
| Cytokine signaling | *NMI* | A_23_P154235 | 9111 | 1.10 | 5.68E-02 | 1.02 | 6.68E-01 | 1.02 | 6.55E-01 |
| Cytokine signaling | *IRF4* | A_23_P214360 | 3662 | 1.06 | 3.76E-01 | 0.99 | 9.11E-01 | 1.02 | 7.64E-01 |

| | | | | | | | | | |
|---|---|---|---|---|---|---|---|---|---|
| Cytokine signaling | *PIAS1* | A_23_P163347 | 8554 | 1.08 | 6.57E-02 | 0.95 | 2.35E-01 | 0.95 | 2.36E-01 |
| Cytokine signaling | *OSM* | A_23_P166408 | 5008 | 0.92 | 4.39E-01 | 0.87 | 1.71E-01 | 0.88 | 2.60E-01 |
| Cytokine signaling | *IL17B* | A_23_P167479 | 27190 | 0.90 | 2.26E-01 | 1.04 | 6.95E-01 | 0.92 | 3.46E-01 |
| Cytokine signaling | *IL17F* | A_23_P167882 | 112744 | 0.84 | 5.61E-02 | 1.09 | 3.76E-01 | 0.92 | 3.97E-01 |
| Cytokine signaling | *IL1RAP* | A_23_P336554 | 3556 | 0.98 | 8.74E-01 | 1.07 | 4.75E-01 | 0.98 | 8.07E-01 |
| Cytokine signaling | *IFNAR1* | A_23_P17633 | 3454 | 1.07 | 1.06E-01 | 1.04 | 3.63E-01 | 1.06 | 1.92E-01 |
| Cytokine signaling | *IL17RA* | A_32_P103815 | 23765 | 1.13 | 4.48E-02 | 1.13 | 4.12E-02 | 1.09 | 1.67E-01 |
| Cytokine signaling | *CASP1* | A_23_P202978 | 834 | 1.12 | 3.39E-02 | 1.02 | 7.01E-01 | 1.09 | 1.28E-01 |
| Cytokine signaling | *IL10RA* | A_23_P203173 | 3587 | 1.10 | 7.10E-02 | 0.96 | 5.07E-01 | 1.07 | 2.33E-01 |
| Cytokine signaling | *SOCS3* | A_23_P351069 | 9021 | 0.85 | 9.96E-02 | 1.04 | 6.59E-01 | 0.93 | 5.11E-01 |
| Cytokine signaling | *STAT5A* | A_24_P173088 | 6776 | 0.85 | 5.56E-02 | 1.14 | 1.19E-01 | 1.02 | 8.62E-01 |
| Cytokine signaling | *PTPN2* | A_23_P309701 | 5771 | 1.05 | 3.66E-01 | 0.91 | 8.02E-02 | 1.02 | 7.67E-01 |
| Cytokine signaling | *IL1RN* | A_23_P209995 | 3557 | 1.06 | 5.39E-01 | 0.96 | 6.69E-01 | 1.02 | 8.49E-01 |
| Cytokine signaling | *IFNAR2* | A_23_P211080 | 3455 | 1.15 | 3.19E-02 | 1.07 | 2.83E-01 | 1.06 | 3.47E-01 |
| Cytokine signaling | *TGFBR2* | A_23_P211957 | 7048 | 1.06 | 1.88E-01 | 0.97 | 4.87E-01 | 0.97 | 4.56E-01 |
| Cytokine signaling | *IL4* | A_23_P213706 | 3565 | 1.04 | 6.91E-01 | 1.09 | 3.67E-01 | 0.90 | 3.20E-01 |
| Cytokine signaling | *S100A9* | A_23_P23048 | 6280 | 1.19 | 2.23E-02 | 1.09 | 2.58E-01 | 1.14 | 1.01E-01 |
| Cytokine signaling | *IL3RA* | A_32_P217750 | 3563 | 1.05 | 5.54E-01 | 1.00 | 9.72E-01 | 1.02 | 8.22E-01 |
| Cytokine signaling | *ILF2* | A_23_P257956 | 3608 | 1.05 | 8.45E-02 | 0.96 | 1.77E-01 | 1.00 | 9.51E-01 |
| Cytokine signaling | *IL27RA* | A_24_P348326 | 9466 | 1.16 | 2.40E-03 | 1.05 | 3.54E-01 | 1.12 | 2.55E-02 |
| Cytokine signaling | *IRF3* | A_23_P27677 | 3661 | 1.07 | 8.41E-02 | 1.01 | 8.51E-01 | 1.06 | 1.13E-01 |
| Cytokine signaling | *IL18RAP* | A_23_P28334 | 8807 | 1.11 | 1.20E-01 | 1.01 | 9.13E-01 | 1.05 | 4.85E-01 |
| Cytokine signaling | *IFNGR2* | A_23_P29036 | 3460 | 1.08 | 1.48E-01 | 0.99 | 8.84E-01 | 1.04 | 4.95E-01 |
| Cytokine signaling | *IL15* | A_23_P29953 | 3600 | 1.11 | 5.32E-02 | 1.05 | 3.86E-01 | 1.07 | 2.21E-01 |
| Cytokine signaling | *STAT4* | A_23_P305198 | 6775 | 1.12 | 1.27E-01 | 0.89 | 1.21E-01 | 1.03 | 6.68E-01 |
| Cytokine signaling | *NFIL3* | A_23_P32253 | 4783 | 1.06 | 4.47E-01 | 0.95 | 5.14E-01 | 1.08 | 3.75E-01 |
| Cytokine signaling | *JAK3* | A_24_P308096 | 3718 | 0.86 | 4.29E-02 | 1.04 | 6.00E-01 | 0.89 | 1.24E-01 |
| Cytokine signaling | *IRF8* | A_23_P332190 | 3394 | 1.00 | 9.49E-01 | 0.93 | 2.83E-01 | 0.99 | 8.40E-01 |
| Cytokine signaling | *IL17A* | A_23_P332820 | 3605 | 0.90 | 2.72E-01 | 1.10 | 3.11E-01 | 0.96 | 6.47E-01 |
| Cytokine signaling | *IL7R* | A_23_P33643 | 3575 | 1.07 | 2.96E-01 | 0.98 | 7.40E-01 | 1.01 | 8.91E-01 |
| Cytokine signaling | *IL29* | A_23_P337800 | 282618 | 1.02 | 7.53E-01 | 1.01 | 8.82E-01 | 0.97 | 5.73E-01 |
| Cytokine signaling | *PTPN1* | A_23_P338890 | 5770 | 1.10 | 2.79E-02 | 0.99 | 9.15E-01 | 1.09 | 6.50E-02 |
| Cytokine signaling | *IL1F6* | A_23_P348028 | 27179 | 0.97 | 7.06E-01 | 1.02 | 7.86E-01 | 1.03 | 7.00E-01 |
| Cytokine signaling | *IL19* | A_23_P35092 | 29949 | 0.93 | 3.33E-01 | 0.96 | 6.26E-01 | 0.93 | 3.89E-01 |
| Cytokine signaling | *ACE* | A_24_P365129 | 1636 | 1.14 | 2.52E-01 | 1.01 | 9.38E-01 | 1.10 | 4.38E-01 |
| Cytokine signaling | *CSF1* | A_23_P407012 | 1435 | 0.97 | 5.42E-01 | 1.02 | 6.94E-01 | 0.95 | 3.77E-01 |
| Cytokine signaling | *IL28A* | A_23_P409438 | 282616 | 0.89 | 1.34E-01 | 1.06 | 4.97E-01 | 0.95 | 5.75E-01 |
| Cytokine signaling | *IRF1* | A_23_P41765 | 3659 | 1.02 | 6.46E-01 | 0.96 | 4.10E-01 | 1.06 | 3.09E-01 |
| Cytokine signaling | *SOCS1* | A_23_P420196 | 8651 | 0.89 | 1.17E-01 | 1.05 | 5.24E-01 | 0.96 | 5.69E-01 |
| Cytokine signaling | *PDGFRB* | A_23_P421401 | 5159 | 1.18 | 1.13E-01 | 0.93 | 4.98E-01 | 1.15 | 1.83E-01 |
| Cytokine signaling | *IL31RA* | A_23_P423348 | 133396 | 0.97 | 6.04E-01 | 1.02 | 7.56E-01 | 1.00 | 9.90E-01 |
| Cytokine signaling | *IL23R* | A_23_P425197 | 149233 | 0.81 | 1.77E-02 | 0.92 | 3.38E-01 | 0.91 | 3.29E-01 |
| Cytokine signaling | *S100A8* | A_23_P434809 | 6279 | 1.19 | 3.02E-02 | 1.07 | 3.89E-01 | 1.07 | 3.86E-01 |
| Cytokine signaling | *STAT6* | A_23_P47879 | 6778 | 1.06 | 2.99E-01 | 0.99 | 9.19E-01 | 1.05 | 4.46E-01 |
| Cytokine signaling | *IRF5* | A_24_P363609 | 3663 | 1.14 | 2.46E-01 | 0.97 | 7.62E-01 | 1.10 | 3.97E-01 |
| Cytokine signaling | *IL1F10* | A_23_P501713 | 84639 | 0.90 | 1.33E-01 | 0.96 | 5.85E-01 | 0.85 | 3.48E-02 |
| Cytokine signaling | *IL6ST* | A_24_P935033 | 3572 | 1.03 | 7.59E-01 | 0.91 | 2.68E-01 | 0.95 | 5.95E-01 |
| Cytokine signaling | *TXLNA* | A_23_P51361 | 200081 | 1.04 | 5.67E-01 | 1.03 | 6.54E-01 | 1.11 | 1.26E-01 |
| Cytokine signaling | *IL24* | A_23_P51951 | 11009 | 1.02 | 7.61E-01 | 0.98 | 6.91E-01 | 1.00 | 9.71E-01 |
| Cytokine signaling | *IL1F7* | A_23_P5654 | 27178 | 0.88 | 8.27E-02 | 1.03 | 6.33E-01 | 0.93 | 3.21E-01 |
| Cytokine signaling | *IL1RL2* | A_23_P56604 | 8808 | 0.95 | 2.41E-01 | 1.02 | 6.79E-01 | 0.96 | 3.64E-01 |
| Cytokine signaling | *STAT1* | A_23_P56630 | 6772 | 1.04 | 4.69E-01 | 1.00 | 9.94E-01 | 1.05 | 2.99E-01 |
| Cytokine signaling | *SOCS5* | A_23_P5813 | 9655 | 0.99 | 8.93E-01 | 0.99 | 8.53E-01 | 0.99 | 8.58E-01 |
| Cytokine signaling | *IL16* | A_24_P73599 | 3603 | 1.10 | 6.59E-02 | 1.03 | 6.05E-01 | 1.02 | 7.25E-01 |
| Cytokine signaling | *IL1R1* | A_24_P200023 | 3554 | 0.95 | 2.74E-01 | 1.09 | 1.30E-01 | 0.99 | 7.96E-01 |
| Cytokine signaling | *IL6* | A_23_P71037 | 3569 | 0.85 | 8.79E-02 | 0.92 | 4.05E-01 | 0.86 | 1.24E-01 |
| Cytokine signaling | *IFNB1* | A_23_P71774 | 3456 | 0.86 | 1.11E-01 | 1.05 | 6.22E-01 | 0.89 | 2.50E-01 |
| Cytokine signaling | *IL12RB2* | A_23_P72077 | 3595 | 1.08 | 2.92E-01 | 1.05 | 4.40E-01 | 1.26 | 9.89E-04 |
| Cytokine signaling | *S100A12* | A_23_P74001 | 6283 | 1.22 | 1.56E-02 | 1.15 | 9.64E-02 | 1.19 | 4.15E-02 |
| Cytokine signaling | *GATA3* | A_23_P75056 | 2625 | 1.15 | 1.19E-02 | 1.00 | 9.66E-01 | 1.04 | 4.82E-01 |
| Cytokine signaling | *STAT2* | A_23_P76090 | 6773 | 0.90 | 2.97E-02 | 1.05 | 3.22E-01 | 0.98 | 6.68E-01 |
| Cytokine signaling | *IL1R2* | A_24_P63019 | 7850 | 0.99 | 9.49E-01 | 1.09 | 3.86E-01 | 0.88 | 1.83E-01 |
| Cytokine signaling | *IL1B* | A_23_P79518 | 3553 | 0.86 | 3.13E-01 | 0.67 | 9.86E-03 | 0.78 | 1.03E-01 |
| Cytokine signaling | *TGIF2* | A_23_P79794 | 60436 | 1.05 | 2.85E-01 | 0.96 | 3.26E-01 | 0.97 | 5.54E-01 |
| Cytokine signaling | *IFNGR1* | A_23_P8281 | 3459 | 1.10 | 5.27E-02 | 1.00 | 9.83E-01 | 0.96 | 4.57E-01 |
| Cytokine signaling | *ENG* | A_23_P83328 | 2022 | 0.95 | 4.89E-01 | 1.12 | 1.00E-01 | 0.96 | 5.93E-01 |
| Cytokine signaling | *TGFB3* | A_23_P88404 | 7043 | 1.06 | 2.46E-01 | 0.96 | 4.35E-01 | 0.96 | 3.88E-01 |
| Cytokine signaling | *IL7* | A_23_P8961 | 3574 | 1.08 | 3.01E-01 | 0.96 | 5.71E-01 | 0.97 | 6.65E-01 |
| Cytokine signaling | *PIAS4* | A_23_P90280 | 51588 | 1.00 | 9.67E-01 | 1.05 | 3.77E-01 | 0.98 | 7.13E-01 |
| Cytokine signaling | *AGER* | A_23_P93360 | 177 | 1.05 | 2.86E-01 | 1.05 | 2.62E-01 | 1.03 | 5.66E-01 |
| Cytokine signaling | *BMPR2* | A_24_P753161 | 659 | 0.96 | 5.44E-01 | 0.97 | 6.69E-01 | 0.97 | 6.70E-01 |
| Cytokine signaling | *JAK1* | A_24_P410678 | 3716 | 1.08 | 8.59E-02 | 0.97 | 4.87E-01 | 1.03 | 5.81E-01 |
| Cytokine signaling | *HMGB1* | A_24_P169148 | 3146 | 1.10 | 8.01E-02 | 0.94 | 2.20E-01 | 1.07 | 2.30E-01 |
| Cytokine signaling | *IRF7* | A_24_P378019 | 3665 | 1.05 | 4.32E-01 | 0.99 | 8.14E-01 | 1.03 | 6.27E-01 |
| Cytokine signaling | *LIF* | A_24_P233488 | 3976 | 0.88 | 2.86E-01 | 1.05 | 6.96E-01 | 0.92 | 4.88E-01 |
| Cytokine signaling | *IL2RB* | A_24_P203000 | 3560 | 1.04 | 4.92E-01 | 1.00 | 9.39E-01 | 1.09 | 1.39E-01 |
| Cytokine signaling | *IL18R1* | A_24_P208567 | 8809 | 1.07 | 2.12E-01 | 1.02 | 7.18E-01 | 1.08 | 1.69E-01 |
| Cytokine signaling | *IL21R* | A_24_P227927 | 50615 | 1.17 | 1.14E-03 | 0.97 | 5.28E-01 | 1.05 | 3.04E-01 |
| Cytokine signaling | *IL10RB* | A_24_P322741 | 3588 | 1.01 | 8.43E-01 | 1.05 | 3.28E-01 | 1.06 | 1.46E-01 |
| Cytokine signaling | *PDGFB* | A_24_P339944 | 5155 | 0.99 | 9.29E-01 | 0.95 | 3.73E-01 | 0.93 | 2.51E-01 |
| Cytokine signaling | *CRP* | A_24_P342484 | 1401 | 0.87 | 1.43E-01 | 0.93 | 4.26E-01 | 0.90 | 3.11E-01 |
| Cytokine signaling | *THPO* | A_24_P377124 | 7066 | 1.02 | 8.00E-01 | 0.91 | 2.06E-01 | 0.94 | 3.99E-01 |
| Cytokine signaling | *IL6R* | A_24_P379413 | 3570 | 1.08 | 1.36E-01 | 1.06 | 3.15E-01 | 1.05 | 3.31E-01 |

| | | | | | | | | | |
|---|---|---|---|---|---|---|---|---|---|
| Cytokine signaling | *IFNA4* | A_24_P403459 | 3441 | 0.98 | 7.87E-01 | 1.00 | 9.59E-01 | 0.96 | 5.73E-01 |
| Cytokine signaling | *TGFB1* | A_24_P79054 | 7040 | 1.05 | 2.50E-01 | 0.99 | 8.52E-01 | 1.09 | 4.81E-02 |
| Cytokine signaling | *SOCS6* | A_24_P91272 | 9306 | 1.02 | 8.25E-01 | 0.93 | 4.46E-01 | 1.03 | 7.31E-01 |
| Cytokine signaling | *IL8* | A_32_P87013 | 3576 | 0.78 | 2.19E-01 | 0.99 | 9.71E-01 | 0.91 | 6.49E-01 |
| MAPK signaling | *YWHAH* | A_23_P103070 | 7533 | 0.95 | 2.53E-01 | 0.97 | 5.39E-01 | 1.03 | 4.64E-01 |
| MAPK signaling | *PPP2R3A* | A_24_P388433 | 5523 | 0.95 | 4.94E-01 | 1.03 | 6.55E-01 | 1.00 | 9.94E-01 |
| MAPK signaling | *ATF1* | A_23_P105646 | 466 | 1.03 | 4.31E-01 | 0.96 | 3.47E-01 | 0.98 | 5.93E-01 |
| MAPK signaling | *FOS* | A_23_P106194 | 2353 | 1.04 | 7.38E-01 | 0.96 | 6.89E-01 | 0.90 | 3.57E-01 |
| MAPK signaling | *PPP2R1A* | A_23_P107661 | 5518 | 0.87 | 6.80E-02 | 0.91 | 2.03E-01 | 0.95 | 5.43E-01 |
| MAPK signaling | *DUSP2* | A_24_P37409 | 1844 | 0.94 | 5.24E-01 | 0.85 | 8.04E-02 | 0.96 | 6.43E-01 |
| MAPK signaling | *DUSP1* | A_23_P110712 | 1843 | 1.08 | 3.56E-01 | 0.89 | 1.90E-01 | 0.91 | 2.79E-01 |
| MAPK signaling | *RASGRP1* | A_32_P195478 | 10125 | 1.09 | 6.71E-02 | 0.95 | 3.30E-01 | 1.04 | 4.14E-01 |
| MAPK signaling | *HDAC1* | A_23_P114656 | 3065 | 1.05 | 1.13E-01 | 0.98 | 3.97E-01 | 0.98 | 4.04E-01 |
| MAPK signaling | *PLA2G4A* | A_23_P11685 | 5321 | 1.03 | 6.43E-01 | 0.99 | 8.32E-01 | 0.99 | 8.60E-01 |
| MAPK signaling | *SOS2* | A_23_P117546 | 6655 | 1.08 | 9.99E-02 | 0.94 | 4.40E-01 | 1.04 | 4.19E-01 |
| MAPK signaling | *MAP2K3* | A_24_P296698 | 5606 | 0.91 | 6.20E-02 | 1.00 | 9.19E-01 | 1.01 | 8.14E-01 |
| MAPK signaling | *YWHAB* | A_23_P500251 | 7529 | 1.09 | 4.84E-02 | 0.98 | 6.61E-01 | 1.08 | 7.91E-02 |
| MAPK signaling | *ATF4* | A_23_P120933 | 468 | 1.06 | 2.36E-01 | 0.95 | 2.90E-01 | 1.03 | 5.62E-01 |
| MAPK signaling | *PPP2CA* | A_23_P122041 | 5515 | 1.07 | 6.33E-02 | 0.97 | 5.65E-01 | 1.02 | 6.17E-01 |
| MAPK signaling | *HDAC2* | A_23_P122304 | 3066 | 1.07 | 8.01E-02 | 0.95 | 1.46E-01 | 0.97 | 5.24E-01 |
| MAPK signaling | *YWHAG* | A_24_P106681 | 7532 | 1.06 | 2.16E-01 | 1.03 | 6.04E-01 | 1.07 | 1.59E-01 |
| MAPK signaling | *ETS1* | A_23_P127525 | 2113 | 1.05 | 3.85E-01 | 1.02 | 6.41E-01 | 1.03 | 5.98E-01 |
| MAPK signaling | *YWHAQ* | A_24_P199905 | 10971 | 1.08 | 1.17E-01 | 0.99 | 8.11E-01 | 1.05 | 2.93E-01 |
| MAPK signaling | *MAP3K5* | A_23_P134125 | 4217 | 1.04 | 2.85E-01 | 1.00 | 9.94E-01 | 1.08 | 8.60E-02 |
| MAPK signaling | *PPP2CB* | A_23_P134693 | 5516 | 1.06 | 2.49E-01 | 0.97 | 5.53E-01 | 1.04 | 5.08E-01 |
| MAPK signaling | *DUSP4* | A_23_P134935 | 1846 | 0.85 | 3.66E-02 | 1.05 | 5.29E-01 | 0.95 | 5.13E-01 |
| MAPK signaling | *PRKCQ* | A_23_P1374 | 5588 | 1.11 | 5.37E-02 | 0.94 | 2.85E-01 | 1.04 | 5.40E-01 |
| MAPK signaling | *DUSP6* | A_23_P139704 | 1848 | 1.05 | 4.68E-01 | 1.02 | 7.45E-01 | 1.09 | 2.08E-01 |
| MAPK signaling | *PRKCD* | A_23_P144054 | 5580 | 1.02 | 5.28E-01 | 1.02 | 4.62E-01 | 1.00 | 8.81E-01 |
| MAPK signaling | *MAPK13* | A_24_P406132 | 5603 | 1.09 | 2.12E-02 | 1.02 | 6.47E-01 | 1.01 | 8.09E-01 |
| MAPK signaling | *LYN* | A_23_P147431 | 4067 | 1.05 | 2.96E-01 | 0.98 | 7.54E-01 | 1.02 | 6.42E-01 |
| MAPK signaling | *MAX* | A_23_P436138 | 4149 | 0.92 | 1.04E-01 | 1.04 | 4.85E-01 | 1.00 | 9.45E-01 |
| MAPK signaling | *PPM1A* | A_23_P409553 | 5494 | 0.95 | 2.97E-01 | 0.95 | 2.46E-01 | 0.94 | 2.11E-01 |
| MAPK signaling | *PPP1R7* | A_23_P154199 | 5510 | 1.07 | 1.91E-02 | 1.00 | 8.83E-01 | 1.03 | 2.59E-01 |
| MAPK signaling | *EGF* | A_23_P155979 | 1950 | 0.93 | 3.13E-01 | 0.91 | 1.88E-01 | 1.02 | 7.49E-01 |
| MAPK signaling | *IFI16* | A_23_P160025 | 3428 | 1.10 | 8.30E-02 | 1.00 | 9.57E-01 | 1.03 | 5.96E-01 |
| MAPK signaling | *FOSL1* | A_23_P161624 | 8061 | 0.94 | 3.27E-01 | 0.99 | 9.10E-01 | 0.99 | 8.44E-01 |
| MAPK signaling | *MAPK9* | A_23_P167692 | 5601 | 1.05 | 2.05E-01 | 0.99 | 6.98E-01 | 1.00 | 9.91E-01 |
| MAPK signaling | *KCNH8* | A_23_P170636 | 131096 | 0.97 | 7.50E-01 | 1.04 | 6.57E-01 | 1.19 | 1.09E-01 |
| MAPK signaling | *ELK1* | A_23_P171054 | 2002 | 1.07 | 2.47E-01 | 0.96 | 5.37E-01 | 1.05 | 4.20E-01 |
| MAPK signaling | *RASA1* | A_23_P18939 | 5921 | 0.97 | 3.01E-01 | 0.96 | 1.96E-01 | 0.95 | 1.21E-01 |
| MAPK signaling | *INS* | A_23_P1981 | 3630 | 0.95 | 3.03E-01 | 1.04 | 4.49E-01 | 0.98 | 6.44E-01 |
| MAPK signaling | *MKNK1* | A_23_P200126 | 8569 | 0.98 | 5.51E-01 | 1.07 | 1.04E-01 | 1.01 | 8.89E-01 |
| MAPK signaling | *SHC1* | A_24_P68585 | 6464 | 0.82 | 2.28E-02 | 0.99 | 8.52E-01 | 0.89 | 2.01E-01 |
| MAPK signaling | *MAPKAPK2* | A_23_P350467 | 9261 | 1.03 | 6.02E-01 | 0.98 | 7.12E-01 | 1.07 | 1.42E-01 |
| MAPK signaling | *JUN* | A_23_P201538 | 3725 | 1.03 | 7.24E-01 | 0.77 | 1.58E-02 | 0.94 | 5.60E-01 |
| MAPK signaling | *MAP2K1* | A_23_P20248 | 5604 | 1.06 | 1.80E-01 | 1.04 | 3.99E-01 | 1.09 | 4.64E-02 |
| MAPK signaling | *MAPK8* | A_23_P356152 | 5599 | 0.96 | 4.98E-01 | 0.95 | 3.93E-01 | 0.95 | 4.31E-01 |
| MAPK signaling | *MADD* | A_23_P202708 | 8567 | 0.99 | 8.01E-01 | 1.00 | 9.27E-01 | 0.99 | 7.80E-01 |
| MAPK signaling | *PPP1CC* | A_23_P204423 | 5501 | 1.05 | 2.29E-01 | 0.96 | 4.21E-01 | 0.98 | 7.26E-01 |
| MAPK signaling | *MAP2K6* | A_23_P207445 | 5608 | 1.03 | 4.49E-01 | 1.04 | 3.97E-01 | 1.03 | 4.62E-01 |
| MAPK signaling | *KSR1* | A_24_P255483 | 8844 | 0.94 | 4.33E-01 | 1.18 | 4.52E-02 | 1.00 | 9.90E-01 |
| MAPK signaling | *MAP2K2* | A_23_P208835 | 5605 | 0.96 | 9.70E-02 | 1.01 | 6.45E-01 | 0.99 | 7.67E-01 |
| MAPK signaling | *ATF2* | A_23_P209879 | 1386 | 1.09 | 8.47E-02 | 1.00 | 9.99E-01 | 1.08 | 1.27E-01 |
| MAPK signaling | *DDIT3* | A_23_P21134 | 1649 | 1.00 | 9.82E-01 | 0.96 | 3.74E-01 | 0.95 | 2.83E-01 |
| MAPK signaling | *PPP2R2B* | A_23_P213620 | 5521 | 1.10 | 6.98E-02 | 1.02 | 7.00E-01 | 1.08 | 1.62E-01 |
| MAPK signaling | *RAC1* | A_32_P217709 | 5879 | 1.07 | 5.51E-02 | 0.99 | 7.37E-01 | 1.08 | 2.26E-02 |
| MAPK signaling | *MYC* | A_24_P178011 | 4609 | 0.82 | 2.66E-02 | 1.08 | 3.83E-01 | 0.89 | 2.10E-01 |
| MAPK signaling | *RAC2* | A_24_P228717 | 5880 | 1.09 | 1.16E-01 | 0.96 | 4.17E-01 | 1.03 | 5.50E-01 |
| MAPK signaling | *PTK2* | A_23_P22096 | 5747 | 1.09 | 2.24E-01 | 0.92 | 1.93E-01 | 0.93 | 3.25E-01 |
| MAPK signaling | *PRKCE* | A_23_P250564 | 5581 | 0.96 | 3.57E-01 | 0.92 | 6.54E-02 | 0.99 | 7.94E-01 |
| MAPK signaling | *PLCG1* | A_23_P254801 | 5335 | 1.08 | 8.55E-02 | 0.96 | 3.78E-01 | 1.02 | 7.52E-01 |
| MAPK signaling | *HSPB1* | A_24_P86537 | 3315 | 0.98 | 6.48E-01 | 0.95 | 1.40E-01 | 0.98 | 5.71E-01 |
| MAPK signaling | *MAPK1* | A_23_P257895 | 5594 | 1.00 | 8.65E-01 | 1.01 | 8.57E-01 | 1.01 | 8.18E-01 |
| MAPK signaling | *ETS2* | A_24_P314179 | 2114 | 1.08 | 2.95E-01 | 0.96 | 5.97E-01 | 1.03 | 6.65E-01 |
| MAPK signaling | *PPP1CB* | A_24_P396720 | 5500 | 1.07 | 9.74E-02 | 0.96 | 3.36E-01 | 1.01 | 8.49E-01 |
| MAPK signaling | *MAPK12* | A_23_P29347 | 6300 | 0.82 | 1.28E-01 | 1.13 | 3.33E-01 | 1.00 | 9.79E-01 |
| MAPK signaling | *MYCN* | A_24_P94402 | 4613 | 0.86 | 6.52E-02 | 0.98 | 8.23E-01 | 0.88 | 1.36E-01 |
| MAPK signaling | *RAPGEF1* | A_23_P391764 | 2889 | 1.09 | 6.99E-02 | 0.96 | 3.97E-01 | 0.96 | 4.46E-01 |
| MAPK signaling | *KRAS* | A_23_P306507 | 3845 | 1.03 | 5.61E-01 | 1.01 | 8.89E-01 | 0.99 | 8.80E-01 |
| MAPK signaling | *SRC* | A_23_P308603 | 6714 | 0.88 | 3.38E-02 | 1.02 | 6.86E-01 | 0.95 | 3.91E-01 |
| MAPK signaling | *MAPKAPK3* | A_23_P314584 | 7867 | 1.09 | 1.28E-02 | 1.02 | 5.17E-01 | 1.06 | 1.38E-01 |
| MAPK signaling | *MEF2C* | A_23_P320739 | 4208 | 1.05 | 3.95E-01 | 0.98 | 6.88E-01 | 1.05 | 4.20E-01 |
| MAPK signaling | *PPP1CA* | A_23_P323227 | 5499 | 1.08 | 3.39E-02 | 1.00 | 9.86E-01 | 1.07 | 7.07E-02 |
| MAPK signaling | *MEF2D* | A_23_P51679 | 4209 | 0.84 | 5.15E-02 | 1.09 | 2.99E-01 | 0.95 | 5.37E-01 |
| MAPK signaling | *SOS1* | A_23_P343808 | 6654 | 1.08 | 1.16E-01 | 1.01 | 7.48E-01 | 1.04 | 4.72E-01 |
| MAPK signaling | *ATF3* | A_23_P34915 | 467 | 1.00 | 9.75E-01 | 0.82 | 7.00E-02 | 0.93 | 5.19E-01 |
| MAPK signaling | *MEF2A* | A_24_P1731 | 4205 | 1.04 | 3.03E-01 | 0.95 | 2.54E-01 | 1.02 | 6.61E-01 |
| MAPK signaling | *RPS6KA4* | A_23_P35791 | 8986 | 1.14 | 2.89E-03 | 1.06 | 2.28E-01 | 1.11 | 2.39E-02 |
| MAPK signaling | *JUND* | A_23_P365610 | 3727 | 0.96 | 2.81E-01 | 0.95 | 2.22E-01 | 1.02 | 5.84E-01 |
| MAPK signaling | *RPS6KA5* | A_24_P237601 | 9252 | 1.07 | 2.15E-01 | 0.98 | 7.78E-01 | 1.01 | 9.26E-01 |
| MAPK signaling | *MAPK3* | A_23_P37910 | 5595 | 0.85 | 1.46E-02 | 1.04 | 5.90E-01 | 0.93 | 2.72E-01 |
| MAPK signaling | *EP300* | A_23_P40693 | 2033 | 1.07 | 1.53E-01 | 0.98 | 7.03E-01 | 1.02 | 7.46E-01 |

| | | | | | | | | | |
|---|---|---|---|---|---|---|---|---|---|
| MAPK signaling | *RAF1* | A_23_P40952 | 5894 | 1.03 | 4.06E-01 | 1.02 | 6.95E-01 | 0.99 | 7.72E-01 |
| MAPK signaling | *MAP3K1* | A_23_P41796 | 4214 | 0.99 | 8.04E-01 | 0.95 | 3.81E-01 | 0.94 | 2.44E-01 |
| MAPK signaling | *MAPK14* | A_24_P397566 | 1432 | 1.10 | 6.06E-02 | 1.05 | 3.76E-01 | 1.11 | 4.33E-02 |
| MAPK signaling | *BRAF* | A_23_P42935 | 673 | 0.99 | 6.47E-01 | 1.01 | 8.22E-01 | 0.97 | 3.20E-01 |
| MAPK signaling | *RAP1GAP* | A_24_P36890 | 5909 | 1.10 | 4.76E-01 | 1.02 | 8.74E-01 | 1.20 | 1.97E-01 |
| MAPK signaling | *MAP4K1* | A_23_P5002 | 11184 | 1.11 | 1.60E-02 | 0.97 | 4.86E-01 | 1.04 | 4.39E-01 |
| MAPK signaling | *MAP3K7* | A_23_P500773 | 6885 | 1.04 | 3.08E-01 | 0.95 | 3.22E-01 | 1.01 | 8.95E-01 |
| MAPK signaling | *MAPK11* | A_23_P502274 | 5600 | 0.91 | 1.33E-01 | 1.02 | 7.20E-01 | 0.94 | 3.63E-01 |
| MAPK signaling | *PRKCZ* | A_24_P924462 | 5590 | 1.11 | 2.05E-01 | 0.92 | 2.96E-01 | 1.00 | 9.62E-01 |
| MAPK signaling | *RAP1A* | A_23_P51754 | 5906 | 1.05 | 1.07E-01 | 1.00 | 9.44E-01 | 1.04 | 2.81E-01 |
| MAPK signaling | *DUSP10* | A_23_P51856 | 11221 | 1.13 | 8.10E-02 | 0.93 | 3.00E-01 | 1.01 | 8.58E-01 |
| MAPK signaling | *PPP2R1B* | A_23_P52793 | 5519 | 0.96 | 3.90E-01 | 0.98 | 6.24E-01 | 0.94 | 2.23E-01 |
| MAPK signaling | *PRKCA* | A_24_P916496 | 5578 | 1.07 | 1.91E-01 | 0.97 | 5.85E-01 | 1.00 | 9.80E-01 |
| MAPK signaling | *NRAS* | A_23_P63190 | 4893 | 1.02 | 4.94E-01 | 0.96 | 2.73E-01 | 1.00 | 9.68E-01 |
| MAPK signaling | *BCAR1* | A_23_P77721 | 9564 | 0.96 | 4.41E-01 | 1.05 | 3.45E-01 | 0.98 | 7.65E-01 |
| MAPK signaling | *ARAF* | A_23_P73511 | 369 | 0.99 | 6.08E-01 | 0.98 | 5.82E-01 | 1.02 | 5.14E-01 |
| MAPK signaling | *PAK1* | A_23_P75989 | 5058 | 1.09 | 1.61E-01 | 1.09 | 1.58E-01 | 1.09 | 1.72E-01 |
| MAPK signaling | *TLN1* | A_24_P196851 | 7094 | 1.02 | 6.38E-01 | 0.99 | 7.87E-01 | 1.08 | 1.21E-01 |
| MAPK signaling | *HRAS* | A_23_P98183 | 3265 | 1.02 | 6.42E-01 | 1.02 | 5.36E-01 | 1.00 | 9.77E-01 |
| MAPK signaling | *MAPKAPK5* | A_24_P124662 | 8550 | 1.06 | 3.38E-01 | 0.92 | 1.59E-01 | 1.00 | 9.34E-01 |
| MAPK signaling | *EEF2K* | A_24_P125067 | 29904 | 1.01 | 8.89E-01 | 1.00 | 8.99E-01 | 0.98 | 5.15E-01 |
| MAPK signaling | *MAP3K6* | A_24_P145653 | 9064 | 1.13 | 1.74E-02 | 1.00 | 9.58E-01 | 1.02 | 6.84E-01 |
| MAPK signaling | *YWHAZ* | A_32_P198923 | 7534 | 1.10 | 1.75E-01 | 0.90 | 1.47E-01 | 1.08 | 3.23E-01 |
| MAPK signaling | *TLN2* | A_24_P347566 | 83660 | 0.81 | 7.69E-02 | 1.05 | 6.93E-01 | 0.88 | 2.86E-01 |
| MAPK signaling | *ESR1* | A_24_P383478 | 2099 | 0.91 | 1.06E-01 | 1.00 | 9.41E-01 | 0.95 | 4.38E-01 |
| MAPK signaling | *RPS6KA1* | A_24_P396650 | 6195 | 1.05 | 1.41E-01 | 1.02 | 4.15E-01 | 1.04 | 2.52E-01 |
| MAPK signaling | *HMGN1* | A_32_P22257 | 3150 | 0.93 | 1.57E-01 | 1.01 | 8.56E-01 | 0.94 | 2.36E-01 |
| MAPK signaling | *DUSP9* | A_24_P417189 | 1852 | 0.95 | 1.96E-01 | 1.02 | 7.05E-01 | 0.97 | 4.84E-01 |
| MAPK signaling | *HINT1* | A_32_P47554 | 3094 | 1.08 | 9.47E-02 | 0.98 | 6.13E-01 | 1.07 | 1.66E-01 |
| MAPK signaling | *RAPGEF3* | A_32_P393316 | 10411 | 0.86 | 4.77E-02 | 1.02 | 8.27E-01 | 0.97 | 7.21E-01 |
| MAPK signaling | *MAP2K4* | A_32_P6344 | 6416 | 1.07 | 1.24E-01 | 1.04 | 4.05E-01 | 1.05 | 2.76E-01 |
| MAPK signaling | *PTPRK* | A_32_P99100 | 5796 | 1.05 | 3.83E-01 | 0.95 | 3.86E-01 | 0.91 | 1.15E-01 |
| Adhesion Extravasation Migration | *SELL* | A_23_P103522 | 6402 | 0.98 | 7.14E-01 | 1.06 | 3.21E-01 | 1.10 | 1.08E-01 |
| Adhesion Extravasation Migration | *ITGB1* | A_32_P95397 | 3688 | 1.12 | 4.74E-02 | 0.99 | 7.87E-01 | 1.04 | 4.87E-01 |
| Adhesion Extravasation Migration | *CXCR6* | A_23_P109913 | 10663 | 0.93 | 3.59E-01 | 1.02 | 7.89E-01 | 1.05 | 5.17E-01 |
| Adhesion Extravasation Migration | *CXCL5* | A_23_P110204 | 6374 | 1.06 | 5.98E-01 | 1.08 | 4.35E-01 | 1.19 | 1.22E-01 |
| Adhesion Extravasation Migration | *NT5E* | A_24_P316430 | 4907 | 1.10 | 2.28E-01 | 1.01 | 8.83E-01 | 0.98 | 7.79E-01 |
| Adhesion Extravasation Migration | *CD36* | A_24_P925505 | 948 | 1.00 | 9.95E-01 | 0.97 | 5.40E-01 | 0.98 | 6.78E-01 |
| Adhesion Extravasation Migration | *CCL21* | A_23_P112470 | 6366 | 0.89 | 1.90E-01 | 1.05 | 5.81E-01 | 0.89 | 1.98E-01 |
| Adhesion Extravasation Migration | *DARC* | A_23_P115161 | 2532 | 0.95 | 4.19E-01 | 1.08 | 2.24E-01 | 0.98 | 7.35E-01 |
| Adhesion Extravasation Migration | *CKLF* | A_24_P215804 | 51192 | 0.87 | 4.45E-01 | 1.09 | 5.98E-01 | 1.49 | 2.91E-02 |
| Adhesion Extravasation Migration | *VASP* | A_23_P119102 | 7408 | 1.09 | 1.08E-01 | 0.95 | 3.92E-01 | 1.00 | 9.38E-01 |
| Adhesion Extravasation Migration | *ICAM5* | A_24_P254079 | 7087 | 0.96 | 4.95E-01 | 1.05 | 4.74E-01 | 0.92 | 2.11E-01 |
| Adhesion Extravasation Migration | *JAM2* | A_23_P120667 | 58494 | 0.81 | 4.53E-02 | 1.01 | 9.50E-01 | 0.85 | 1.39E-01 |
| Adhesion Extravasation Migration | *PPBP* | A_23_P121596 | 5473 | 1.03 | 7.07E-01 | 0.97 | 6.75E-01 | 1.14 | 1.17E-01 |
| Adhesion Extravasation Migration | *FYB* | A_24_P393740 | 2533 | 1.10 | 4.40E-02 | 1.01 | 8.48E-01 | 1.00 | 9.65E-01 |
| Adhesion Extravasation Migration | *CCL19* | A_23_P123853 | 6363 | 0.98 | 7.46E-01 | 0.99 | 9.21E-01 | 0.96 | 5.23E-01 |
| Adhesion Extravasation Migration | *ITGAM* | A_23_P124108 | 3684 | 1.09 | 3.43E-02 | 1.04 | 3.22E-01 | 1.03 | 4.41E-01 |
| Adhesion Extravasation Migration | *SIPA1* | A_23_P127460 | 6494 | 1.11 | 2.19E-02 | 0.96 | 3.49E-01 | 1.01 | 8.15E-01 |
| Adhesion Extravasation Migration | *FUT4* | A_24_P295609 | 2526 | 0.99 | 8.95E-01 | 1.00 | 9.87E-01 | 1.02 | 6.62E-01 |
| Adhesion Extravasation Migration | *ITGAD* | A_23_P129665 | 3681 | 1.18 | 1.61E-01 | 1.15 | 2.25E-01 | 1.24 | 8.65E-02 |
| Adhesion Extravasation Migration | *CEACAM3* | A_23_P130515 | 1084 | 1.10 | 3.07E-01 | 1.13 | 1.67E-01 | 1.03 | 7.50E-01 |
| Adhesion Extravasation Migration | *CCL27* | A_23_P135248 | 10850 | 0.92 | 2.37E-01 | 1.01 | 9.02E-01 | 0.92 | 2.18E-01 |
| Adhesion Extravasation Migration | *SELP* | A_23_P137697 | 6403 | 0.97 | 7.23E-01 | 1.01 | 8.70E-01 | 1.14 | 8.79E-02 |
| Adhesion Extravasation Migration | *MUC1* | A_23_P137856 | 4582 | 0.97 | 6.02E-01 | 1.00 | 9.71E-01 | 1.01 | 9.12E-01 |
| Adhesion Extravasation Migration | *CD58* | A_23_P138308 | 965 | 1.06 | 1.02E-01 | 1.04 | 3.17E-01 | 1.03 | 4.81E-01 |
| Adhesion Extravasation Migration | *PXN* | A_23_P13969 | 5829 | 1.00 | 9.44E-01 | 1.05 | 4.21E-01 | 1.08 | 1.82E-01 |
| Adhesion Extravasation Migration | *PTPRU* | A_23_P149064 | 10076 | 0.85 | 4.11E-02 | 0.99 | 9.14E-01 | 0.88 | 1.19E-01 |
| Adhesion Extravasation Migration | *ICAM2* | A_23_P152655 | 3384 | 1.11 | 4.88E-02 | 0.99 | 8.72E-01 | 1.10 | 7.69E-02 |
| Adhesion Extravasation Migration | *CCL5* | A_23_P152838 | 6352 | 1.15 | 2.06E-02 | 1.04 | 5.33E-01 | 1.19 | 5.24E-03 |
| Adhesion Extravasation Migration | *ICAM1* | A_23_P153320 | 3383 | 0.93 | 2.32E-01 | 0.96 | 5.30E-01 | 0.96 | 4.73E-01 |
| Adhesion Extravasation Migration | *CD2* | A_23_P161076 | 914 | 1.15 | 1.13E-02 | 1.00 | 9.34E-01 | 1.05 | 3.54E-01 |
| Adhesion Extravasation Migration | *MYH10* | A_23_P164081 | 4628 | 0.83 | 7.55E-02 | 1.06 | 5.40E-01 | 0.86 | 1.55E-01 |
| Adhesion Extravasation Migration | *PLAUR* | A_23_P16469 | 5329 | 1.04 | 6.53E-01 | 0.90 | 1.89E-01 | 1.01 | 9.05E-01 |
| Adhesion Extravasation Migration | *ICAM3* | A_23_P164691 | 3385 | 1.09 | 4.39E-02 | 1.03 | 5.22E-01 | 1.04 | 3.76E-01 |
| Adhesion Extravasation Migration | *MMP1* | A_23_P1691 | 4312 | 0.89 | 7.90E-02 | 0.94 | 3.32E-01 | 0.97 | 7.06E-01 |
| Adhesion Extravasation Migration | *SIGLEC1* | A_23_P17481 | 6614 | 1.08 | 5.45E-01 | 0.98 | 8.69E-01 | 1.05 | 7.04E-01 |
| Adhesion Extravasation Migration | *XCR1* | A_23_P18246 | 2829 | 0.81 | 4.56E-03 | 1.02 | 7.91E-01 | 0.91 | 2.14E-01 |
| Adhesion Extravasation Migration | *CXCL9* | A_23_P18452 | 4283 | 0.91 | 1.91E-01 | 1.17 | 2.46E-02 | 1.02 | 7.38E-01 |
| Adhesion Extravasation Migration | *RASSF5* | A_24_P171268 | 83593 | 1.07 | 7.03E-02 | 1.01 | 8.59E-01 | 1.05 | 2.58E-01 |
| Adhesion Extravasation Migration | *VCL* | A_24_P47182 | 7414 | 1.06 | 4.10E-01 | 1.02 | 7.88E-01 | 1.14 | 4.72E-02 |
| Adhesion Extravasation Migration | *CXCL12* | A_23_P202448 | 6387 | 1.03 | 5.85E-01 | 0.99 | 8.50E-01 | 0.95 | 2.95E-01 |
| Adhesion Extravasation Migration | *CTTN* | A_23_P202823 | 2017 | 0.91 | 2.26E-01 | 0.96 | 5.66E-01 | 1.02 | 8.16E-01 |
| Adhesion Extravasation Migration | *MMP19* | A_23_P203882 | 4327 | 0.88 | 1.09E-01 | 0.95 | 5.50E-01 | 0.89 | 1.87E-01 |
| Adhesion Extravasation Migration | *ITGAL* | A_23_P206806 | 3683 | 1.14 | 7.48E-03 | 1.03 | 6.16E-01 | 1.11 | 4.07E-02 |
| Adhesion Extravasation Migration | *CCL4* | A_23_P207564 | 6351 | 1.16 | 6.74E-02 | 0.89 | 2.30E-01 | 1.01 | 9.29E-01 |
| Adhesion Extravasation Migration | *ALS2* | A_23_P209430 | 57679 | 1.18 | 6.44E-02 | 1.01 | 9.04E-01 | 1.24 | 1.96E-02 |
| Adhesion Extravasation Migration | *ROCK2* | A_24_P328333 | 9475 | 1.03 | 5.85E-01 | 0.97 | 5.33E-01 | 1.03 | 5.41E-01 |
| Adhesion Extravasation Migration | *ITGA6* | A_23_P210176 | 3655 | 0.99 | 7.07E-01 | 0.98 | 6.56E-01 | 0.93 | 8.93E-02 |
| Adhesion Extravasation Migration | *CCR2* | A_23_P212354 | 729230 | 1.14 | 1.83E-01 | 1.29 | 1.88E-02 | 1.18 | 9.22E-02 |
| Adhesion Extravasation Migration | *CCL24* | A_23_P215491 | 6369 | 0.89 | 3.22E-02 | 1.00 | 9.73E-01 | 0.99 | 9.08E-01 |
| Adhesion Extravasation Migration | *CD99* | A_24_P70993 | 4267 | 1.06 | 1.85E-01 | 0.98 | 6.84E-01 | 1.12 | 8.88E-03 |
| Adhesion Extravasation Migration | *JAM3* | A_23_P217998 | 83700 | 1.01 | 9.08E-01 | 0.96 | 6.56E-01 | 1.21 | 4.29E-02 |

| | | | | | | | | | |
|---|---|---|---|---|---|---|---|---|---|
| Adhesion Extravasation Migration | *ITGAE* | A_23_P218375 | 3682 | 1.04 | 3.30E-01 | 1.01 | 8.94E-01 | 1.10 | 2.42E-02 |
| Adhesion Extravasation Migration | *CCR3* | A_24_P367473 | 1232 | 0.87 | 1.15E-01 | 1.09 | 3.28E-01 | 0.93 | 4.32E-01 |
| Adhesion Extravasation Migration | *CTNND1* | A_23_P251316 | 1500 | 1.13 | 3.42E-02 | 1.02 | 7.93E-01 | 1.02 | 7.54E-01 |
| Adhesion Extravasation Migration | *PECAM1* | A_23_P252471 | 5175 | 1.11 | 4.37E-02 | 1.03 | 5.83E-01 | 1.05 | 3.39E-01 |
| Adhesion Extravasation Migration | *ITGA1* | A_23_P256334 | 3672 | 1.21 | 4.49E-02 | 1.04 | 6.61E-01 | 1.17 | 1.29E-01 |
| Adhesion Extravasation Migration | *CCL17* | A_23_P26325 | 6361 | 0.93 | 1.79E-01 | 1.04 | 4.21E-01 | 0.99 | 8.36E-01 |
| Adhesion Extravasation Migration | *ITGAX* | A_23_P312132 | 3687 | 1.07 | 3.26E-01 | 1.05 | 4.76E-01 | 0.99 | 8.40E-01 |
| Adhesion Extravasation Migration | *ARHGAP1* | A_23_P44057 | 392 | 0.94 | 3.28E-01 | 0.91 | 1.27E-01 | 0.93 | 2.79E-01 |
| Adhesion Extravasation Migration | *CXCL2* | A_23_P315364 | 2920 | 0.80 | 2.96E-01 | 0.51 | 1.61E-03 | 0.78 | 2.41E-01 |
| Adhesion Extravasation Migration | *ITGB2* | A_23_P430411 | 3689 | 0.97 | 6.26E-01 | 1.04 | 4.42E-01 | 1.00 | 9.81E-01 |
| Adhesion Extravasation Migration | *CD33* | A_24_P301655 | 945 | 1.11 | 1.34E-01 | 1.12 | 1.10E-01 | 1.15 | 5.66E-02 |
| Adhesion Extravasation Migration | *CCR7* | A_23_P343398 | 1236 | 1.02 | 7.47E-01 | 0.99 | 9.21E-01 | 1.00 | 9.74E-01 |
| Adhesion Extravasation Migration | *MYL6* | A_23_P344973 | 4637 | 1.00 | 9.41E-01 | 1.01 | 6.66E-01 | 1.01 | 8.07E-01 |
| Adhesion Extravasation Migration | *THY1* | A_23_P36364 | 7070 | 0.74 | 4.54E-03 | 1.07 | 5.32E-01 | 0.90 | 3.40E-01 |
| Adhesion Extravasation Migration | *ITGA5* | A_23_P36562 | 3678 | 0.98 | 6.64E-01 | 1.02 | 6.06E-01 | 0.98 | 6.81E-01 |
| Adhesion Extravasation Migration | *CEACAM8* | A_23_P380240 | 1088 | 1.07 | 4.41E-01 | 1.13 | 1.63E-01 | 0.99 | 9.08E-01 |
| Adhesion Extravasation Migration | *CXCL16* | A_23_P38505 | 58191 | 0.97 | 5.22E-01 | 1.03 | 5.42E-01 | 0.94 | 2.41E-01 |
| Adhesion Extravasation Migration | *ITGB3* | A_24_P318656 | 3690 | 1.08 | 4.55E-01 | 0.94 | 5.01E-01 | 1.17 | 1.31E-01 |
| Adhesion Extravasation Migration | *MMP9* | A_23_P40174 | 4318 | 1.01 | 8.64E-01 | 0.99 | 9.22E-01 | 1.01 | 9.10E-01 |
| Adhesion Extravasation Migration | *CX3CR1* | A_23_P407565 | 1524 | 1.21 | 4.74E-02 | 1.11 | 2.91E-01 | 1.17 | 1.06E-01 |
| Adhesion Extravasation Migration | *ALCAM* | A_32_P74643 | 214 | 0.99 | 8.36E-01 | 1.08 | 1.60E-01 | 1.08 | 2.17E-01 |
| Adhesion Extravasation Migration | *CCR5* | A_23_P412321 | 1234 | 1.08 | 2.65E-01 | 1.09 | 1.75E-01 | 1.12 | 1.09E-01 |
| Adhesion Extravasation Migration | *SPN* | A_24_P407645 | 6693 | 1.10 | 1.59E-02 | 1.04 | 3.18E-01 | 1.12 | 1.12E-02 |
| Adhesion Extravasation Migration | *CEACAM1* | A_23_P55738 | 634 | 0.85 | 6.83E-02 | 1.08 | 3.81E-01 | 0.92 | 3.64E-01 |
| Adhesion Extravasation Migration | *CD96* | A_23_P44154 | 10225 | 1.09 | 7.21E-02 | 0.95 | 3.61E-01 | 1.01 | 7.90E-01 |
| Adhesion Extravasation Migration | *CD97* | A_23_P502312 | 976 | 1.10 | 6.04E-02 | 1.00 | 9.78E-01 | 1.03 | 6.13E-01 |
| Adhesion Extravasation Migration | *CCL28* | A_23_P503072 | 56477 | 1.07 | 2.16E-01 | 0.89 | 2.81E-02 | 0.98 | 6.64E-01 |
| Adhesion Extravasation Migration | *ITGAV* | A_23_P50907 | 3685 | 1.09 | 4.41E-02 | 1.06 | 2.18E-01 | 1.04 | 3.87E-01 |
| Adhesion Extravasation Migration | *ITGA4* | A_23_P56505 | 3676 | 1.09 | 1.03E-01 | 0.93 | 2.11E-01 | 1.04 | 5.40E-01 |
| Adhesion Extravasation Migration | *MYH9* | A_24_P408424 | 4627 | 1.06 | 2.20E-01 | 0.94 | 2.23E-01 | 0.99 | 8.84E-01 |
| Adhesion Extravasation Migration | *RHOH* | A_23_P58132 | 399 | 1.06 | 2.12E-01 | 0.93 | 1.50E-01 | 0.98 | 6.66E-01 |
| Adhesion Extravasation Migration | *SELPLG* | A_23_P64860 | 6404 | 1.13 | 4.56E-03 | 1.04 | 3.13E-01 | 1.06 | 1.98E-01 |
| Adhesion Extravasation Migration | *ITGB4* | A_23_P66355 | 3691 | 0.98 | 7.56E-01 | 1.04 | 4.61E-01 | 0.98 | 7.15E-01 |
| Adhesion Extravasation Migration | *CCR1* | A_24_P148717 | 1230 | 1.03 | 7.05E-01 | 1.06 | 5.04E-01 | 1.08 | 3.50E-01 |
| Adhesion Extravasation Migration | *CCRL2* | A_24_P97405 | 9034 | 0.90 | 2.64E-01 | 1.09 | 3.26E-01 | 1.11 | 2.59E-01 |
| Adhesion Extravasation Migration | *CD47* | A_24_P943792 | 961 | 0.98 | 6.93E-01 | 0.97 | 5.46E-01 | 1.05 | 3.89E-01 |
| Adhesion Extravasation Migration | *RHOA* | A_23_P69493 | 387 | 1.10 | 2.75E-02 | 0.95 | 2.70E-01 | 1.04 | 4.29E-01 |
| Adhesion Extravasation Migration | *HMMR* | A_23_P70007 | 3161 | 0.93 | 2.15E-01 | 1.05 | 3.76E-01 | 1.01 | 8.92E-01 |
| Adhesion Extravasation Migration | *CXCL1* | A_23_P7144 | 2919 | 0.91 | 3.52E-01 | 0.92 | 4.53E-01 | 0.86 | 1.58E-01 |
| Adhesion Extravasation Migration | *CCR4* | A_23_P72989 | 1233 | 0.98 | 6.99E-01 | 1.06 | 1.93E-01 | 1.02 | 6.48E-01 |
| Adhesion Extravasation Migration | *CD48* | A_23_P74145 | 962 | 1.12 | 1.80E-02 | 0.97 | 5.95E-01 | 1.06 | 2.71E-01 |
| Adhesion Extravasation Migration | *CD9* | A_23_P76364 | 928 | 1.01 | 8.94E-01 | 0.96 | 6.40E-01 | 1.11 | 2.25E-01 |
| Adhesion Extravasation Migration | *ITGB7* | A_23_P76529 | 3695 | 1.12 | 1.33E-02 | 0.96 | 4.09E-01 | 1.02 | 6.18E-01 |
| Adhesion Extravasation Migration | *ITGA2B* | A_23_P77971 | 3674 | 1.05 | 5.56E-01 | 1.02 | 8.47E-01 | 1.22 | 2.38E-02 |
| Adhesion Extravasation Migration | *CD226* | A_23_P78330 | 10666 | 1.03 | 6.04E-01 | 0.96 | 4.39E-01 | 1.07 | 2.11E-01 |
| Adhesion Extravasation Migration | *CRK* | A_24_P270814 | 1398 | 0.95 | 5.07E-01 | 1.02 | 8.39E-01 | 0.97 | 6.64E-01 |
| Adhesion Extravasation Migration | *CCL2* | A_23_P89431 | 6347 | 0.83 | 1.61E-02 | 1.02 | 8.26E-01 | 0.89 | 1.53E-01 |
| Adhesion Extravasation Migration | *MIF* | A_23_P91619 | 4282 | 1.09 | 1.88E-03 | 1.02 | 4.05E-01 | 1.07 | 2.24E-02 |
| Adhesion Extravasation Migration | *CCBP2* | A_23_P91954 | 1238 | 0.95 | 4.08E-01 | 0.99 | 8.88E-01 | 0.99 | 8.38E-01 |
| Adhesion Extravasation Migration | *CXCL3* | A_24_P183150 | 2921 | 0.83 | 1.45E-01 | 0.80 | 9.66E-02 | 1.16 | 2.61E-01 |
| Adhesion Extravasation Migration | *CCR6* | A_24_P234921 | 1235 | 0.97 | 6.98E-01 | 1.02 | 8.03E-01 | 1.11 | 2.02E-01 |
| Adhesion Extravasation Migration | *ADAM8* | A_24_P300777 | 101 | 1.12 | 4.36E-02 | 1.02 | 7.01E-01 | 1.03 | 5.48E-01 |
| Adhesion Extravasation Migration | *CCL15* | A_24_P301501 | 6359 | 1.14 | 1.28E-01 | 0.98 | 8.16E-01 | 0.94 | 4.90E-01 |
| Adhesion Extravasation Migration | *CCL23* | A_24_P319088 | 6368 | 0.95 | 3.96E-01 | 0.94 | 3.85E-01 | 0.98 | 7.75E-01 |
| Adhesion Extravasation Migration | *F11R* | A_24_P319364 | 50848 | 1.08 | 8.73E-02 | 1.02 | 7.48E-01 | 1.11 | 3.61E-02 |
| Adhesion Extravasation Migration | *CX3CL1* | A_24_P390495 | 6376 | 0.76 | 6.98E-03 | 1.11 | 2.85E-01 | 0.90 | 3.40E-01 |
| Adhesion Extravasation Migration | *XCL1* | A_24_P45476 | 6375 | 1.15 | 7.70E-02 | 1.04 | 6.88E-01 | 1.12 | 1.71E-01 |
| Adhesion Extravasation Migration | *MMP14* | A_24_P82106 | 4323 | 1.05 | 4.48E-01 | 1.00 | 9.91E-01 | 1.03 | 6.40E-01 |
| Adhesion Extravasation Migration | *ITGA2* | A_32_P208076 | 3673 | 1.11 | 2.00E-01 | 0.95 | 5.34E-01 | 1.23 | 1.53E-02 |
| Leukocyte signaling | *MARCO* | A_23_P101992 | 8685 | 1.17 | 9.21E-02 | 1.25 | 1.43E-02 | 1.30 | 7.86E-03 |
| Leukocyte signaling | *CTLA4* | A_23_P102481 | 1493 | 0.99 | 9.13E-01 | 1.01 | 7.53E-01 | 1.00 | 9.53E-01 |
| Leukocyte signaling | *LCK* | A_23_P103361 | 3932 | 1.15 | 4.95E-03 | 1.00 | 9.64E-01 | 1.05 | 3.86E-01 |
| Leukocyte signaling | *FCER1A* | A_23_P103765 | 2205 | 1.03 | 5.94E-01 | 1.09 | 1.48E-01 | 1.01 | 8.60E-01 |
| Leukocyte signaling | *PIK3AP1* | A_23_P104445 | 118788 | 1.07 | 9.97E-02 | 1.02 | 6.93E-01 | 1.09 | 4.82E-02 |
| Leukocyte signaling | *CD79A* | A_23_P107735 | 973 | 0.86 | 8.78E-03 | 1.04 | 4.95E-01 | 0.97 | 6.56E-01 |
| Leukocyte signaling | *LILRA5* | A_24_P370172 | 353514 | 1.15 | 3.95E-02 | 1.12 | 1.15E-01 | 1.17 | 2.50E-02 |
| Leukocyte signaling | *CD86* | A_24_P131589 | 942 | 1.26 | 5.95E-02 | 0.94 | 6.50E-01 | 1.06 | 6.67E-01 |
| Leukocyte signaling | *SH3BP2* | A_24_P226020 | 6452 | 0.90 | 7.86E-02 | 1.00 | 9.49E-01 | 0.93 | 2.87E-01 |
| Leukocyte signaling | *MS4A1* | A_23_P116371 | 931 | 1.08 | 2.25E-01 | 0.93 | 2.83E-01 | 0.91 | 1.61E-01 |
| Leukocyte signaling | *IGLL1* | A_24_P83102 | 3543 | 1.18 | 4.75E-02 | 1.00 | 9.81E-01 | 1.02 | 8.01E-01 |
| Leukocyte signaling | *PTPRC* | A_23_P12392 | 5788 | 1.07 | 2.03E-01 | 1.01 | 9.17E-01 | 1.01 | 8.39E-01 |
| Leukocyte signaling | *IGHA1* | A_23_P124632 | 3493 | 1.11 | 1.42E-01 | 0.96 | 5.58E-01 | 1.01 | 8.36E-01 |
| Leukocyte signaling | *SCARF2* | A_24_P108738 | 91179 | 0.96 | 4.89E-01 | 1.13 | 4.00E-02 | 1.19 | 4.17E-03 |
| Leukocyte signaling | *BTK* | A_23_P137139 | 695 | 1.06 | 2.55E-01 | 1.01 | 8.84E-01 | 1.08 | 1.26E-01 |
| Leukocyte signaling | *LILRA2* | A_23_P142205 | 11027 | 1.11 | 1.94E-01 | 1.17 | 4.36E-02 | 1.21 | 1.99E-02 |
| Leukocyte signaling | *SLA2* | A_23_P143173 | 84174 | 1.01 | 7.80E-01 | 0.95 | 3.45E-01 | 1.10 | 8.23E-02 |
| Leukocyte signaling | *GRAP2* | A_24_P408449 | 9402 | 1.08 | 2.49E-01 | 0.96 | 5.00E-01 | 1.09 | 1.93E-01 |
| Leukocyte signaling | *PTPN22* | A_23_P149345 | 26191 | 1.07 | 2.06E-01 | 0.98 | 6.42E-01 | 1.06 | 3.20E-01 |
| Leukocyte signaling | *SCGB1A1* | A_23_P150583 | 7356 | 0.76 | 1.35E-02 | 1.12 | 2.93E-01 | 0.91 | 4.38E-01 |
| Leukocyte signaling | *CSK* | A_23_P152024 | 1445 | 1.07 | 6.03E-02 | 0.98 | 6.56E-01 | 1.02 | 5.51E-01 |
| Leukocyte signaling | *CD68* | A_23_P15394 | 968 | 1.08 | 1.79E-01 | 1.02 | 7.16E-01 | 1.12 | 4.92E-02 |
| Leukocyte signaling | *SCARF1* | A_23_P15414 | 8578 | 1.07 | 1.91E-01 | 1.03 | 5.21E-01 | 1.06 | 2.99E-01 |
| Leukocyte signaling | *FOXP3* | A_23_P159709 | 50943 | 0.91 | 2.42E-01 | 0.95 | 5.12E-01 | 0.91 | 2.40E-01 |

| | | | | | | | | | |
|---|---|---|---|---|---|---|---|---|---|
| Leukocyte signaling | *SIGLEC5* | A_24_P48539 | 8778 | 0.87 | 8.44E-02 | 1.08 | 3.13E-01 | 0.98 | 7.95E-01 |
| Leukocyte signaling | *FCER1G* | A_23_P160849 | 2207 | 0.85 | 1.57E-02 | 1.09 | 2.01E-01 | 0.99 | 9.41E-01 |
| Leukocyte signaling | *FCER2* | A_23_P164773 | 2208 | 0.97 | 7.17E-01 | 0.94 | 4.29E-01 | 1.08 | 3.75E-01 |
| Leukocyte signaling | *ICOSLG* | A_23_P317667 | 23308 | 0.88 | 1.62E-01 | 1.08 | 4.00E-01 | 0.94 | 4.97E-01 |
| Leukocyte signaling | *CD38* | A_23_P167328 | 952 | 0.99 | 9.02E-01 | 0.94 | 2.25E-01 | 1.01 | 8.93E-01 |
| Leukocyte signaling | *MAL* | A_23_P17134 | 4118 | 1.06 | 2.27E-01 | 0.98 | 6.14E-01 | 1.01 | 9.00E-01 |
| Leukocyte signaling | *SIRPB1* | A_24_P143574 | 10326 | 1.01 | 7.93E-01 | 1.05 | 2.89E-01 | 1.02 | 7.18E-01 |
| Leukocyte signaling | *MS4A2* | A_23_P1904 | 2206 | 1.01 | 8.87E-01 | 1.02 | 7.81E-01 | 1.05 | 5.93E-01 |
| Leukocyte signaling | *TREM1* | A_23_P19333 | 54210 | 1.05 | 5.87E-01 | 0.99 | 8.87E-01 | 1.04 | 6.48E-01 |
| Leukocyte signaling | *PILRB* | A_23_P19829 | 29990 | 1.09 | 1.39E-01 | 0.95 | 4.12E-01 | 1.03 | 6.66E-01 |
| Leukocyte signaling | *PTPN7* | A_23_P201778 | 5778 | 1.16 | 4.42E-03 | 0.99 | 7.77E-01 | 1.08 | 1.29E-01 |
| Leukocyte signaling | *CD79B* | A_23_P207201 | 974 | 1.06 | 3.72E-01 | 0.98 | 7.51E-01 | 0.98 | 8.10E-01 |
| Leukocyte signaling | *SECTM1* | A_24_P48204 | 6398 | 1.13 | 6.65E-02 | 1.08 | 2.63E-01 | 1.14 | 6.26E-02 |
| Leukocyte signaling | *SIGLEC10* | A_23_P208182 | 89790 | 1.09 | 1.98E-01 | 1.00 | 9.56E-01 | 1.02 | 7.48E-01 |
| Leukocyte signaling | *LILRB2* | A_23_P208493 | 10288 | 1.14 | 3.74E-02 | 1.12 | 1.21E-01 | 1.10 | 1.29E-01 |
| Leukocyte signaling | *FCAR* | A_24_P348265 | 2204 | 0.99 | 9.15E-01 | 0.91 | 2.82E-01 | 0.95 | 5.98E-01 |
| Leukocyte signaling | *CD22* | A_24_P254106 | 933 | 1.09 | 2.88E-01 | 0.95 | 4.98E-01 | 1.02 | 8.20E-01 |
| Leukocyte signaling | *TEC* | A_23_P213114 | 7006 | 0.93 | 1.66E-01 | 0.97 | 4.99E-01 | 0.95 | 3.79E-01 |
| Leukocyte signaling | *CD44* | A_23_P24870 | 960 | 1.10 | 4.54E-03 | 0.99 | 8.09E-01 | 1.07 | 8.03E-02 |
| Leukocyte signaling | *DAPP1* | A_23_P255444 | 27071 | 0.98 | 5.22E-01 | 1.02 | 5.43E-01 | 1.05 | 1.78E-01 |
| Leukocyte signaling | *MST1R* | A_23_P256312 | 4486 | 0.92 | 3.00E-01 | 1.12 | 1.58E-01 | 1.05 | 5.60E-01 |
| Leukocyte signaling | *SHB* | A_24_P146575 | 6461 | 0.95 | 4.22E-01 | 1.03 | 6.48E-01 | 0.96 | 6.09E-01 |
| Leukocyte signaling | *LILRP2* | A_23_P27781 | 79166 | 0.98 | 6.74E-01 | 1.08 | 5.34E-02 | 1.07 | 1.13E-01 |
| Leukocyte signaling | *SIRPG* | A_23_P28857 | 55423 | 1.05 | 2.64E-01 | 0.99 | 8.71E-01 | 0.97 | 4.67E-01 |
| Leukocyte signaling | *VPREB1* | A_23_P29152 | 7441 | 0.79 | 1.01E-02 | 0.94 | 4.88E-01 | 0.83 | 4.54E-02 |
| Leukocyte signaling | *TXK* | A_24_P921122 | 7294 | 1.07 | 2.53E-01 | 0.92 | 1.35E-01 | 0.98 | 6.79E-01 |
| Leukocyte signaling | *SCGB3A1* | A_23_P30217 | 92304 | 0.98 | 8.68E-01 | 1.04 | 7.23E-01 | 1.10 | 3.80E-01 |
| Leukocyte signaling | *LCP2* | A_23_P30547 | 3937 | 1.09 | 5.84E-02 | 0.97 | 4.93E-01 | 1.05 | 3.33E-01 |
| Leukocyte signaling | *CBL* | A_23_P53226 | 867 | 1.12 | 7.52E-02 | 1.05 | 4.19E-01 | 1.04 | 5.23E-01 |
| Leukocyte signaling | *FCGR2B* | A_23_P34644 | 2213 | 1.01 | 8.47E-01 | 0.97 | 6.16E-01 | 0.98 | 7.43E-01 |
| Leukocyte signaling | *PAG1* | A_32_P61684 | 55824 | 1.04 | 4.36E-01 | 1.01 | 9.13E-01 | 1.04 | 4.11E-01 |
| Leukocyte signaling | *SH2D1B* | A_23_P351148 | 117157 | 1.05 | 5.82E-01 | 1.06 | 4.85E-01 | 1.09 | 3.00E-01 |
| Leukocyte signaling | *ITK* | A_23_P354151 | 3702 | 1.07 | 2.44E-01 | 0.99 | 8.72E-01 | 0.99 | 8.75E-01 |
| Leukocyte signaling | *LRRC23* | A_23_P36689 | 10233 | 1.06 | 3.68E-01 | 0.91 | 8.68E-02 | 1.00 | 9.58E-01 |
| Leukocyte signaling | *LILRB3* | A_32_P70158 | 11025 | 1.10 | 1.74E-01 | 1.05 | 5.03E-01 | 1.02 | 7.40E-01 |
| Leukocyte signaling | *TFEB* | A_23_P368729 | 7942 | 1.05 | 1.10E-01 | 1.02 | 5.28E-01 | 1.00 | 9.07E-01 |
| Leukocyte signaling | *ICOS* | A_23_P371215 | 29851 | 1.03 | 4.68E-01 | 0.98 | 6.87E-01 | 0.99 | 8.08E-01 |
| Leukocyte signaling | *SLC7A5* | A_23_P3792 | 8140 | 0.94 | 3.22E-01 | 1.00 | 9.90E-01 | 0.96 | 5.15E-01 |
| Leukocyte signaling | *VAV1* | A_23_P38959 | 7409 | 1.13 | 7.44E-03 | 1.01 | 8.66E-01 | 1.04 | 3.91E-01 |
| Leukocyte signaling | *ZAP70* | A_23_P39682 | 7535 | 1.19 | 5.28E-03 | 0.96 | 5.06E-01 | 1.02 | 7.38E-01 |
| Leukocyte signaling | *PTPRJ* | A_23_P405049 | 5795 | 1.06 | 2.40E-01 | 0.97 | 5.18E-01 | 1.09 | 1.15E-01 |
| Leukocyte signaling | *CD3E* | A_24_P86968 | 916 | 1.05 | 2.86E-01 | 0.95 | 2.78E-01 | 0.97 | 4.86E-01 |
| Leukocyte signaling | *LAT* | A_23_P44105 | 27040 | 1.13 | 2.68E-02 | 1.00 | 9.40E-01 | 1.07 | 2.34E-01 |
| Leukocyte signaling | *FYN* | A_23_P502142 | 2534 | 1.09 | 6.97E-02 | 0.98 | 5.99E-01 | 1.04 | 3.55E-01 |
| Leukocyte signaling | *PTAFR* | A_23_P51926 | 5724 | 1.14 | 6.56E-02 | 1.11 | 1.70E-01 | 1.08 | 2.94E-01 |
| Leukocyte signaling | *CD40* | A_23_P57036 | 958 | 1.03 | 5.27E-01 | 0.97 | 4.97E-01 | 0.98 | 6.36E-01 |
| Leukocyte signaling | *ABL1* | A_24_P282416 | 25 | 1.11 | 1.52E-01 | 0.91 | 2.42E-01 | 1.08 | 3.35E-01 |
| Leukocyte signaling | *CD40LG* | A_23_P62220 | 959 | 1.09 | 7.55E-02 | 1.05 | 3.02E-01 | 1.06 | 2.51E-01 |
| Leukocyte signaling | *SLAMF1* | A_23_P62652 | 6504 | 1.11 | 9.46E-02 | 1.03 | 6.41E-01 | 1.09 | 1.71E-01 |
| Leukocyte signaling | *CST7* | A_23_P68601 | 8530 | 1.11 | 9.03E-02 | 1.00 | 9.55E-01 | 1.19 | 8.75E-03 |
| Leukocyte signaling | *CD53* | A_23_P74547 | 963 | 1.06 | 2.01E-01 | 0.97 | 5.26E-01 | 1.03 | 5.24E-01 |
| Leukocyte signaling | *TCIRG1* | A_23_P75369 | 10312 | 1.10 | 7.06E-02 | 0.98 | 7.29E-01 | 1.02 | 6.73E-01 |
| Leukocyte signaling | *SLC3A2* | A_23_P75811 | 6520 | 1.03 | 3.67E-01 | 1.01 | 7.41E-01 | 1.03 | 4.04E-01 |
| Leukocyte signaling | *TCF7* | A_23_P7582 | 6932 | 1.12 | 4.50E-02 | 0.95 | 3.92E-01 | 0.94 | 2.72E-01 |
| Leukocyte signaling | *GRB2* | A_24_P407717 | 2885 | 1.09 | 6.38E-02 | 0.99 | 8.96E-01 | 1.09 | 7.95E-02 |
| Leukocyte signaling | *FLT3LG* | A_23_P78742 | 2323 | 1.05 | 2.28E-01 | 1.01 | 7.68E-01 | 1.08 | 9.36E-02 |
| Leukocyte signaling | *LILRA3* | A_23_P79094 | 11026 | 1.18 | 2.11E-02 | 1.17 | 2.28E-02 | 1.23 | 3.77E-03 |
| Leukocyte signaling | *FCGR2A* | A_23_P85716 | 2212 | 1.01 | 7.97E-01 | 1.03 | 5.81E-01 | 0.99 | 7.84E-01 |
| Leukocyte signaling | *CD52* | A_23_P85800 | 1043 | 1.17 | 3.10E-03 | 0.99 | 8.34E-01 | 1.09 | 1.01E-01 |
| Leukocyte signaling | *LILRA4* | A_23_P90497 | 23547 | 1.02 | 6.34E-01 | 1.07 | 1.35E-01 | 1.12 | 1.72E-02 |
| Leukocyte signaling | *CD28* | A_23_P91095 | 940 | 1.05 | 3.73E-01 | 0.99 | 9.11E-01 | 1.01 | 9.22E-01 |
| Leukocyte signaling | *SYK* | A_23_P9255 | 6850 | 1.08 | 1.32E-01 | 0.99 | 8.54E-01 | 1.03 | 4.93E-01 |
| Leukocyte signaling | *SLAMF9* | A_23_P96833 | 89886 | 0.83 | 9.41E-03 | 1.05 | 4.61E-01 | 0.93 | 3.42E-01 |
| Leukocyte signaling | *FLT3* | A_23_P99442 | 2322 | 0.98 | 7.95E-01 | 1.00 | 9.69E-01 | 1.02 | 7.16E-01 |
| Leukocyte signaling | *TACR1* | A_24_P148590 | 6869 | 0.79 | 6.77E-02 | 1.13 | 3.34E-01 | 1.01 | 9.62E-01 |
| Leukocyte signaling | *EDNRA* | A_24_P217572 | 1909 | 0.97 | 7.95E-01 | 1.07 | 4.95E-01 | 1.09 | 4.58E-01 |
| Leukocyte signaling | *SEMA4D* | A_24_P261169 | 10507 | 1.06 | 3.19E-01 | 0.98 | 7.77E-01 | 1.02 | 7.62E-01 |
| Leukocyte signaling | *CD4* | A_24_P295999 | 920 | 0.84 | 4.97E-03 | 1.07 | 2.60E-01 | 0.98 | 7.46E-01 |
| Leukocyte signaling | *CD80* | A_24_P320033 | 941 | 0.94 | 4.46E-01 | 0.99 | 8.81E-01 | 0.92 | 3.08E-01 |
| Leukocyte signaling | *LILRA1* | A_24_P393151 | 11024 | 1.06 | 3.92E-01 | 1.11 | 1.72E-01 | 1.11 | 1.02E-01 |
| Leukocyte signaling | *SLAMF7* | A_24_P353638 | 57823 | 1.14 | 2.60E-02 | 0.99 | 8.95E-01 | 1.12 | 5.09E-02 |
| Leukocyte signaling | *CD5* | A_24_P364221 | 921 | 1.00 | 9.55E-01 | 0.95 | 1.09E-01 | 0.95 | 1.29E-01 |
| Leukocyte signaling | *SLAMF6* | A_24_P45451 | 114836 | 1.06 | 3.30E-01 | 0.99 | 8.29E-01 | 1.07 | 2.59E-01 |
| Leukocyte signaling | *BLNK* | A_24_P64344 | 29760 | 1.05 | 5.15E-01 | 0.95 | 5.02E-01 | 1.00 | 9.99E-01 |
| Leukocyte signaling | *LILRB4* | A_24_P65722 | 11006 | 0.91 | 1.12E-01 | 1.10 | 9.67E-02 | 0.99 | 8.96E-01 |
| Leukocyte signaling | *CD37* | A_24_P82749 | 951 | 1.08 | 1.16E-01 | 0.95 | 2.87E-01 | 1.02 | 7.50E-01 |
| Leukocyte signaling | *PAX5* | A_24_P916522 | 5079 | 0.93 | 1.67E-01 | 0.97 | 5.52E-01 | 0.97 | 5.85E-01 |
| Leukocyte signaling | *DPP4* | A_24_P97104 | 1803 | 0.90 | 1.09E-01 | 0.97 | 6.25E-01 | 0.91 | 1.89E-01 |
| Leukocyte signaling | *CD8A* | A_32_P163247 | 925 | 1.13 | 8.52E-02 | 0.97 | 6.49E-01 | 1.01 | 8.84E-01 |
| Apoptosis Signaling | *BCL2L13* | A_24_P410389 | 23786 | 0.99 | 7.27E-01 | 1.06 | 1.49E-01 | 0.97 | 3.99E-01 |
| Apoptosis Signaling | *PARP1* | A_23_P114783 | 142 | 1.01 | 7.95E-01 | 1.00 | 9.60E-01 | 1.01 | 6.86E-01 |
| Apoptosis Signaling | *TNFSF10* | A_23_P121253 | 8743 | 1.10 | 1.07E-01 | 1.10 | 1.14E-01 | 1.09 | 1.57E-01 |
| Apoptosis Signaling | *DFFA* | A_23_P12189 | 1676 | 0.94 | 2.45E-01 | 1.07 | 2.44E-01 | 0.96 | 5.17E-01 |

| | | | | | | | | | |
|---|---|---|---|---|---|---|---|---|---|
| Apoptosis Signaling | *DAXX* | A_23_P122579 | 1616 | 1.05 | 2.57E-01 | 1.01 | 7.37E-01 | 1.04 | 2.75E-01 |
| Apoptosis Signaling | *CASP7* | A_23_P12572 | 840 | 1.13 | 1.80E-02 | 1.02 | 6.92E-01 | 1.13 | 2.67E-02 |
| Apoptosis Signaling | *BCL2L14* | A_23_P128050 | 79370 | 0.97 | 6.85E-01 | 1.06 | 3.77E-01 | 1.10 | 1.88E-01 |
| Apoptosis Signaling | *ROCK1* | A_24_P538403 | 6093 | 1.05 | 2.26E-01 | 0.98 | 6.84E-01 | 1.03 | 5.02E-01 |
| Apoptosis Signaling | *CAPN1* | A_24_P544543 | 823 | 1.07 | 1.57E-01 | 0.94 | 1.78E-01 | 1.02 | 7.10E-01 |
| Apoptosis Signaling | *ACIN1* | A_23_P14389 | 22985 | 1.06 | 1.25E-01 | 0.95 | 1.79E-01 | 1.01 | 8.54E-01 |
| Apoptosis Signaling | *BAK1* | A_23_P145357 | 578 | 1.05 | 6.80E-02 | 1.00 | 9.70E-01 | 1.02 | 5.29E-01 |
| Apoptosis Signaling | *RIPK3* | A_23_P14559 | 11035 | 1.09 | 6.59E-02 | 1.03 | 5.71E-01 | 1.03 | 5.97E-01 |
| Apoptosis Signaling | *BAD* | A_23_P150207 | 572 | 0.98 | 4.79E-01 | 0.98 | 5.11E-01 | 0.99 | 6.63E-01 |
| Apoptosis Signaling | *BCL2A1* | A_23_P152002 | 597 | 1.08 | 2.79E-01 | 0.95 | 4.86E-01 | 1.00 | 9.50E-01 |
| Apoptosis Signaling | *TANK* | A_23_P154306 | 10010 | 0.95 | 2.05E-01 | 0.95 | 2.54E-01 | 0.94 | 1.64E-01 |
| Apoptosis Signaling | *BCL2L11* | A_24_P122921 | 10018 | 1.06 | 2.37E-01 | 1.03 | 5.90E-01 | 1.09 | 9.21E-02 |
| Apoptosis Signaling | *BID* | A_24_P187948 | 637 | 1.09 | 7.33E-02 | 0.99 | 8.73E-01 | 1.07 | 1.74E-01 |
| Apoptosis Signaling | *TNFRSF10B* | A_24_P218265 | 8795 | 1.03 | 4.87E-01 | 1.01 | 8.37E-01 | 1.09 | 7.63E-02 |
| Apoptosis Signaling | *PTPN13* | A_23_P18493 | 5783 | 0.98 | 8.05E-01 | 0.96 | 5.49E-01 | 0.98 | 8.52E-01 |
| Apoptosis Signaling | *BCL2* | A_23_P208132 | 596 | 0.92 | 1.68E-01 | 1.03 | 6.43E-01 | 0.95 | 3.77E-01 |
| Apoptosis Signaling | *SPTAN1* | A_23_P20832 | 6709 | 1.01 | 5.67E-01 | 1.03 | 3.20E-01 | 1.02 | 4.76E-01 |
| Apoptosis Signaling | *BAX* | A_23_P346309 | 581 | 1.08 | 7.89E-02 | 1.07 | 1.61E-01 | 1.12 | 1.67E-02 |
| Apoptosis Signaling | *CASP8* | A_24_P157087 | 841 | 1.00 | 9.96E-01 | 1.03 | 5.27E-01 | 1.03 | 4.55E-01 |
| Apoptosis Signaling | *CFLAR* | A_24_P120115 | 8837 | 1.05 | 3.30E-01 | 1.02 | 7.08E-01 | 1.04 | 4.27E-01 |
| Apoptosis Signaling | *CASP10* | A_24_P3045 | 843 | 0.95 | 3.26E-01 | 1.00 | 9.88E-01 | 0.93 | 2.23E-01 |
| Apoptosis Signaling | *BCL2L1* | A_24_P100130 | 598 | 0.89 | 6.40E-02 | 1.06 | 3.32E-01 | 1.03 | 6.37E-01 |
| Apoptosis Signaling | *CAPN2* | A_23_P23924 | 824 | 1.07 | 7.72E-02 | 0.99 | 7.55E-01 | 1.04 | 3.16E-01 |
| Apoptosis Signaling | *RIPK2* | A_23_P252106 | 8767 | 1.03 | 5.67E-01 | 0.89 | 5.68E-02 | 0.97 | 6.14E-01 |
| Apoptosis Signaling | *DAPK1* | A_23_P252163 | 1612 | 1.04 | 4.38E-01 | 1.15 | 2.21E-02 | 1.10 | 8.35E-02 |
| Apoptosis Signaling | *FAIM* | A_23_P253932 | 55179 | 1.09 | 7.48E-02 | 0.99 | 8.25E-01 | 1.04 | 4.45E-01 |
| Apoptosis Signaling | *TNFRSF10A* | A_23_P255653 | 8797 | 1.11 | 1.65E-02 | 0.99 | 9.02E-01 | 1.01 | 8.64E-01 |
| Apoptosis Signaling | *TNFRSF10C* | A_23_P256724 | 8794 | 1.19 | 1.78E-02 | 1.02 | 7.59E-01 | 1.02 | 7.57E-01 |
| Apoptosis Signaling | *TP53* | A_23_P26810 | 7157 | 1.02 | 7.16E-01 | 1.01 | 9.08E-01 | 1.04 | 4.92E-01 |
| Apoptosis Signaling | *TNFRSF21* | A_23_P30666 | 27242 | 0.96 | 4.58E-01 | 1.01 | 7.66E-01 | 0.98 | 6.46E-01 |
| Apoptosis Signaling | *CAPN10* | A_23_P341349 | 11132 | 1.09 | 1.73E-01 | 1.09 | 1.74E-01 | 1.13 | 5.71E-02 |
| Apoptosis Signaling | *LMNA* | A_23_P34835 | 4000 | 1.07 | 2.25E-01 | 1.01 | 8.61E-01 | 1.12 | 4.42E-02 |
| Apoptosis Signaling | *APAF1* | A_23_P36611 | 317 | 1.09 | 1.95E-01 | 1.08 | 2.48E-01 | 1.08 | 2.71E-01 |
| Apoptosis Signaling | *FASLG* | A_23_P369815 | 356 | 1.03 | 6.55E-01 | 1.08 | 2.99E-01 | 1.12 | 1.43E-01 |
| Apoptosis Signaling | *BBC3* | A_24_P305312 | 27113 | 0.83 | 5.56E-03 | 1.04 | 5.29E-01 | 0.90 | 1.39E-01 |
| Apoptosis Signaling | *CASP2* | A_24_P269398 | 835 | 0.93 | 1.44E-01 | 1.03 | 4.98E-01 | 0.95 | 3.49E-01 |
| Apoptosis Signaling | *BIK* | A_23_P404667 | 638 | 1.07 | 2.10E-01 | 0.97 | 5.09E-01 | 1.00 | 9.63E-01 |
| Apoptosis Signaling | *BCL2L2* | A_23_P418373 | 599 | 1.06 | 4.27E-01 | 1.02 | 7.72E-01 | 1.15 | 7.68E-02 |
| Apoptosis Signaling | *DFFB* | A_23_P44546 | 1677 | 1.04 | 4.96E-01 | 0.95 | 4.28E-01 | 0.99 | 8.51E-01 |
| Apoptosis Signaling | *DIABLO* | A_23_P47800 | 56616 | 1.08 | 5.79E-02 | 0.97 | 4.51E-01 | 1.01 | 9.04E-01 |
| Apoptosis Signaling | *CASP6* | A_23_P500799 | 839 | 0.93 | 1.92E-01 | 1.02 | 6.69E-01 | 0.92 | 1.49E-01 |
| Apoptosis Signaling | *BCL2L12* | A_23_P50477 | 83596 | 1.03 | 2.78E-01 | 0.98 | 4.93E-01 | 1.00 | 8.88E-01 |
| Apoptosis Signaling | *CASP8AP2* | A_23_P58898 | 9994 | 1.04 | 5.52E-01 | 0.96 | 4.77E-01 | 0.99 | 8.53E-01 |
| Apoptosis Signaling | *DAP3* | A_23_P63067 | 7818 | 1.08 | 7.48E-02 | 1.00 | 9.57E-01 | 1.03 | 5.14E-01 |
| Apoptosis Signaling | *FAS* | A_23_P63896 | 355 | 1.07 | 1.08E-01 | 1.03 | 5.14E-01 | 1.00 | 9.21E-01 |
| Apoptosis Signaling | *CAPNS1* | A_23_P67648 | 826 | 1.07 | 4.71E-02 | 0.99 | 8.14E-01 | 1.09 | 1.18E-02 |
| Apoptosis Signaling | *ENDOG* | A_23_P83266 | 2021 | 0.88 | 6.08E-02 | 1.04 | 5.53E-01 | 0.92 | 2.50E-01 |
| Apoptosis Signaling | *FADD* | A_23_P86917 | 8772 | 1.05 | 2.99E-01 | 1.07 | 1.20E-01 | 1.06 | 2.07E-01 |
| Apoptosis Signaling | *CASP14* | A_23_P90407 | 23581 | 0.92 | 1.53E-01 | 1.05 | 4.59E-01 | 1.05 | 4.62E-01 |
| Apoptosis Signaling | *CASP3* | A_23_P92410 | 836 | 1.05 | 3.14E-01 | 0.97 | 5.15E-01 | 1.04 | 4.45E-01 |
| Apoptosis Signaling | *DAP* | A_23_P92687 | 1611 | 0.98 | 6.53E-01 | 1.07 | 1.17E-01 | 1.12 | 6.50E-03 |
| Apoptosis Signaling | *TNFRSF10D* | A_23_P95417 | 8793 | 0.84 | 9.40E-02 | 1.05 | 6.38E-01 | 0.91 | 3.89E-01 |
| Apoptosis Signaling | *CASP9* | A_24_P111342 | 842 | 1.03 | 3.34E-01 | 0.97 | 3.82E-01 | 1.01 | 6.59E-01 |
| Apoptosis Signaling | *BIRC3* | A_23_P98350 | 330 | 1.06 | 3.58E-01 | 1.05 | 4.96E-01 | 1.08 | 2.50E-01 |
| Apoptosis Signaling | *BIRC2* | A_24_P115774 | 329 | 0.94 | 1.19E-01 | 1.05 | 1.99E-01 | 1.03 | 4.56E-01 |
| Apoptosis Signaling | *GAS2* | A_24_P179363 | 2620 | 1.08 | 1.92E-01 | 0.94 | 2.86E-01 | 1.05 | 3.98E-01 |
| Apoptosis Signaling | *CYCS* | A_24_P29665 | 54205 | 1.12 | 1.19E-01 | 0.93 | 2.86E-01 | 0.97 | 6.56E-01 |
| Apoptosis Signaling | *MCL1* | A_24_P336759 | 4170 | 1.01 | 8.75E-01 | 0.98 | 7.51E-01 | 1.01 | 9.26E-01 |
| Apoptosis Signaling | *HTRA2* | A_24_P336957 | 27429 | 0.92 | 7.15E-02 | 1.04 | 4.64E-01 | 0.97 | 5.39E-01 |
| Apoptosis Signaling | *CRADD* | A_32_P29806 | 8738 | 1.07 | 8.26E-02 | 1.01 | 7.67E-01 | 1.00 | 9.71E-01 |
| Phagocytosis Ag presentation | *PSMB9* | A_23_P111000 | 5698 | 1.11 | 2.33E-02 | 1.02 | 6.27E-01 | 1.08 | 1.13E-01 |
| Phagocytosis Ag presentation | *LAG3* | A_23_P116942 | 3902 | 1.15 | 1.02E-02 | 1.06 | 2.48E-01 | 1.14 | 1.62E-02 |
| Phagocytosis Ag presentation | *XBP1* | A_24_P100228 | 7494 | 1.13 | 9.55E-02 | 1.03 | 6.59E-01 | 1.20 | 1.27E-02 |
| Phagocytosis Ag presentation | *HLA-B* | A_23_P125107 | 3106 | 1.10 | 3.28E-02 | 0.97 | 5.54E-01 | 1.03 | 5.10E-01 |
| Phagocytosis Ag presentation | *RFX1* | A_23_P142037 | 5989 | 0.99 | 8.56E-01 | 1.05 | 4.20E-01 | 1.00 | 9.80E-01 |
| Phagocytosis Ag presentation | *PSMA1* | A_23_P150286 | 5682 | 1.11 | 1.71E-02 | 0.97 | 5.56E-01 | 1.06 | 1.95E-01 |
| Phagocytosis Ag presentation | *PSME1* | A_23_P151610 | 5720 | 1.10 | 2.75E-02 | 1.00 | 9.42E-01 | 1.06 | 1.59E-01 |
| Phagocytosis Ag presentation | *IFI30* | A_23_P153745 | 10437 | 1.15 | 2.48E-02 | 1.07 | 3.18E-01 | 1.09 | 1.85E-01 |
| Phagocytosis Ag presentation | *LILRB5* | A_23_P164784 | 10990 | 0.85 | 6.11E-02 | 1.03 | 7.38E-01 | 1.06 | 5.31E-01 |
| Phagocytosis Ag presentation | *RFXANK* | A_23_P165180 | 8625 | 1.02 | 3.13E-01 | 1.01 | 7.39E-01 | 1.02 | 4.96E-01 |
| Phagocytosis Ag presentation | *HLA-DQB2* | A_23_P19510 | 3120 | 0.98 | 6.60E-01 | 1.02 | 7.71E-01 | 0.99 | 8.92E-01 |
| Phagocytosis Ag presentation | *TAP2* | A_23_P368067 | 6891 | 0.88 | 2.21E-01 | 1.00 | 1.00E+00 | 1.09 | 4.30E-01 |
| Phagocytosis Ag presentation | *HLA-DPB1* | A_23_P258769 | 3115 | 1.12 | 1.16E-01 | 0.96 | 5.16E-01 | 1.04 | 6.41E-01 |
| Phagocytosis Ag presentation | *TAPBP* | A_23_P315336 | 6892 | 0.93 | 2.25E-01 | 1.05 | 4.15E-01 | 1.00 | 9.70E-01 |
| Phagocytosis Ag presentation | *NFX1* | A_23_P304543 | 4799 | 0.91 | 4.60E-02 | 1.03 | 5.79E-01 | 0.96 | 3.97E-01 |
| Phagocytosis Ag presentation | *HLA-DPA1* | A_23_P30913 | 3113 | 1.08 | 1.14E-01 | 0.98 | 7.33E-01 | 1.05 | 3.83E-01 |
| Phagocytosis Ag presentation | *HLA-DOA* | A_24_P332981 | 3111 | 1.15 | 6.31E-02 | 1.13 | 9.18E-02 | 1.09 | 2.80E-01 |
| Phagocytosis Ag presentation | *CIITA* | A_23_P33384 | 4261 | 1.09 | 1.34E-01 | 0.96 | 4.97E-01 | 1.02 | 7.99E-01 |
| Phagocytosis Ag presentation | *PRSS16* | A_23_P340131 | 10279 | 1.04 | 5.94E-01 | 0.96 | 5.84E-01 | 1.00 | 9.88E-01 |
| Phagocytosis Ag presentation | *CD1B* | A_23_P351844 | 910 | 1.01 | 8.12E-01 | 1.05 | 3.97E-01 | 1.04 | 4.86E-01 |
| Phagocytosis Ag presentation | *PSMB5* | A_23_P37191 | 5693 | 1.00 | 9.46E-01 | 1.04 | 1.87E-01 | 1.02 | 5.08E-01 |
| Phagocytosis Ag presentation | *CD1A* | A_23_P402670 | 909 | 1.08 | 4.40E-01 | 1.05 | 6.04E-01 | 1.20 | 1.33E-01 |
| Phagocytosis Ag presentation | *HLA-A* | A_24_P376483 | 3105 | 1.09 | 1.68E-01 | 1.02 | 7.97E-01 | 1.16 | 1.57E-02 |

| | | | | | | | | | |
|---|---|---|---|---|---|---|---|---|---|
| Phagocytosis Ag presentation | *HLA-DMA* | A_23_P42306 | 3108 | 1.12 | 3.46E-02 | 0.97 | 5.99E-01 | 1.02 | 7.02E-01 |
| Phagocytosis Ag presentation | *CTSS* | A_24_P242646 | 1520 | 1.07 | 4.63E-01 | 1.13 | 2.31E-01 | 1.24 | 2.02E-02 |
| Phagocytosis Ag presentation | *CD1C* | A_23_P51767 | 911 | 1.10 | 1.37E-01 | 1.07 | 2.67E-01 | 0.98 | 7.81E-01 |
| Phagocytosis Ag presentation | *TAP1* | A_23_P59005 | 6890 | 1.05 | 1.68E-01 | 1.03 | 3.74E-01 | 1.00 | 9.50E-01 |
| Phagocytosis Ag presentation | *PSME2* | A_23_P65427 | 5721 | 1.14 | 9.93E-03 | 1.00 | 9.50E-01 | 1.07 | 1.94E-01 |
| Phagocytosis Ag presentation | *CD74* | A_23_P70095 | 972 | 1.09 | 1.73E-01 | 0.94 | 3.01E-01 | 1.03 | 6.75E-01 |
| Phagocytosis Ag presentation | *CD1D* | A_23_P74575 | 912 | 1.14 | 7.72E-02 | 1.14 | 8.81E-02 | 1.07 | 4.02E-01 |
| Phagocytosis Ag presentation | *CD209* | A_24_P186539 | 30835 | 0.99 | 9.01E-01 | 1.05 | 3.40E-01 | 0.96 | 4.92E-01 |
| Phagocytosis Ag presentation | *HLA-DQA1* | A_24_P196827 | 3117 | 0.94 | 7.17E-01 | 1.05 | 7.73E-01 | 1.15 | 4.56E-01 |
| Phagocytosis Ag presentation | *RFX4* | A_24_P224158 | 5992 | 0.85 | 1.19E-01 | 1.09 | 4.37E-01 | 0.91 | 3.91E-01 |
| Phagocytosis Ag presentation | *HLA-C* | A_24_P298409 | 3107 | 1.08 | 4.45E-02 | 0.98 | 5.18E-01 | 1.01 | 7.56E-01 |
| Phagocytosis Ag presentation | *HLA-DQA2* | A_24_P852756 | 3118 | 1.04 | 6.72E-01 | 0.95 | 6.04E-01 | 0.92 | 4.18E-01 |
| Phagocytosis Ag presentation | *HLA-DMB* | A_32_P351968 | 3109 | 1.13 | 3.75E-02 | 0.99 | 8.75E-01 | 1.05 | 4.32E-01 |
| Phagocytosis Ag presentation | *HLA-DRA* | A_32_P87697 | 3122 | 1.09 | 1.98E-01 | 0.97 | 6.53E-01 | 1.05 | 4.74E-01 |
| PI3K AKT Signaling | *ILK* | A_23_P105066 | 3611 | 1.06 | 1.94E-01 | 1.00 | 9.80E-01 | 1.10 | 4.93E-02 |
| PI3K AKT Signaling | *EIF4E* | A_32_P203300 | 1977 | 1.06 | 2.34E-01 | 0.98 | 6.16E-01 | 1.02 | 7.69E-01 |
| PI3K AKT Signaling | *CDC37* | A_23_P130531 | 11140 | 1.09 | 9.86E-03 | 1.01 | 8.57E-01 | 1.10 | 3.63E-03 |
| PI3K AKT Signaling | *RHEB* | A_23_P134247 | 6009 | 1.03 | 3.00E-01 | 0.99 | 8.60E-01 | 1.04 | 1.68E-01 |
| PI3K AKT Signaling | *MDM2* | A_24_P925664 | 4193 | 1.04 | 6.75E-01 | 1.05 | 6.02E-01 | 1.12 | 2.22E-01 |
| PI3K AKT Signaling | *RPS6KB1* | A_24_P497226 | 6198 | 1.09 | 4.72E-02 | 0.97 | 5.59E-01 | 1.04 | 3.47E-01 |
| PI3K AKT Signaling | *PIK3R2* | A_23_P142361 | 5296 | 0.89 | 2.38E-02 | 1.01 | 8.13E-01 | 0.96 | 4.60E-01 |
| PI3K AKT Signaling | *PIK3R1* | A_23_P144980 | 5295 | 1.02 | 6.95E-01 | 0.91 | 1.36E-01 | 1.01 | 9.24E-01 |
| PI3K AKT Signaling | *HSP90AB1* | A_24_P170295 | 3326 | 1.05 | 3.30E-01 | 0.95 | 3.25E-01 | 1.05 | 4.05E-01 |
| PI3K AKT Signaling | *THEM4* | A_24_P928510 | 117145 | 1.19 | 1.52E-02 | 1.02 | 7.40E-01 | 0.99 | 8.44E-01 |
| PI3K AKT Signaling | *AKT3* | A_24_P45481 | 10000 | 1.10 | 1.91E-01 | 0.93 | 3.38E-01 | 1.08 | 3.43E-01 |
| PI3K AKT Signaling | *HSP90AA1* | A_32_P199252 | 3320 | 1.05 | 3.21E-01 | 0.97 | 6.19E-01 | 1.10 | 6.80E-02 |
| PI3K AKT Signaling | *CDKN1B* | A_24_P81841 | 1027 | 1.02 | 6.68E-01 | 0.95 | 3.66E-01 | 0.95 | 2.85E-01 |
| PI3K AKT Signaling | *AKT2* | A_23_P373475 | 208 | 1.04 | 4.15E-01 | 1.04 | 4.51E-01 | 1.04 | 4.52E-01 |
| PI3K AKT Signaling | *LIMS1* | A_23_P210358 | 3987 | 1.03 | 6.88E-01 | 0.98 | 6.94E-01 | 1.06 | 3.50E-01 |
| PI3K AKT Signaling | *PIK3R3* | A_23_P22970 | 8503 | 1.03 | 7.69E-01 | 1.04 | 6.14E-01 | 1.00 | 9.82E-01 |
| PI3K AKT Signaling | *MAP3K8* | A_23_P23947 | 1326 | 1.01 | 9.23E-01 | 0.96 | 5.30E-01 | 1.07 | 2.40E-01 |
| PI3K AKT Signaling | *RPS6KB2* | A_23_P24318 | 6199 | 0.97 | 2.72E-01 | 1.07 | 2.67E-02 | 1.06 | 7.75E-02 |
| PI3K AKT Signaling | *PIK3CD* | A_24_P71244 | 5293 | 1.03 | 5.02E-01 | 1.00 | 9.59E-01 | 1.02 | 6.39E-01 |
| PI3K AKT Signaling | *CTNNB1* | A_23_P29499 | 1499 | 0.88 | 2.05E-01 | 1.02 | 8.09E-01 | 0.95 | 6.20E-01 |
| PI3K AKT Signaling | *AKT1* | A_23_P2960 | 207 | 1.05 | 3.14E-01 | 1.01 | 7.95E-01 | 1.06 | 3.04E-01 |
| PI3K AKT Signaling | *PIK3CB* | A_23_P346969 | 5291 | 1.04 | 4.86E-01 | 1.03 | 5.18E-01 | 1.03 | 6.29E-01 |
| PI3K AKT Signaling | *INPPL1* | A_23_P36322 | 3636 | 1.04 | 1.45E-01 | 1.03 | 3.21E-01 | 1.04 | 1.14E-01 |
| PI3K AKT Signaling | *CDKN1A* | A_23_P59210 | 1026 | 0.91 | 6.73E-02 | 1.04 | 3.89E-01 | 1.00 | 9.35E-01 |
| PI3K AKT Signaling | *INPP5D* | A_24_P912074 | 3635 | 0.94 | 3.74E-01 | 0.95 | 4.38E-01 | 0.95 | 4.98E-01 |
| PI3K AKT Signaling | *TSC2* | A_23_P66110 | 7249 | 0.92 | 2.05E-01 | 0.98 | 7.53E-01 | 0.94 | 3.63E-01 |
| PI3K AKT Signaling | *PDPK1* | A_24_P222599 | 5170 | 1.13 | 9.61E-02 | 0.93 | 3.05E-01 | 0.96 | 6.11E-01 |
| PI3K AKT Signaling | *PIK3R5* | A_23_P66543 | 23533 | 1.04 | 3.59E-01 | 0.96 | 4.44E-01 | 1.08 | 9.00E-02 |
| PI3K AKT Signaling | *NOS3* | A_23_P70849 | 4846 | 0.89 | 5.67E-02 | 1.02 | 7.18E-01 | 0.96 | 5.80E-01 |
| PI3K AKT Signaling | *PIK3CA* | A_23_P92057 | 5290 | 1.00 | 9.33E-01 | 0.96 | 4.62E-01 | 0.99 | 8.65E-01 |
| PI3K AKT Signaling | *PTEN* | A_24_P913115 | 5728 | 1.05 | 2.92E-01 | 1.01 | 7.66E-01 | 0.99 | 8.33E-01 |
| PI3K AKT Signaling | *CCND1* | A_24_P193011 | 595 | 0.83 | 2.61E-02 | 1.02 | 7.72E-01 | 0.90 | 1.92E-01 |
| PI3K AKT Signaling | *TSC1* | A_24_P329635 | 7248 | 1.02 | 6.50E-01 | 0.96 | 4.49E-01 | 1.05 | 2.96E-01 |
| PI3K AKT Signaling | *GAB1* | A_24_P936319 | 2549 | 1.01 | 9.38E-01 | 1.02 | 7.78E-01 | 1.00 | 9.75E-01 |
| Innate pathogen detection | *TLR1* | A_23_P10873 | 7096 | 0.99 | 8.60E-01 | 1.11 | 2.61E-02 | 1.04 | 4.01E-01 |
| Innate pathogen detection | *TLR9* | A_23_P132654 | 54106 | 1.06 | 2.74E-01 | 0.98 | 7.00E-01 | 0.94 | 2.46E-01 |
| Innate pathogen detection | *IRAK4* | A_24_P158903 | 51135 | 1.08 | 5.45E-02 | 1.00 | 9.36E-01 | 1.01 | 8.93E-01 |
| Innate pathogen detection | *IRAK3* | A_32_P83256 | 11213 | 1.07 | 2.83E-01 | 1.14 | 7.47E-02 | 1.10 | 1.17E-01 |
| Innate pathogen detection | *TIRAP* | A_23_P202905 | 114609 | 1.16 | 5.02E-02 | 1.13 | 1.06E-01 | 1.23 | 1.14E-02 |
| Innate pathogen detection | *OAS2* | A_24_P343929 | 4939 | 1.11 | 1.43E-01 | 1.02 | 7.65E-01 | 1.06 | 4.44E-01 |
| Innate pathogen detection | *DDX58* | A_23_P20814 | 23586 | 1.08 | 1.89E-01 | 1.01 | 8.68E-01 | 0.97 | 6.33E-01 |
| Innate pathogen detection | *PGLYRP1* | A_23_P208747 | 8993 | 1.22 | 4.03E-02 | 1.16 | 9.57E-02 | 1.10 | 3.39E-01 |
| Innate pathogen detection | *TLR6* | A_23_P256561 | 10333 | 1.04 | 4.76E-01 | 1.07 | 2.44E-01 | 0.97 | 5.75E-01 |
| Innate pathogen detection | *CD180* | A_23_P257815 | 4064 | 1.20 | 1.54E-01 | 1.15 | 2.44E-01 | 1.18 | 2.23E-01 |
| Innate pathogen detection | *HSP90B1* | A_23_P2601 | 7184 | 0.80 | 1.67E-03 | 1.08 | 2.85E-01 | 0.99 | 9.28E-01 |
| Innate pathogen detection | *PYCARD* | A_23_P26629 | 29108 | 1.16 | 3.96E-03 | 1.03 | 6.00E-01 | 1.07 | 2.13E-01 |
| Innate pathogen detection | *CARD8* | A_24_P14260 | 22900 | 1.14 | 2.95E-02 | 1.08 | 2.32E-01 | 1.12 | 7.31E-02 |
| Innate pathogen detection | *TLR3* | A_23_P29922 | 7098 | 1.08 | 3.86E-01 | 1.05 | 5.74E-01 | 0.97 | 7.54E-01 |
| Innate pathogen detection | *TLR10* | A_23_P33420 | 81793 | 1.07 | 4.51E-01 | 1.06 | 5.17E-01 | 1.03 | 7.80E-01 |
| Innate pathogen detection | *MYD88* | A_23_P362659 | 4615 | 1.04 | 2.45E-01 | 1.05 | 1.53E-01 | 1.05 | 1.44E-01 |
| Innate pathogen detection | *TICAM1* | A_23_P376096 | 148022 | 0.93 | 1.34E-01 | 0.99 | 7.38E-01 | 0.93 | 1.16E-01 |
| Innate pathogen detection | *CARD6* | A_23_P41854 | 84674 | 1.10 | 1.68E-01 | 1.11 | 1.45E-01 | 1.11 | 1.34E-01 |
| Innate pathogen detection | *CASP5* | A_23_P47304 | 838 | 1.13 | 4.62E-03 | 1.05 | 2.51E-01 | 1.08 | 7.74E-02 |
| Innate pathogen detection | *TLR4* | A_23_P60306 | 7099 | 1.06 | 4.09E-01 | 1.04 | 6.03E-01 | 0.99 | 9.05E-01 |
| Innate pathogen detection | *TLR7* | A_23_P62437 | 51284 | 1.07 | 4.47E-01 | 1.08 | 3.83E-01 | 1.12 | 2.18E-01 |
| Innate pathogen detection | *OAS1* | A_23_P64828 | 4938 | 1.06 | 4.28E-01 | 1.05 | 5.61E-01 | 1.02 | 8.25E-01 |
| Innate pathogen detection | *IFIH1* | A_23_P68155 | 64135 | 1.05 | 3.50E-01 | 1.00 | 9.92E-01 | 1.04 | 4.91E-01 |
| Innate pathogen detection | *IRAK1* | A_23_P73780 | 3654 | 0.88 | 3.47E-02 | 1.00 | 9.47E-01 | 0.95 | 4.72E-01 |
| Innate pathogen detection | *TLR8* | A_23_P73837 | 51311 | 1.16 | 7.41E-02 | 1.12 | 2.25E-01 | 1.04 | 6.28E-01 |
| Innate pathogen detection | *TOLLIP* | A_24_P287189 | 54472 | 0.99 | 9.32E-01 | 1.11 | 3.17E-01 | 1.15 | 2.16E-01 |
| Innate pathogen detection | *IRAK2* | A_23_P80635 | 3656 | 0.91 | 1.43E-01 | 1.02 | 8.01E-01 | 0.94 | 3.24E-01 |
| Innate pathogen detection | *TLR5* | A_23_P85903 | 7100 | 1.17 | 2.00E-02 | 1.19 | 9.06E-03 | 1.12 | 9.89E-02 |
| Innate pathogen detection | *PRKRA* | A_23_P91019 | 8575 | 1.00 | 9.14E-01 | 0.97 | 4.93E-01 | 1.05 | 2.31E-01 |
| Innate pathogen detection | *TLR2* | A_23_P92499 | 7097 | 1.10 | 1.47E-01 | 1.05 | 5.64E-01 | 0.95 | 4.83E-01 |
| Innate pathogen detection | *LY96* | A_23_P94230 | 23643 | 1.19 | 5.15E-03 | 1.06 | 3.60E-01 | 1.08 | 2.29E-01 |
| Innate pathogen detection | *PGLYRP3* | A_23_P96899 | 114771 | 0.86 | 1.00E-01 | 1.00 | 9.83E-01 | 0.94 | 5.42E-01 |
| Innate pathogen detection | *SARM1* | A_24_P267293 | 23098 | 1.06 | 1.79E-01 | 0.91 | 2.80E-02 | 0.99 | 7.40E-01 |
| Innate pathogen detection | *CD14* | A_24_P283189 | 929 | 0.99 | 9.00E-01 | 1.08 | 1.66E-01 | 1.03 | 5.55E-01 |
| G Protein Coupled Receptor Signaling | *ADORA2A* | A_23_P109436 | 135 | 0.85 | 3.13E-02 | 1.08 | 3.13E-01 | 1.00 | 9.90E-01 |

| | | | | | | | | | |
|---|---|---|---|---|---|---|---|---|---|
| G Protein Coupled Receptor Signaling | *PDE3B* | A_23_P116114 | 5140 | 1.08 | 6.29E-02 | 0.96 | 3.07E-01 | 0.96 | 3.76E-01 |
| G Protein Coupled Receptor Signaling | *PDE4C* | A_24_P139120 | 5143 | 0.88 | 7.01E-02 | 1.05 | 4.55E-01 | 0.96 | 6.12E-01 |
| G Protein Coupled Receptor Signaling | *PDE4D* | A_24_P944519 | 5144 | 1.00 | 9.83E-01 | 0.85 | 8.10E-02 | 0.97 | 7.69E-01 |
| G Protein Coupled Receptor Signaling | *ADORA3* | A_23_P126540 | 140 | 0.83 | 8.10E-02 | 1.04 | 7.35E-01 | 0.99 | 9.64E-01 |
| G Protein Coupled Receptor Signaling | *ADRA2A* | A_23_P138706 | 150 | 1.00 | 9.81E-01 | 0.93 | 2.59E-01 | 1.07 | 3.24E-01 |
| G Protein Coupled Receptor Signaling | *PDE1B* | A_23_P139585 | 5153 | 1.01 | 8.69E-01 | 1.04 | 3.86E-01 | 0.97 | 4.25E-01 |
| G Protein Coupled Receptor Signaling | *ADRB2* | A_23_P145024 | 154 | 1.13 | 1.11E-01 | 1.08 | 2.87E-01 | 1.15 | 7.08E-02 |
| G Protein Coupled Receptor Signaling | *CREBBP* | A_24_P322025 | 1387 | 1.03 | 5.49E-01 | 0.96 | 3.88E-01 | 0.99 | 8.37E-01 |
| G Protein Coupled Receptor Signaling | *PTK2B* | A_23_P168836 | 2185 | 1.03 | 3.51E-01 | 1.06 | 5.53E-02 | 1.08 | 1.67E-02 |
| G Protein Coupled Receptor Signaling | *CREM* | A_24_P171075 | 1390 | 0.95 | 5.09E-01 | 0.92 | 3.17E-01 | 1.06 | 4.95E-01 |
| G Protein Coupled Receptor Signaling | *ADCY1* | A_23_P21002 | 107 | 0.87 | 1.57E-01 | 1.14 | 1.77E-01 | 0.96 | 7.09E-01 |
| G Protein Coupled Receptor Signaling | *PRKAR2A* | A_24_P943335 | 5576 | 1.04 | 5.34E-01 | 0.98 | 7.11E-01 | 1.07 | 3.03E-01 |
| G Protein Coupled Receptor Signaling | *ADRA2C* | A_23_P256158 | 152 | 1.08 | 2.66E-01 | 1.10 | 1.20E-01 | 1.05 | 4.85E-01 |
| G Protein Coupled Receptor Signaling | *ADCY2* | A_23_P33539 | 108 | 0.83 | 1.23E-01 | 1.14 | 2.85E-01 | 1.08 | 5.55E-01 |
| G Protein Coupled Receptor Signaling | *PRKACB* | A_24_P62708 | 5567 | 1.09 | 1.32E-01 | 0.95 | 3.59E-01 | 1.03 | 6.25E-01 |
| G Protein Coupled Receptor Signaling | *ADCY4* | A_23_P381261 | 196883 | 1.04 | 3.87E-01 | 1.03 | 5.40E-01 | 1.02 | 6.78E-01 |
| G Protein Coupled Receptor Signaling | *PDE2A* | A_23_P87379 | 5138 | 0.99 | 7.84E-01 | 1.00 | 9.66E-01 | 1.07 | 1.85E-01 |
| G Protein Coupled Receptor Signaling | *PRKAR2B* | A_23_P42975 | 5577 | 1.02 | 7.53E-01 | 1.00 | 9.61E-01 | 1.15 | 6.36E-02 |
| G Protein Coupled Receptor Signaling | *PRKAR1A* | A_24_P356592 | 5573 | 1.00 | 9.96E-01 | 0.97 | 4.53E-01 | 1.05 | 2.85E-01 |
| G Protein Coupled Receptor Signaling | *ADORA2B* | A_23_P55477 | 136 | 1.03 | 6.65E-01 | 1.03 | 6.64E-01 | 1.19 | 1.16E-02 |
| G Protein Coupled Receptor Signaling | *PLCB1* | A_24_P941643 | 23236 | 0.99 | 9.02E-01 | 1.06 | 2.33E-01 | 1.01 | 9.10E-01 |
| G Protein Coupled Receptor Signaling | *PRKACG* | A_23_P71926 | 5568 | 0.98 | 7.55E-01 | 1.08 | 2.63E-01 | 0.96 | 6.12E-01 |
| G Protein Coupled Receptor Signaling | *PDE4B* | A_24_P325333 | 5142 | 0.99 | 8.99E-01 | 0.87 | 8.16E-02 | 0.97 | 7.17E-01 |
| G Protein Coupled Receptor Signaling | *ADORA1* | A_23_P74299 | 134 | 0.78 | 1.29E-02 | 0.94 | 5.45E-01 | 0.89 | 2.80E-01 |
| G Protein Coupled Receptor Signaling | *HRH2* | A_23_P7535 | 3274 | 0.86 | 5.98E-02 | 1.04 | 5.93E-01 | 0.93 | 3.79E-01 |
| G Protein Coupled Receptor Signaling | *CREB1* | A_24_P932208 | 1385 | 0.93 | 2.72E-01 | 1.07 | 2.94E-01 | 1.12 | 1.11E-01 |
| G Protein Coupled Receptor Signaling | *RGS1* | A_23_P97141 | 5996 | 1.03 | 8.30E-01 | 0.81 | 1.18E-01 | 0.91 | 5.44E-01 |
| G Protein Coupled Receptor Signaling | *PDE1A* | A_24_P208436 | 5136 | 0.91 | 2.02E-01 | 1.06 | 4.48E-01 | 0.96 | 6.05E-01 |
| G Protein Coupled Receptor Signaling | *PLCB2* | A_24_P936779 | 5330 | 1.02 | 6.40E-01 | 1.01 | 7.35E-01 | 0.98 | 6.19E-01 |
| G Protein Coupled Receptor Signaling | *PDE4A* | A_24_P322474 | 5141 | 1.08 | 1.49E-01 | 0.95 | 4.29E-01 | 1.07 | 2.22E-01 |
| G Protein Coupled Receptor Signaling | *ADRBK1* | A_24_P326635 | 156 | 1.08 | 8.46E-02 | 1.00 | 9.97E-01 | 1.06 | 2.23E-01 |
| G Protein Coupled Receptor Signaling | *PRKACA* | A_24_P399630 | 5566 | 0.96 | 3.84E-01 | 1.01 | 7.88E-01 | 0.97 | 4.11E-01 |
| G Protein Coupled Receptor Signaling | *SYNGAP1* | A_24_P9346 | 8831 | 1.09 | 1.36E-01 | 1.07 | 2.52E-01 | 1.12 | 6.15E-02 |
| NF kB signaling | *RELA* | A_24_P937256 | 5970 | 0.93 | 3.24E-01 | 1.02 | 7.55E-01 | 0.96 | 6.24E-01 |
| NF kB signaling | *NFKBIA* | A_23_P106002 | 4792 | 1.01 | 8.94E-01 | 0.84 | 2.88E-02 | 0.95 | 5.51E-01 |
| NF kB signaling | *UBE2V1* | A_23_P11461 | 7335 | 0.97 | 5.17E-01 | 1.00 | 9.22E-01 | 0.94 | 2.29E-01 |
| NF kB signaling | *BCL10* | A_24_P19983 | 8915 | 0.92 | 1.06E-01 | 0.96 | 3.90E-01 | 1.02 | 7.72E-01 |
| NF kB signaling | *UBE2N* | A_23_P116829 | 7334 | 1.04 | 4.36E-01 | 0.99 | 7.93E-01 | 0.99 | 8.70E-01 |
| NF kB signaling | *MEFV* | A_23_P140967 | 4210 | 1.01 | 8.39E-01 | 1.00 | 9.54E-01 | 0.95 | 1.97E-01 |
| NF kB signaling | *EIF2AK2* | A_23_P142750 | 5610 | 1.07 | 2.46E-01 | 1.01 | 8.16E-01 | 1.03 | 5.66E-01 |
| NF kB signaling | *CSNK2A2* | A_23_P14915 | 1459 | 1.11 | 3.50E-02 | 0.98 | 6.12E-01 | 1.01 | 8.34E-01 |
| NF kB signaling | *IKBKG* | A_23_P159920 | 8517 | 1.00 | 9.76E-01 | 0.98 | 6.88E-01 | 0.99 | 8.68E-01 |
| NF kB signaling | *NFKB2* | A_23_P202156 | 4791 | 0.94 | 9.89E-02 | 0.97 | 3.44E-01 | 0.96 | 3.26E-01 |
| NF kB signaling | *MAP3K3* | A_23_P207138 | 4215 | 1.09 | 2.64E-02 | 0.98 | 6.03E-01 | 1.00 | 9.43E-01 |
| NF kB signaling | *MAP3K14* | A_23_P207319 | 9020 | 1.07 | 8.59E-02 | 1.01 | 7.65E-01 | 1.03 | 4.48E-01 |
| NF kB signaling | *CARD14* | A_23_P207879 | 79092 | 0.84 | 4.78E-03 | 1.01 | 8.59E-01 | 0.92 | 1.75E-01 |
| NF kB signaling | *IKBKB* | A_24_P155058 | 3551 | 0.94 | 2.95E-01 | 0.96 | 4.24E-01 | 0.96 | 4.84E-01 |
| NF kB signaling | *NFRKB* | A_23_P24485 | 4798 | 0.97 | 6.63E-01 | 1.09 | 1.73E-01 | 0.99 | 8.48E-01 |
| NF kB signaling | *NFKB1* | A_23_P30024 | 4790 | 1.03 | 2.94E-01 | 0.98 | 5.64E-01 | 1.03 | 4.26E-01 |
| NF kB signaling | *NFKBIE* | A_23_P30655 | 4794 | 1.03 | 5.65E-01 | 0.90 | 8.61E-02 | 1.02 | 7.32E-01 |
| NF kB signaling | *NFKBIB* | A_23_P79086 | 4793 | 0.89 | 9.64E-02 | 1.05 | 5.15E-01 | 0.93 | 3.39E-01 |
| NF kB signaling | *BTRC* | A_23_P35427 | 8945 | 0.88 | 4.81E-02 | 1.01 | 9.04E-01 | 0.94 | 3.56E-01 |
| NF kB signaling | *RIPK1* | A_23_P370005 | 8737 | 1.07 | 1.32E-01 | 0.94 | 1.59E-01 | 1.01 | 7.68E-01 |
| NF kB signaling | *CARD10* | A_23_P434890 | 29775 | 1.04 | 4.88E-01 | 1.00 | 9.45E-01 | 1.04 | 5.05E-01 |
| NF kB signaling | *TBK1* | A_23_P44768 | 29110 | 1.02 | 4.32E-01 | 1.02 | 5.93E-01 | 1.01 | 6.32E-01 |
| NF kB signaling | *BCL3* | A_23_P4662 | 602 | 1.11 | 1.14E-01 | 0.93 | 3.14E-01 | 0.99 | 8.72E-01 |
| NF kB signaling | *CHUK* | A_23_P46748 | 1147 | 0.98 | 6.63E-01 | 0.97 | 3.91E-01 | 0.94 | 1.14E-01 |
| NF kB signaling | *CARD9* | A_23_P500433 | 64170 | 1.10 | 1.63E-01 | 1.07 | 4.03E-01 | 1.14 | 5.91E-02 |
| NF kB signaling | *CSNK2A1* | A_24_P936444 | 1457 | 0.95 | 1.30E-01 | 1.07 | 9.37E-02 | 0.94 | 1.14E-01 |
| NF kB signaling | *RELB* | A_23_P55706 | 5971 | 1.06 | 2.38E-01 | 1.01 | 8.56E-01 | 1.12 | 2.30E-02 |
| NF kB signaling | *REL* | A_23_P56938 | 5966 | 0.99 | 7.49E-01 | 0.95 | 2.77E-01 | 1.06 | 1.91E-01 |
| NF kB signaling | *BCL6* | A_23_P57856 | 604 | 1.05 | 5.28E-01 | 1.05 | 5.69E-01 | 0.97 | 7.05E-01 |
| NF kB signaling | *CARD11* | A_24_P945262 | 84433 | 1.07 | 2.99E-01 | 0.92 | 1.85E-01 | 1.08 | 2.22E-01 |
| NF kB signaling | *MALT1* | A_32_P76576 | 10892 | 1.07 | 3.08E-01 | 0.98 | 7.85E-01 | 1.08 | 2.57E-01 |
| NF kB signaling | *CSNK2B* | A_24_P97931 | 1460 | 1.06 | 5.37E-02 | 0.98 | 4.59E-01 | 1.00 | 9.61E-01 |
| Eicosanoid Signaling | *ALOX5* | A_23_P104464 | 240 | 1.06 | 3.36E-01 | 1.01 | 9.23E-01 | 1.02 | 7.75E-01 |
| Eicosanoid Signaling | *MGST2* | A_23_P110167 | 4258 | 1.17 | 7.16E-03 | 1.07 | 2.21E-01 | 1.12 | 4.65E-02 |
| Eicosanoid Signaling | *DPEP2* | A_23_P118025 | 64174 | 1.03 | 5.53E-01 | 0.98 | 6.97E-01 | 0.99 | 8.85E-01 |
| Eicosanoid Signaling | *GGT1* | A_24_P178175 | 2678 | 0.89 | 9.66E-02 | 1.05 | 4.91E-01 | 0.97 | 6.45E-01 |
| Eicosanoid Signaling | *DPEP3* | A_23_P129413 | 64180 | 0.91 | 3.69E-02 | 0.99 | 7.57E-01 | 0.96 | 3.88E-01 |
| Eicosanoid Signaling | *AKR1C3* | A_23_P138541 | 8644 | 1.02 | 8.50E-01 | 0.94 | 5.10E-01 | 1.08 | 4.19E-01 |
| Eicosanoid Signaling | *PTGDS* | A_23_P146554 | 5730 | 0.95 | 3.46E-01 | 1.00 | 9.35E-01 | 0.98 | 7.12E-01 |
| Eicosanoid Signaling | *PTGER4* | A_23_P148047 | 5734 | 1.07 | 2.18E-01 | 0.98 | 7.30E-01 | 1.02 | 7.37E-01 |
| Eicosanoid Signaling | *PTGER2* | A_23_P151710 | 5732 | 1.08 | 5.63E-02 | 1.06 | 2.13E-01 | 1.08 | 6.73E-02 |
| Eicosanoid Signaling | *DPEP1* | A_23_P152262 | 1800 | 0.86 | 7.33E-02 | 1.11 | 2.03E-01 | 0.95 | 5.59E-01 |
| Eicosanoid Signaling | *ALOX12* | A_23_P152906 | 239 | 1.05 | 5.63E-01 | 1.03 | 7.24E-01 | 1.18 | 6.80E-02 |
| Eicosanoid Signaling | *PTGS1* | A_24_P64167 | 5742 | 1.08 | 3.66E-01 | 1.05 | 5.75E-01 | 1.17 | 6.75E-02 |
| Eicosanoid Signaling | *CYSLTR1* | A_23_P22660 | 10800 | 1.03 | 2.72E-01 | 1.01 | 7.36E-01 | 0.95 | 1.17E-01 |
| Eicosanoid Signaling | *ALOX5AP* | A_24_P347378 | 241 | 1.07 | 1.41E-01 | 0.98 | 7.37E-01 | 1.01 | 7.99E-01 |
| Eicosanoid Signaling | *PLA2G2D* | A_23_P300100 | 26279 | 0.85 | 7.32E-02 | 0.90 | 2.23E-01 | 0.94 | 4.93E-01 |
| Eicosanoid Signaling | *CYSLTR2* | A_23_P319466 | 57105 | 0.80 | 1.10E-02 | 1.03 | 7.55E-01 | 0.94 | 5.36E-01 |
| Eicosanoid Signaling | *PLA2G2A* | A_23_P321949 | 5320 | 0.88 | 1.58E-01 | 1.07 | 4.10E-01 | 0.97 | 7.34E-01 |
| Eicosanoid Signaling | *PTGIR* | A_24_P322426 | 5739 | 0.91 | 9.78E-02 | 1.06 | 2.96E-01 | 0.98 | 6.97E-01 |

| | | | | | | | | | |
|---|---|---|---|---|---|---|---|---|---|
| Eicosanoid Signaling | *TBXA2R* | A_23_P355471 | 6915 | 0.89 | 5.06E-02 | 1.01 | 8.24E-01 | 0.93 | 2.37E-01 |
| Eicosanoid Signaling | *LTA4H* | A_23_P388670 | 4048 | 1.10 | 1.19E-01 | 1.01 | 8.87E-01 | 1.08 | 2.26E-01 |
| Eicosanoid Signaling | *PTGDR* | A_23_P393777 | 5729 | 0.96 | 4.44E-01 | 1.02 | 7.29E-01 | 0.94 | 2.66E-01 |
| Eicosanoid Signaling | *MGST3* | A_23_P51548 | 4259 | 1.14 | 4.20E-03 | 1.00 | 9.57E-01 | 1.10 | 4.04E-02 |
| Eicosanoid Signaling | *ALOX15* | A_24_P931250 | 246 | 0.92 | 3.58E-01 | 0.99 | 9.08E-01 | 0.95 | 5.54E-01 |
| Eicosanoid Signaling | *ALOX15B* | A_23_P60627 | 247 | 0.95 | 2.49E-01 | 1.01 | 7.67E-01 | 1.00 | 9.99E-01 |
| Eicosanoid Signaling | *ALOX12B* | A_23_P83634 | 242 | 0.93 | 1.56E-01 | 1.03 | 5.54E-01 | 0.98 | 7.19E-01 |
| Eicosanoid Signaling | *PTGES2* | A_24_P106953 | 80142 | 1.07 | 1.20E-01 | 1.02 | 6.69E-01 | 1.13 | 5.47E-03 |
| Eicosanoid Signaling | *GPR44* | A_24_P115932 | 11251 | 1.16 | 5.81E-02 | 1.06 | 4.87E-01 | 1.12 | 1.41E-01 |
| Eicosanoid Signaling | *PTGS2* | A_24_P77008 | 5743 | 0.88 | 3.37E-01 | 0.80 | 1.24E-01 | 0.85 | 2.27E-01 |
| Eicosanoid Signaling | *PTGES3* | A_32_P114896 | 10728 | 0.89 | 4.48E-02 | 1.05 | 4.52E-01 | 1.04 | 5.05E-01 |
| Eicosanoid Signaling | *TBXAS1* | A_32_P212886 | 6916 | 0.85 | 7.02E-02 | 1.06 | 5.43E-01 | 0.92 | 3.59E-01 |
| Eicosanoid Signaling | *LTC4S* | A_24_P397294 | 4056 | 0.98 | 6.92E-01 | 0.97 | 5.35E-01 | 0.95 | 2.38E-01 |
| Eicosanoid Signaling | *PTGER3* | A_24_P945365 | 5733 | 0.90 | 7.66E-02 | 1.00 | 9.88E-01 | 0.97 | 6.33E-01 |
| TNF Superfamily Signaling | *PTX3* | A_23_P121064 | 5806 | 0.94 | 4.02E-01 | 0.83 | 2.00E-02 | 0.94 | 4.27E-01 |
| TNF Superfamily Signaling | *TNFSF4* | A_23_P126836 | 7292 | 0.99 | 8.85E-01 | 1.10 | 2.57E-01 | 1.17 | 8.88E-02 |
| TNF Superfamily Signaling | *TNFRSF25* | A_23_P126844 | 8718 | 1.11 | 9.60E-02 | 0.97 | 5.66E-01 | 1.07 | 2.55E-01 |
| TNF Superfamily Signaling | *TNFRSF14* | A_23_P126908 | 8764 | 1.09 | 5.06E-02 | 1.02 | 6.09E-01 | 1.08 | 1.21E-01 |
| TNF Superfamily Signaling | *TNFRSF1A* | A_23_P139722 | 7132 | 1.12 | 1.13E-01 | 1.17 | 4.80E-02 | 1.13 | 1.02E-01 |
| TNF Superfamily Signaling | *TNFSF13B* | A_23_P14174 | 10673 | 1.13 | 4.33E-02 | 1.03 | 5.99E-01 | 1.07 | 2.58E-01 |
| TNF Superfamily Signaling | *LTA* | A_23_P156683 | 4049 | 0.84 | 4.51E-02 | 1.13 | 1.80E-01 | 0.93 | 4.22E-01 |
| TNF Superfamily Signaling | *TNFAIP6* | A_23_P165624 | 7130 | 1.11 | 3.88E-01 | 1.05 | 6.68E-01 | 1.13 | 3.12E-01 |
| TNF Superfamily Signaling | *TNFSF8* | A_23_P169257 | 944 | 0.97 | 5.00E-01 | 1.01 | 8.26E-01 | 0.95 | 2.46E-01 |
| TNF Superfamily Signaling | *TRAF2* | A_23_P169331 | 7186 | 1.14 | 4.61E-03 | 0.98 | 7.45E-01 | 1.09 | 8.88E-02 |
| TNF Superfamily Signaling | *TNIP1* | A_23_P19036 | 10318 | 1.08 | 7.85E-02 | 0.94 | 2.56E-01 | 1.04 | 3.95E-01 |
| TNF Superfamily Signaling | *TRAF5* | A_23_P201731 | 7188 | 1.08 | 2.63E-01 | 0.97 | 6.90E-01 | 1.03 | 7.08E-01 |
| TNF Superfamily Signaling | *TRAF1* | A_24_P89891 | 7185 | 1.17 | 3.69E-02 | 1.03 | 7.04E-01 | 1.18 | 3.13E-02 |
| TNF Superfamily Signaling | *TRAF3* | A_23_P37068 | 7187 | 1.09 | 2.24E-01 | 1.01 | 9.27E-01 | 1.11 | 1.45E-01 |
| TNF Superfamily Signaling | *TNF* | A_23_P376488 | 7124 | 0.93 | 4.55E-01 | 0.81 | 2.55E-02 | 0.92 | 3.55E-01 |
| TNF Superfamily Signaling | *TNFRSF17* | A_23_P37736 | 608 | 1.04 | 7.12E-01 | 0.94 | 5.58E-01 | 0.96 | 6.86E-01 |
| TNF Superfamily Signaling | *TNFRSF11A* | A_23_P390518 | 8792 | 0.94 | 4.43E-01 | 0.98 | 7.60E-01 | 0.89 | 1.45E-01 |
| TNF Superfamily Signaling | *TNFRSF12A* | A_23_P49338 | 51330 | 1.03 | 7.52E-01 | 1.10 | 2.66E-01 | 1.07 | 4.06E-01 |
| TNF Superfamily Signaling | *TNFRSF9* | A_23_P51936 | 3604 | 1.03 | 5.29E-01 | 1.05 | 3.00E-01 | 1.03 | 5.89E-01 |
| TNF Superfamily Signaling | *LTBR* | A_23_P53557 | 4055 | 1.09 | 6.58E-02 | 1.08 | 1.03E-01 | 1.04 | 4.40E-01 |
| TNF Superfamily Signaling | *TRADD* | A_23_P54649 | 8717 | 1.10 | 4.27E-02 | 1.01 | 8.60E-01 | 1.06 | 2.80E-01 |
| TNF Superfamily Signaling | *TNFSF9* | A_24_P5856 | 8744 | 0.88 | 2.40E-01 | 1.06 | 5.73E-01 | 0.97 | 7.96E-01 |
| TNF Superfamily Signaling | *TRAF6* | A_23_P75921 | 7189 | 1.02 | 6.81E-01 | 0.97 | 4.86E-01 | 0.99 | 7.76E-01 |
| TNF Superfamily Signaling | *TNFRSF13B* | A_23_P84705 | 23495 | 1.17 | 8.11E-02 | 0.92 | 3.33E-01 | 0.96 | 6.97E-01 |
| TNF Superfamily Signaling | *TNFRSF13C* | A_23_P91764 | 115650 | 1.04 | 5.46E-01 | 0.89 | 4.95E-02 | 0.90 | 7.90E-02 |
| TNF Superfamily Signaling | *TNFAIP3* | A_24_P166527 | 7128 | 1.02 | 8.60E-01 | 0.81 | 5.00E-02 | 0.97 | 7.93E-01 |
| TNF Superfamily Signaling | *TNFRSF11B* | A_24_P192485 | 4982 | 1.07 | 4.37E-01 | 1.20 | 1.98E-02 | 0.97 | 7.52E-01 |
| TNF Superfamily Signaling | *TNFSF14* | A_24_P237036 | 8740 | 0.93 | 1.86E-01 | 1.00 | 9.41E-01 | 0.96 | 4.52E-01 |
| TNF Superfamily Signaling | *TNFSF12* | A_24_P245298 | 8742 | 1.09 | 8.84E-03 | 1.05 | 1.73E-01 | 1.06 | 1.26E-01 |
| TNF Superfamily Signaling | *TNFRSF1B* | A_24_P54174 | 7133 | 1.02 | 6.76E-01 | 1.05 | 2.79E-01 | 1.07 | 1.06E-01 |
| TNF Superfamily Signaling | *TNFRSF4* | A_32_P26092 | 7293 | 1.04 | 3.14E-01 | 1.01 | 8.41E-01 | 0.99 | 7.90E-01 |
| Natural Killer Cell Signaling | *KIR3DL2* | A_23_P101636 | 3812 | 1.04 | 6.78E-01 | 0.94 | 5.67E-01 | 1.07 | 4.81E-01 |
| Natural Killer Cell Signaling | *PTPN11* | A_23_P99027 | 5781 | 1.04 | 2.36E-01 | 0.97 | 4.25E-01 | 1.03 | 4.13E-01 |
| Natural Killer Cell Signaling | *NCR1* | A_23_P108042 | 9437 | 1.08 | 2.81E-01 | 0.95 | 4.91E-01 | 1.05 | 4.97E-01 |
| Natural Killer Cell Signaling | *KLRC1* | A_23_P151046 | 3821 | 1.11 | 2.45E-01 | 0.93 | 4.64E-01 | 1.09 | 3.57E-01 |
| Natural Killer Cell Signaling | *PTPN6* | A_23_P162486 | 5777 | 1.10 | 1.88E-02 | 1.04 | 2.74E-01 | 1.06 | 1.69E-01 |
| Natural Killer Cell Signaling | *B3GAT1* | A_23_P1833 | 27087 | 1.18 | 8.13E-02 | 1.08 | 4.26E-01 | 1.16 | 1.30E-01 |
| Natural Killer Cell Signaling | *FCGR3A* | A_23_P200728 | 2214 | 1.13 | 7.48E-02 | 1.06 | 3.82E-01 | 0.98 | 8.02E-01 |
| Natural Killer Cell Signaling | *NCAM1* | A_23_P203053 | 4684 | 0.81 | 2.10E-02 | 0.94 | 5.05E-01 | 0.92 | 4.07E-01 |
| Natural Killer Cell Signaling | *KLRD1* | A_23_P204208 | 3824 | 1.12 | 1.05E-01 | 1.05 | 5.34E-01 | 1.15 | 5.05E-02 |
| Natural Killer Cell Signaling | *CD300A* | A_24_P159434 | 11314 | 1.02 | 7.97E-01 | 1.09 | 1.99E-01 | 1.11 | 1.65E-01 |
| Natural Killer Cell Signaling | *LAIR1* | A_24_P262688 | 3903 | 1.11 | 1.29E-01 | 1.05 | 4.97E-01 | 1.09 | 2.03E-01 |
| Natural Killer Cell Signaling | *KLRC4* | A_23_P218058 | 8302 | 1.05 | 4.79E-01 | 0.94 | 3.38E-01 | 0.92 | 2.90E-01 |
| Natural Killer Cell Signaling | *KLRC2* | A_23_P22232 | 3822 | 1.00 | 9.70E-01 | 0.88 | 2.59E-01 | 1.01 | 9.51E-01 |
| Natural Killer Cell Signaling | *NCR3* | A_23_P251881 | 259197 | 1.14 | 8.94E-02 | 1.01 | 9.32E-01 | 1.12 | 1.29E-01 |
| Natural Killer Cell Signaling | *MICA* | A_23_P257516 | 100507436 | 1.15 | 4.59E-02 | 1.13 | 1.05E-01 | 1.09 | 2.62E-01 |
| Natural Killer Cell Signaling | *TYROBP* | A_23_P27994 | 7305 | 1.15 | 1.58E-02 | 1.03 | 6.59E-01 | 1.07 | 2.68E-01 |
| Natural Killer Cell Signaling | *HLA-G* | A_23_P300112 | 3135 | 1.04 | 5.86E-01 | 0.89 | 8.72E-02 | 0.96 | 5.44E-01 |
| Natural Killer Cell Signaling | *HLA-E* | A_23_P30848 | 3133 | 1.05 | 3.28E-01 | 0.95 | 3.70E-01 | 1.04 | 4.50E-01 |
| Natural Killer Cell Signaling | *LILRB1* | A_23_P343221 | 10859 | 1.16 | 3.36E-02 | 1.15 | 4.91E-02 | 1.15 | 6.67E-02 |
| Natural Killer Cell Signaling | *CD160* | A_23_P354341 | 11126 | 1.04 | 6.39E-01 | 0.93 | 4.05E-01 | 1.00 | 9.77E-01 |
| Natural Killer Cell Signaling | *KIR3DL3* | A_23_P370574 | 115653 | 1.14 | 1.01E-01 | 0.95 | 4.93E-01 | 1.10 | 2.35E-01 |
| Natural Killer Cell Signaling | *MICB* | A_23_P387471 | 4277 | 1.12 | 1.72E-02 | 1.06 | 2.74E-01 | 1.12 | 2.09E-02 |
| Natural Killer Cell Signaling | *SIGLEC7* | A_23_P50175 | 27036 | 1.07 | 2.58E-01 | 1.05 | 5.07E-01 | 1.01 | 8.96E-01 |
| Natural Killer Cell Signaling | *NCR2* | A_23_P413224 | 9436 | 0.90 | 1.24E-01 | 0.96 | 5.49E-01 | 0.91 | 1.72E-01 |
| Natural Killer Cell Signaling | *SH2D1A* | A_24_P203103 | 4068 | 1.06 | 3.07E-01 | 1.00 | 9.65E-01 | 1.03 | 5.71E-01 |
| Natural Killer Cell Signaling | *KIR2DS4* | A_23_P78840 | 3809 | 1.04 | 6.82E-01 | 0.91 | 2.46E-01 | 1.05 | 6.05E-01 |
| Natural Killer Cell Signaling | *CD244* | A_24_P105332 | 51744 | 0.88 | 1.20E-01 | 1.01 | 9.48E-01 | 0.93 | 3.58E-01 |
| Natural Killer Cell Signaling | *KLRB1* | A_23_P99275 | 3820 | 1.03 | 6.31E-01 | 0.90 | 8.49E-02 | 1.00 | 9.76E-01 |
| Natural Killer Cell Signaling | *KLRK1* | A_24_P193093 | 22914 | 1.13 | 1.20E-01 | 0.94 | 5.39E-01 | 1.05 | 5.91E-01 |
| Complement Cascase | *C3* | A_23_P101407 | 718 | 1.08 | 1.08E-01 | 1.04 | 3.67E-01 | 1.10 | 3.69E-02 |
| Complement Cascase | *CFH* | A_23_P200160 | 3075 | 1.12 | 1.12E-01 | 1.08 | 2.94E-01 | 1.03 | 7.05E-01 |
| Complement Cascase | *CFD* | A_23_P119562 | 1675 | 1.21 | 2.22E-02 | 1.07 | 3.88E-01 | 1.19 | 4.34E-02 |
| Complement Cascase | *CR2* | A_24_P936376 | 1380 | 0.86 | 6.13E-02 | 1.06 | 4.21E-01 | 0.94 | 4.74E-01 |
| Complement Cascase | *C1R* | A_23_P125423 | 715 | 1.07 | 2.55E-01 | 0.95 | 3.09E-01 | 0.99 | 8.75E-01 |
| Complement Cascase | *C2* | A_32_P162183 | 717 | 1.24 | 1.76E-02 | 1.10 | 3.42E-01 | 1.29 | 5.68E-03 |
| Complement Cascase | *C1QB* | A_23_P137366 | 713 | 1.16 | 8.26E-02 | 1.11 | 2.40E-01 | 1.12 | 2.19E-01 |
| Complement Cascase | *C5AR1* | A_23_P303058 | 728 | 0.80 | 1.07E-02 | 0.91 | 2.58E-01 | 0.83 | 4.27E-02 |
| Complement Cascase | *CD46* | A_23_P201758 | 4179 | 1.04 | 4.57E-01 | 1.04 | 5.35E-01 | 1.11 | 8.08E-02 |

| | | | | | | | | | |
|---|---|---|---|---|---|---|---|---|---|
| Complement Cascase | *C8G* | A_23_P20713 | 733 | 0.99 | 8.90E-01 | 1.04 | 5.50E-01 | 0.94 | 4.00E-01 |
| Complement Cascase | *C7* | A_23_P213857 | 730 | 0.86 | 6.46E-02 | 1.09 | 2.95E-01 | 1.04 | 6.74E-01 |
| Complement Cascase | *SERPINA1* | A_23_P218111 | 5265 | 1.09 | 7.09E-02 | 1.07 | 1.52E-01 | 1.05 | 3.64E-01 |
| Complement Cascase | *C3AR1* | A_23_P2431 | 719 | 1.15 | 1.66E-02 | 1.10 | 8.73E-02 | 1.17 | 6.65E-03 |
| Complement Cascase | *C1S* | A_23_P2492 | 716 | 0.89 | 2.23E-01 | 0.93 | 4.43E-01 | 0.90 | 2.99E-01 |
| Complement Cascase | *CR1* | A_23_P256821 | 1378 | 1.10 | 1.22E-01 | 1.12 | 8.89E-02 | 1.06 | 3.93E-01 |
| Complement Cascase | *MASP2* | A_23_P301971 | 10747 | 0.87 | 1.08E-01 | 1.07 | 4.13E-01 | 0.95 | 5.56E-01 |
| Complement Cascase | *CD55* | A_23_P374862 | 1604 | 0.95 | 3.97E-01 | 0.91 | 1.67E-01 | 0.92 | 2.13E-01 |
| Complement Cascase | *C4B* | A_23_P42282 | 721 | 0.76 | 7.30E-03 | 0.90 | 3.15E-01 | 0.78 | 2.12E-02 |
| Complement Cascase | *C8A* | A_23_P46639 | 731 | 0.94 | 5.03E-01 | 0.93 | 3.52E-01 | 1.04 | 6.86E-01 |
| Complement Cascase | *CPAMD8* | A_23_P67198 | 27151 | 1.17 | 1.94E-03 | 1.01 | 9.17E-01 | 1.01 | 7.87E-01 |
| Complement Cascase | *C5* | A_23_P71855 | 727 | 0.96 | 4.37E-01 | 1.00 | 9.60E-01 | 0.95 | 3.60E-01 |
| Complement Cascase | *CD59* | A_24_P784765 | 966 | 0.94 | 4.42E-01 | 1.01 | 8.63E-01 | 0.97 | 7.30E-01 |
| Complement Cascase | *SERPINF2* | A_23_P89270 | 5345 | 0.86 | 7.60E-02 | 1.11 | 1.96E-01 | 0.88 | 1.60E-01 |
| Complement Cascase | *C4BPA* | A_23_P97541 | 722 | 1.03 | 7.70E-01 | 1.16 | 1.10E-01 | 1.14 | 1.82E-01 |
| Complement Cascase | *SERPINE1* | A_24_P158089 | 5054 | 0.92 | 2.26E-01 | 1.07 | 3.48E-01 | 1.01 | 9.34E-01 |
| Complement Cascase | *C1QA* | A_24_P222655 | 712 | 1.15 | 1.41E-01 | 1.10 | 3.42E-01 | 1.10 | 3.34E-01 |
| Complement Cascase | *C9* | A_32_P203917 | 735 | 0.82 | 8.52E-02 | 1.11 | 3.55E-01 | 1.00 | 9.70E-01 |
| ROS Glutathione Cytotoxic granules | *CAT* | A_23_P105138 | 847 | 1.00 | 9.58E-01 | 0.98 | 6.72E-01 | 1.02 | 5.25E-01 |
| ROS Glutathione Cytotoxic granules | *PRDX4* | A_23_P114232 | 10549 | 1.15 | 6.23E-03 | 1.01 | 8.22E-01 | 1.10 | 7.94E-02 |
| ROS Glutathione Cytotoxic granules | *GZMB* | A_23_P117602 | 3002 | 1.13 | 7.99E-02 | 1.02 | 8.28E-01 | 1.13 | 9.78E-02 |
| ROS Glutathione Cytotoxic granules | *PRDX1* | A_23_P11995 | 5052 | 1.06 | 8.58E-02 | 1.00 | 8.99E-01 | 1.05 | 1.34E-01 |
| ROS Glutathione Cytotoxic granules | *GZMH* | A_23_P128993 | 2999 | 1.30 | 9.65E-03 | 1.08 | 4.00E-01 | 1.23 | 4.30E-02 |
| ROS Glutathione Cytotoxic granules | *GZMM* | A_23_P130836 | 3004 | 1.06 | 6.69E-02 | 0.98 | 5.91E-01 | 1.00 | 9.75E-01 |
| ROS Glutathione Cytotoxic granules | *BPI* | A_23_P131785 | 671 | 1.30 | 2.92E-02 | 1.27 | 6.25E-02 | 1.24 | 9.03E-02 |
| ROS Glutathione Cytotoxic granules | *GZMA* | A_23_P133445 | 3001 | 1.12 | 7.77E-02 | 1.00 | 9.66E-01 | 1.09 | 2.00E-01 |
| ROS Glutathione Cytotoxic granules | *GPX3* | A_23_P133474 | 2878 | 1.01 | 8.35E-01 | 1.02 | 7.70E-01 | 0.96 | 5.35E-01 |
| ROS Glutathione Cytotoxic granules | *SOD2* | A_23_P134176 | 6648 | 1.04 | 4.37E-01 | 0.98 | 7.14E-01 | 1.05 | 3.78E-01 |
| ROS Glutathione Cytotoxic granules | *NCF2* | A_23_P138194 | 4688 | 1.05 | 4.55E-01 | 1.10 | 2.30E-01 | 1.01 | 8.47E-01 |
| ROS Glutathione Cytotoxic granules | *PRDX2* | A_24_P168416 | 7001 | 0.91 | 1.29E-01 | 1.06 | 3.01E-01 | 0.95 | 4.15E-01 |
| ROS Glutathione Cytotoxic granules | *PRTN3* | A_23_P142345 | 5657 | 0.95 | 3.48E-01 | 1.02 | 7.87E-01 | 0.90 | 7.06E-02 |
| ROS Glutathione Cytotoxic granules | *PRF1* | A_23_P1473 | 5551 | 1.13 | 7.57E-02 | 1.04 | 5.71E-01 | 1.12 | 9.59E-02 |
| ROS Glutathione Cytotoxic granules | *SOD1* | A_23_P154840 | 6647 | 1.04 | 3.31E-01 | 0.97 | 4.36E-01 | 1.04 | 2.80E-01 |
| ROS Glutathione Cytotoxic granules | *CYBB* | A_24_P365767 | 1536 | 1.13 | 4.23E-02 | 1.05 | 4.43E-01 | 1.11 | 7.53E-02 |
| ROS Glutathione Cytotoxic granules | *GPX2* | A_23_P3038 | 2877 | 0.83 | 5.04E-02 | 0.99 | 9.05E-01 | 0.89 | 2.42E-01 |
| ROS Glutathione Cytotoxic granules | *ANPEP* | A_23_P88626 | 290 | 1.04 | 6.11E-01 | 1.08 | 2.70E-01 | 1.12 | 1.17E-01 |
| Glucocorticoid PPAR signaling | *NR4A1* | A_23_P128230 | 3164 | 0.88 | 4.02E-01 | 0.70 | 1.19E-02 | 0.88 | 4.24E-01 |
| Glucocorticoid PPAR signaling | *FKBP4* | A_23_P128372 | 2288 | 0.89 | 1.11E-02 | 1.06 | 2.38E-01 | 1.02 | 7.29E-01 |
| Glucocorticoid PPAR signaling | *NR4A2* | A_23_P131208 | 4929 | 0.86 | 3.26E-01 | 0.69 | 1.10E-02 | 0.93 | 6.21E-01 |
| Glucocorticoid PPAR signaling | *GMEB1* | A_24_P621023 | 10691 | 1.10 | 4.20E-02 | 1.03 | 5.08E-01 | 1.11 | 2.51E-02 |
| Glucocorticoid PPAR signaling | *NRIP1* | A_23_P211007 | 8204 | 1.06 | 4.49E-01 | 0.94 | 4.69E-01 | 1.03 | 6.87E-01 |
| Glucocorticoid PPAR signaling | *NR3C1* | A_24_P214754 | 2908 | 1.07 | 6.45E-02 | 0.98 | 6.82E-01 | 1.03 | 4.20E-01 |
| Glucocorticoid PPAR signaling | *CITED2* | A_23_P214969 | 10370 | 1.12 | 4.25E-03 | 1.06 | 1.15E-01 | 1.07 | 1.21E-01 |
| Glucocorticoid PPAR signaling | *PPARA* | A_24_P417036 | 5465 | 1.07 | 2.43E-01 | 1.01 | 8.45E-01 | 1.09 | 1.23E-01 |
| Glucocorticoid PPAR signaling | *GLCCI1* | A_23_P336198 | 113263 | 1.09 | 1.25E-01 | 1.01 | 8.67E-01 | 1.04 | 5.46E-01 |
| Glucocorticoid PPAR signaling | *GMEB2* | A_23_P337033 | 26205 | 0.78 | 1.22E-02 | 1.11 | 2.87E-01 | 0.92 | 4.20E-01 |
| Glucocorticoid PPAR signaling | *NR2F1* | A_23_P348737 | 7025 | 0.81 | 1.56E-02 | 1.00 | 9.63E-01 | 0.91 | 3.29E-01 |
| Glucocorticoid PPAR signaling | *KPNA1* | A_23_P351055 | 3836 | 0.97 | 4.38E-01 | 0.98 | 7.36E-01 | 1.01 | 8.15E-01 |
| Glucocorticoid PPAR signaling | *NCOA1* | A_23_P39602 | 8648 | 1.02 | 5.86E-01 | 0.95 | 1.68E-01 | 0.97 | 3.95E-01 |
| Glucocorticoid PPAR signaling | *SDPR* | A_23_P72668 | 8436 | 1.05 | 5.39E-01 | 1.07 | 4.06E-01 | 1.25 | 6.65E-03 |
| Glucocorticoid PPAR signaling | *FAF1* | A_23_P96853 | 11124 | 1.08 | 7.15E-03 | 0.96 | 1.64E-01 | 1.00 | 9.62E-01 |
| Glucocorticoid PPAR signaling | *NCOA2* | A_24_P166794 | 10499 | 1.01 | 7.26E-01 | 1.02 | 6.28E-01 | 1.05 | 2.57E-01 |
| Glucocorticoid PPAR signaling | *HSPD1* | A_32_P76091 | 3329 | 1.09 | 8.07E-02 | 0.96 | 3.78E-01 | 1.09 | 1.01E-01 |
| Calcium Signaling | *PPP3R1* | A_23_P108592 | 5534 | 0.92 | 1.06E-01 | 1.02 | 6.58E-01 | 0.95 | 3.50E-01 |
| Calcium Signaling | *CAMK1D* | A_23_P124252 | 57118 | 1.07 | 1.17E-01 | 1.09 | 5.08E-02 | 0.99 | 7.66E-01 |
| Calcium Signaling | *NFATC4* | A_23_P140394 | 4776 | 0.98 | 6.68E-01 | 1.00 | 9.78E-01 | 0.95 | 2.90E-01 |
| Calcium Signaling | *PPP3CC* | A_23_P157495 | 5533 | 1.11 | 4.27E-02 | 0.98 | 6.55E-01 | 1.07 | 1.98E-01 |
| Calcium Signaling | *CAMK4* | A_23_P250347 | 814 | 1.05 | 2.60E-01 | 0.99 | 8.00E-01 | 1.01 | 7.71E-01 |
| Calcium Signaling | *NFATC1* | A_24_P258846 | 4772 | 0.98 | 6.71E-01 | 1.01 | 7.61E-01 | 0.99 | 8.48E-01 |
| Calcium Signaling | *NFAT5* | A_23_P359647 | 10725 | 1.03 | 4.87E-01 | 0.98 | 6.46E-01 | 1.04 | 3.56E-01 |
| Calcium Signaling | *NFATC3* | A_24_P373312 | 4775 | 1.11 | 1.40E-02 | 0.98 | 5.89E-01 | 1.02 | 5.84E-01 |
| Calcium Signaling | *NFATC2* | A_24_P525917 | 4773 | 1.07 | 1.95E-01 | 0.96 | 4.57E-01 | 1.02 | 7.65E-01 |
| Calcium Signaling | *PPP3CA* | A_23_P92623 | 5530 | 1.07 | 1.10E-01 | 0.95 | 2.26E-01 | 1.00 | 9.23E-01 |
| Calcium Signaling | *PPP3R2* | A_23_P9348 | 5535 | 0.84 | 3.85E-03 | 1.02 | 7.56E-01 | 1.03 | 6.76E-01 |
| Calcium Signaling | *CABIN1* | A_24_P102512 | 23523 | 1.13 | 2.90E-02 | 0.91 | 1.29E-01 | 1.06 | 3.24E-01 |
| Calcium Signaling | *PPP3CB* | A_24_P98524 | 5532 | 1.09 | 2.51E-02 | 1.01 | 8.95E-01 | 1.04 | 3.35E-01 |

**Table S 2.** Number of unique genes by sub-pathways in the inflammation panel from the inflammatory transcriptome by Loza et al. and in our study. Percentage of gene present in our dataset is also given.

| Primary subpathways | Number of genes in subpathways (Loza etal) | Number of genes in subpathways (EGM) | Percentage (EGM) |
|---|---|---|---|
| Adhesion-Extravasation-Migration | 142 | 110 | 77.5 |
| Apoptosis Signaling | 68 | 64 | 94.1 |
| Calcium Signaling | 14 | 13 | 92.9 |
| Complement Cascase | 40 | 27 | 67.5 |
| Cytokine signaling | 172 | 119 | 69.2 |
| Eicosanoid Signaling | 39 | 32 | 82.1 |
| Glucocorticoid/PPAR signaling | 21 | 17 | 81 |
| G-Protein Coupled Receptor Signaling | 42 | 34 | 81 |
| Innate pathogen detection | 50 | 34 | 68 |
| Leukocyte signaling | 121 | 103 | 85.1 |
| MAPK signaling | 118 | 111 | 94.1 |
| Natural Killer Cell Signaling | 31 | 29 | 93.5 |
| NF-kB signaling | 33 | 32 | 97 |
| Phagocytosis-Ag presentation | 39 | 37 | 94.9 |
| PI3K/AKT Signaling | 37 | 34 | 91.9 |
| ROS/Glutathione/Cytotoxic granules | 22 | 18 | 81.8 |
| TNF Superfamily Signaling | 38 | 31 | 81.6 |
| **Inflammatory transcriptome** | **1027** | **845** | **82.3** |

**Table S 3.** List of genes present in our study for each inflammatory transcriptome sub-pathways.

| Primary subpathways | Gene ID |
|---|---|
| Adhesion-Extravasation-Migration | *ADAM8, ALCAM, ALS2, ARHGAP1, CCBP2, CCL15, CCL17, CCL19, CCL2, CCL21, CCL23, CCL24, CCL27, CCL28, CCL4, CCL5, CCR1, CCR2, CCR3, CCR4, CCR5, CCR6, CCR7, CCRL2, CD2, CD226, CD33, CD36, CD47, CD48, CD58, CD9, CD96, CD97, CD99, CEACAM1, CEACAM3, CEACAM8, CKLF, CRK, CTNND1, CTTN, CX3CL1, CX3CR1, CXCL1, CXCL12, CXCL16, CXCL2, CXCL3, CXCL5, CXCL9, CXCR6, DARC, F11R, FUT4, FYB, HMMR, ICAM1, ICAM2, ICAM3, ICAM5, ITGA1, ITGA2, ITGA2B, ITGA4, ITGA5, ITGA6, ITGAD, ITGAE, ITGAL, ITGAM, ITGAV, ITGAX, ITGB1, ITGB2, ITGB3, ITGB4, ITGB7, JAM2, JAM3, MIF, MMP1, MMP14, MMP19, MMP9, MUC1, MYH10, MYH9, MYL6, NT5E, PECAM1, PLAUR, PPBP, PTPRU, PXN, RASSF5, RHOA, RHOH, ROCK2, SELL, SELP, SELPLG, SIGLEC1, SIPA1, SPN, THY1, VASP, VCL, XCL1, XCR1* |
| Apoptosis Signaling | *ACIN1, APAF1, BAD, BAK1, BAX, BBC3, BCL2, BCL2A1, BCL2L1, BCL2L11, BCL2L12, BCL2L13, BCL2L14, BCL2L2, BID, BIK, BIRC2, BIRC3, CAPN1, CAPN10, CAPN2, CAPNS1, CASP10, CASP14, CASP2, CASP3, CASP6, CASP7, CASP8, CASP8AP2, CASP9, CFLAR, CRADD, CYCS, DAP, DAP3, DAPK1, DAXX, DFFA, DFFB, DIABLO, ENDOG, FADD, FAIM, FAS, FASLG, GAS2, HTRA2, LMNA, MCL1, PARP1, PTPN13, RIPK2, RIPK3, ROCK1, SPTAN1, TANK, TNFRSF10A, TNFRSF10B, TNFRSF10C, TNFRSF10D, TNFRSF21, TNFSF10, TP53* |
| Calcium Signaling | *CABIN1, CAMK1D, CAMK4, NFAT5, NFATC1, NFATC2, NFATC3, NFATC4, PPP3CA, PPP3CB, PPP3CC, PPP3R1, PPP3R2* |
| Complement Cascase | *C1QA, C1QB, C1R, C1S, C2, C3, C3AR1, C4B, C4BPA, C5, C5AR1, C7, C8A, C8G, C9, CD46, CD55, CD59, CFD, CFH, CPAMD8, CR1, CR2, MASP2, SERPINA1, SERPINE1, SERPINF2* |
| Cytokine signaling | *ACE, ADAM17, AGER, BMPR1A, BMPR2, CASP1, CEBPB, CISH, CRP, CSF1, CSF1R, CSF2RB, CSF3R, EBI3, ENG, GATA3, HMGB1, IFNA4, IFNAR1, IFNAR2, IFNB1, IFNG, IFNGR1, IFNGR2, IL10, IL10RA, IL10RB, IL12RB1, IL12RB2, IL13RA1, IL15, IL15RA, IL16, IL17A, IL17B, IL17F, IL17RA, IL18, IL18R1, IL18RAP, IL19, IL1B, IL1F10, IL1F6, IL1F7, IL1R1, IL1R2, IL1RAP, IL1RL2, IL1RN, IL21R, IL23R, IL24, IL27RA, IL28A, IL29, IL2RA, IL2RB, IL2RG, IL31RA, IL32, IL3RA, IL4, IL4R, IL5RA, IL6, IL6R, IL6ST, IL7, IL7R, IL8, ILF2, IRF1, IRF2, IRF3, IRF4, IRF5, IRF7, IRF8, JAK1, JAK2, JAK3, LIF, NFIL3, NMI, OSM, PDGFB, PDGFRB, PIAS1, PIAS2, PIAS3, PIAS4, PTPN1, PTPN2, S100A12, S100A8, S100A9, SOCS1, SOCS2, SOCS3, SOCS4, SOCS5, SOCS6, STAT1, STAT2, STAT3, STAT4, STAT5A, STAT5B, STAT6, TBX21, TGFB1, TGFB3, TGFBR1, TGFBR2, TGIF2, THPO, TXLNA, TYK2* |
| Eicosanoid Signaling | *AKR1C3, ALOX12, ALOX12B, ALOX15, ALOX15B, ALOX5, ALOX5AP, CYSLTR1, CYSLTR2, DPEP1, DPEP2, DPEP3, GGT1, GPR44, LTA4H, LTC4S, MGST2, MGST3, PLA2G2A, PLA2G2D, PTGDR, PTGDS, PTGER2, PTGER3, PTGER4, PTGES2, PTGES3, PTGIR, PTGS1, PTGS2, TBXA2R, TBXAS1* |
| Glucocorticoid/PPAR signaling | *CITED2, FAF1, FKBP4, GLCCI1, GMEB1, GMEB2, HSPD1, KPNA1, NCOA1, NCOA2, NR2F1, NR3C1, NR4A1, NR4A2, NRIP1, PPARA, SDPR* |
| G-Protein Coupled Receptor Signaling | *ADCY1, ADCY2, ADCY4, ADORA1, ADORA2A, ADORA2B, ADORA3, ADRA2A, ADRA2C, ADRB2, ADRBK1, CREB1, CREBBP, CREM, HRH2, PDE1A, PDE1B, PDE2A, PDE3B, PDE4A, PDE4B, PDE4C, PDE4D, PLCB1, PLCB2, PRKACA, PRKACB, PRKACG, PRKAR1A, PRKAR2A, PRKAR2B, PTK2B, RGS1, SYNGAP1* |
| Innate pathogen detection | *CARD6, CARD8, CASP5, CD14, CD180, DDX58, HSP90B1, IFIH1, IRAK1, IRAK2, IRAK3, IRAK4, LY96, MYD88, OAS1, OAS2, PGLYRP1, PGLYRP3, PRKRA, PYCARD, SARM1, TICAM1, TIRAP, TLR1, TLR10, TLR2, TLR3, TLR4, TLR5, TLR6, TLR7, TLR8, TLR9, TOLLIP* |
| Leukocyte signaling | *ABL1, BLNK, BTK, CBL, CD22, CD28, CD37, CD38, CD3E, CD4, CD40, CD40LG, CD44, CD5, CD52, CD53, CD68, CD79A, CD79B, CD80, CD86, CD8A, CSK, CST7, CTLA4, DAPP1, DPP4, EDNRA, FCAR, FCER1A, FCER1G, FCER2, FCGR2A, FCGR2B, FLT3, FLT3LG, FOXP3, FYN, GRAP2, GRB2, ICOS, ICOSLG, IGHA1, IGLL1, ITK, LAT, LCK, LCP2, LILRA1, LILRA2, LILRA3, LILRA4, LILRA5, LILRB2, LILRB3, LILRB4, LILRP2, LRRC23, MAL, MARCO, MS4A1, MS4A2, MST1R, PAG1, PAX5, PIK3AP1, PILRB, PTAFR, PTPN22, PTPN7, PTPRC, PTPRJ, SCARF1, SCARF2, SCGB1A1, SCGB3A1, SECTM1, SEMA4D, SH2D1B, SH3BP2, SHB, SIGLEC10, SIGLEC5, SIRPB1, SIRPG, SLA2, SLAMF1, SLAMF6, SLAMF7, SLAMF9, SLC3A2, SLC7A5, SYK, TACR1, TCF7, TCIRG1, TEC, TFEB, TREM1, TXK, VAV1, VPREB1, ZAP70* |
| MAPK signaling | *ARAF, ATF1, ATF2, ATF3, ATF4, BCAR1, BRAF, DDIT3, DUSP1, DUSP10, DUSP2, DUSP4, DUSP6, DUSP9, EEF2K, EGF, ELK1, EP300, ESR1, ETS1, ETS2, FOS, FOSL1, HDAC1, HDAC2, HINT1, HMGN1, HRAS, HSPB1, IFI16, INS, JUN, JUND, KCNH8, KRAS, KSR1, LYN, MADD, MAP2K1, MAP2K2, MAP2K3, MAP2K4, MAP2K6, MAP3K1, MAP3K5, MAP3K6, MAP3K7, MAP4K1, MAPK1, MAPK11, MAPK12, MAPK13, MAPK14, MAPK3, MAPK8, MAPK9, MAPKAPK2, MAPKAPK3, MAPKAPK5, MAX, MEF2A, MEF2C, MEF2D, MKNK1, MYC, MYCN, NRAS, PAK1, PLA2G4A, PLCG1, PPM1A, PPP1CA, PPP1CB, PPP1CC, PPP1R7, PPP2CA, PPP2CB, PPP2R1A, PPP2R1B, PPP2R2B, PPP2R3A, PRKCA, PRKCD, PRKCE, PRKCQ, PRKCZ, PTK2, PTPRK, RAC1, RAC2, RAF1, RAP1A, RAP1GAP, RAPGEF1, RAPGEF3, RASA1, RASGRP1, RPS6KA1, RPS6KA4, RPS6KA5, SHC1, SOS1, SOS2, SRC, TLN1, TLN2, YWHAB, YWHAG, YWHAH, YWHAQ, YWHAZ* |
| Natural Killer Cell Signaling | *B3GAT1, CD160, CD244, CD300A, FCGR3A, HLA-E, HLA-G, KIR2DS4, KIR3DL2, KIR3DL3, KLRB1, KLRC1, KLRC2, KLRC4, KLRD1, KLRK1, LAIR1, LILRB1, MICA, MICB, NCAM1, NCR1, NCR2, NCR3, PTPN11, PTPN6, SH2D1A, SIGLEC7, TYROBP* |
| NF-kB signaling | *BCL10, BCL3, BCL6, BTRC, CARD10, CARD11, CARD14, CARD9, CHUK, CSNK2A1, CSNK2A2, CSNK2B, EIF2AK2, IKBKB, IKBKG, MALT1, MAP3K14, MAP3K3, MEFV, NFKB1, NFKB2, NFKBIA, NFKBIB, NFKBIE, NFRKB, REL, RELA, RELB, RIPK1, TBK1, UBE2N, UBE2V1* |
| Phagocytosis-Ag presentation | *CD1A, CD1B, CD1C, CD1D, CD209, CD74, CIITA, CTSS, HLA-A, HLA-B, HLA-C, HLA-DMA, HLA-DMB, HLA-DOA, HLA-DPA1, HLA-DPB1, HLA-DQA1, HLA-DQA2, HLA-DQB2, HLA-DRA, IFI30, LAG3, LILRB5, NFX1, PRSS16, PSMA1, PSMB5, PSMB9, PSME1, PSME2, RFX1, RFX4, RFXANK, TAP1, TAP2, TAPBP, XBP1* |
| PI3K/AKT Signaling | *AKT1, AKT2, AKT3, CCND1, CDC37, CDKN1A, CDKN1B, CTNNB1, EIF4E, GAB1, HSP90AA1, HSP90AB1, ILK, INPP5D, INPPL1, LIMS1, MAP3K8, MDM2, NOS3, PDPK1, PIK3CA, PIK3CB, PIK3CD, PIK3R1, PIK3R2, PIK3R3, PIK3R5, PTEN, RHEB, RPS6KB1, RPS6KB2, THEM4, TSC1, TSC2* |
| ROS/Glutathione/Cytotoxic granules | *ANPEP, BPI, CAT, CYBB, GPX2, GPX3, GZMA, GZMB, GZMH, GZMM, NCF2, PRDX1, PRDX2, PRDX4, PRF1, PRTN3, SOD1, SOD2* |
| TNF Superfamily Signaling | *LTA, LTBR, PTX3, TNF, TNFAIP3, TNFAIP6, TNFRSF11A, TNFRSF11B, TNFRSF12A, TNFRSF13B, TNFRSF13C, TNFRSF14, TNFRSF17, TNFRSF1A, TNFRSF1B, TNFRSF25, TNFRSF4, TNFRSF9, TNFSF12, TNFSF13B, TNFSF14, TNFSF4, TNFSF8, TNFSF9, TNIP1, TRADD, TRAF1, TRAF2, TRAF3, TRAF5, TRAF6* |

**Scientists participating in the project consortium:**

**National Hellenic Research Foundation, Athens:**
Soterios A. Kyrtopoulos, Panagiotis Georgiadis, Maria Botsivali, Christina Papadopoulou, Aristotelis Chatziioannou, Ioannis Valavanis

**University of Maastricht:**
Jos C.S. Kleinjans, Theo M.C.M. de Kok, Dennie G.A.J. Hebels, Ralph Gottschalk, Danitsja van Leeuwen, Leen Timmermans

**Imperial College London , London:**
Paolo Vineis, Hector C. Keun, Toby J. Athersuch, Marc Chadeau-Hyam, Rachel Kelly, Gianluca Campanella

**Umea University:**
Ingvar A. Bergdahl, Goran Hallmans, Beatrice Melin, Per Lenner

**Istituto per lo Studio e la Prevenzione Oncologica, Florence:**
Domenico Palli, Benedetta Bendinelli

**University of Crete:**
Euripides G. Stephanou, Antonis Myridakis, Manolis Kogevinas

**University of Utrecht:**
Roel Vermeulen, Lutzen Portengen, Fatemeh Saberi-Hosnijeh

**Istituto Superiore di Sanita , Rome:**
Lucia Fazzo, Marco De Santis, Pietro Comba

**National Institute for Health and Welfare, Kuopio:**
Hannu Kiviranta, Panu Rantakokko, Riikka Airaksinen, Paivi Ruokojarvi

**University of Leeds :**
Mark Gilthorpe, Sarah Fleming, Thomas Fleming, Yu-Kang Tu

**Lund University :**
Bo Jonsson, Thomas Lundh

**National Taiwan University :**
Kuo-Liong Chien, Wei J. Chen, Wen-Chung Lee, Chuhsing Kate Hsiao, Po-Hsiu Kuo, Hung Hung, Shu-Fen Liao
